# Face-on crystallization in ambipolar organic mixed conductors enables homogeneous light-modulated artificial neurons

**DOI:** 10.1093/nsr/nwag341

**Published:** 2026-06-09

**Authors:** Yulin Zhang, Shijie Wang, Guangyu Qi, Xinmei Cai, Sen Zhang, Bingjun Wang, Chao Zhao, Yong Han, Qunping Fan, Laili Wang, Wei Ma

**Affiliations:** State Key Laboratory for Mechanical Behavior of Materials, Xi’an Jiaotong University, Xi’an 710049, China; State Key Laboratory for Mechanical Behavior of Materials, Xi’an Jiaotong University, Xi’an 710049, China; State Key Laboratory for Mechanical Behavior of Materials, Xi’an Jiaotong University, Xi’an 710049, China; State Key Laboratory for Mechanical Behavior of Materials, Xi’an Jiaotong University, Xi’an 710049, China; State Key Laboratory for Mechanical Behavior of Materials, Xi’an Jiaotong University, Xi’an 710049, China; State Key Laboratory for Mechanical Behavior of Materials, Xi’an Jiaotong University, Xi’an 710049, China; State Key Laboratory for Mechanical Behavior of Materials, Xi’an Jiaotong University, Xi’an 710049, China; State Key Laboratory for Mechanical Behavior of Materials, Xi’an Jiaotong University, Xi’an 710049, China; State Key Laboratory for Mechanical Behavior of Materials, Xi’an Jiaotong University, Xi’an 710049, China; State Key Laboratory of Electrical Insulation and Power Equipment, Xi’an Jiaotong University, Xi’an 710049, China; State Key Laboratory for Mechanical Behavior of Materials, Xi’an Jiaotong University, Xi’an 710049, China

**Keywords:** artificial neuron, organic mixed ionic-electronic conductors, ambipolar semiconductors, molecular weight, optic nerve

## Abstract

Light-modulated artificial neurons are vital for clinical optic nerve repair and bionic robotics. Light-sensitive ambipolar organic mixed ionic-electronic conductors (OMIECs) allow for homogeneously integrated artificial neurons, free from issues such as voltage/resistance mismatch or digital-to-analog conversion. However, their development is constrained by a trade-off between ion and electron transport, which results in low figure-of-merit (μC*) values and P/N-mode imbalance. Here, through precise molecular weight tailoring, we develop an ambipolar OMIEC with a high face-on orientation ratio of 85.5% and a 4-fold enhancement in backbone crystallinity. This approach simultaneously enhances vertical ionic transport and electron transport, enabling the synergetic optimization of electronic mobility and volumetric capacitance. The optimized material achieves high μC* values of 423.3 ± 17.3 F cm^−1^ V^−1^ s^−1^ (N-type) and 359.8 ± 42.6 F cm^−1^ V^−1^ s^−1^ (P-type). Using this OMIEC, we construct a homogeneous artificial neuron whose excitation/inhibition behaviors can be dually modulated by light and chemical environment, paving new paths for high-performance OMIECs in biological neural prosthetics and bionic machine vision systems.

## Teaser

Precise control of molecular orientation and crystallinity yields an ambipolar organic mixed conductor with optimized electronic and ionic transport, enabling homogeneously integrated artificial optic neurons for bionic vision.

## INTRODUCTION

Artificial neural circuits (ANCs), featuring fused signal perception and frequency encoding function, show unique promise for clinical nerve repair and bionic robotics, creating new opportunities in life sciences and human-machine interaction [1–3]. Compared to traditional silicon-based or phase change materials-based circuits, organic mixed ionic-electronic conductor (OMIEC)-based ANCs represent a revolutionary breakthrough [4,5]. Their intrinsic chemical sensitivity, mechanical flexibility and biocompatibility enable efficient, conformal and safe integration with biological tissues [6–8]. For instance, recent studies have demonstrated the successful application of OMIEC-based ANCs in chemically mediated biological rhythm regulation [9–11], nerve function replacement [6,12], and anticoincidence detection [13–15]. However, current OMIEC-based ANC technologies face significant limitations: all reported OMIEC-based ANCs rely on organic electrochemical transistor (OECT)-based P/N complementary logic circuits and external heterogeneous integration with inorganic sensors (i.e. artificial receptors). This heterogeneous integration approach, although effective, can suffer from the following challenges [16]: (1) High manufacturing complexity and low integration density, which arise from the sequential deposition and patterning process of P/N-type materials [17–19]. (2) The resistance and supply voltage of inorganic sensors and organic neurons can be mismatched, which limits the performance and increases the power consumption of ANCs. (3) The integration with rigid and bulky external sensors leads to poor mechanical flexibility and biocompatibility. In contrast, constructing a homogeneous ANC based on a single OMIEC is a viable solution to fundamentally overcome these inherent drawbacks [20,21].

The single-component nature of ambipolar OMIECs avoids the need for the complex material systems of conventional P/N-type complementary circuits [22–25]. Moreover, recent works demonstrated their potential use in multimodal sensing, including electric field, chemical pressure and light [26,27]. However, their performance is still significantly lagging behind unipolar OMIECs, which limits their application in homogeneous ANCs. This issue mainly originates from the fundamental trade-off between electronic transport (influencing electronic mobility, *μ*) and ionic transport (influencing volumetric capacitance, C* and response time, *τ*) characteristics [27–31]. Recent research has focused on two primary strategies: backbone/side-chain engineering and a binary blending approach. The former aims to enhance performance via backbone and side-chain optimization to improve molecular packing, energy levels, and orbital properties [27,30,32–34]. The latter seeks to optimize device performance by blending complementary P-type and N-type OMIECs [35,36]. While these approaches have yielded some improvements, they have not yet overcome the core issues caused by the electronic-ionic transport trade-off, resulting in a limited overall figure-of-merit (μC* < 250 F cm^−1^ V^−1^ s^−1^ for the inferior operation mode). Beyond performance, the operational stability of ambipolar OMIECs under long-term use remains a critical challenge for the practical applications of ANCs. Besides, the multimodal sensing capability of these ambipolar OMIECs is not fully demonstrated, impeding the construction of homogeneous ANCs with fused sensing-processing function.

Here, we introduced a strategy to control molecular weight by controlling the polymerization time of 2,5-di(2,5,8,11,14-pentaoxahexadecan-16-yl)-3,6-di(thiophen-2-yl)-2,5-dihydropyrrolo[3,4-c]pyrrole-1,4-dione (HOMO-gDPP), an ambipolar OMIEC with engineered HOMO/LUMO levels [37]. We demonstrated that controlling molecular weight can effectively regulate the molecular stacking orientation and order degree of the polymer backbone. This leads to two key effects: (1) The increased backbone order degree extends the π-π conjugated length, significantly improving both hole/electron transport mobility (from *μ*_h_/*μ*_e _= 0.76 ± 0.03/1.76 ± 0.12 cm^2^ V^−1^ s^−1^ to 1.34 ± 0.09/2.24 ± 0.11 cm^2^ V^−1^ s^−1^). (2) The face-on orientation proportion increases from 69.3% to 85.5%, which provides continuous vertical ion transport pathways that substantially accelerate out-of-plane ion transport, resulting in improved C* and fast response. These improvements collectively overcome the electronic–ionic transport trade-off, achieving high μC* values of 423.3 ± 17.3 F cm^−1^ V^−1^ s^−1^ (N-type) and 359.8 ± 42.6 F cm^−1^ V^−1^ s^−1^ (P-type). The optimized molecular stacking also enhances operational stability and sensitivity to light stimuli. Using this high-performance ambipolar OMIEC, we demonstrate the first flexible optical ANC with homogeneously integrated photoreceptors and axon–hillock neurons. This novel ANC exhibits a stable dual light/chemical response, successfully emulating the light-induced inhibitory and excitatory behaviors of the biological retina, and offers promise for biological nerve replacement and machine vision systems.

## RESULTS AND DISCUSSION

**Figure 1.**
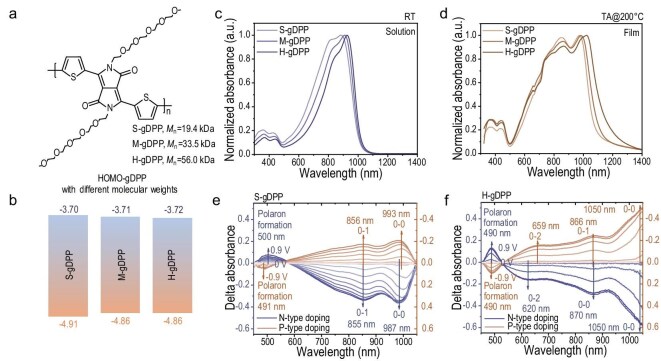
Molecular structure and optoelectronic characterization of HOMO-gDPP. (a) Molecular structure of HOMO-gDPP. (b) Schematic illustration of LUMO/HOMO energy level diagrams for HOMO-gDPP with different molecular weights. (c) UV-Vis absorption spectra of three HOMO-gDPP materials with different molecular weights in chloroform solution. (d) UV-Vis absorption spectra of thin films for the three HOMO-gDPP materials with varying molecular weights. (e and f) UV-Vis absorption spectra of S-gDPP (e) and H-gDPP (f) during the electrochemical doping process in 1 M NaCl aqueous solution. Notably, all thin‑film samples in (d–f) were annealed at 200°C before the test.

To synthesize HOMO-gDPP with varying molecular weights, we adjusted polymerization time, successfully synthesized three distinct HOMO-gDPP polymers. The synthesis details and nuclear magnetic resonance information can be found in Figs S1–S10 and Note S1. Gel permeation chromatography (GPC) shows that the three polymers have different molecular weights (Fig. S11 and Table S1): S-gDPP: *M*_n _= 19.4 kDa (PDI = 1.6), M-gDPP: *M*_n _= 33.5 kDa (PDI = 1.3), and H-gDPP: *M*_n _= 56.0 kDa (PDI = 1.1). Cyclic voltammetry (CV, Fig. S12) measurements revealed the LUMO/HOMO energy levels of the three polymers are slightly different: S-gDPP: −3.70/−4.91 eV, M-gDPP: −3.71/−4.86 eV, and H-gDPP: −3.72/−4.86 eV (Fig. 1b). This result indicates that an increase in molecular weight can achieve a narrower energy band gap with deeper the LUMO level and a shallower HOMO level, thereby facilitating the ambipolar electrochemical doping and charge transport. We next performed UV-Vis absorption spectroscopy in both solution (chloroform) and thin-film states to characterize the impact of molecular weight on optical properties [38]. Spectral analysis (Fig. 1c and d) revealed that the aggregation behavior of the three polymers displayed significant molecular weight dependence. In the solution state, the absorption edge on the high-energy side progressively redshifted from 886 nm for S-gDPP to 910 nm for M-gDPP and 926 nm for H-gDPP, attributed to enhanced polymer chain aggregation[39]. Additionally, the M-gDPP and H-gDPP thin-film samples exhibited an obvious redshifted absorption edge on the low-energy side and a higher 0–0/0–1 intensity ratio, which coincides well with the CV measurement, suggesting a narrower band gap. This change can originate from a more planar backbone conformation in the solid state due to stronger intramolecular interactions [40].

*In situ* spectroelectrochemical measurements (Fig. 1e, f and Figs S13–S16) demonstrated ambipolar doping/dedoping characteristics under applied voltages (0–0.9 V for N-type doping/dedoping, and 0 to −0.9 V for P-type doping/dedoping), with all samples showing polaron absorption peaks (450–550 nm) and ground state bleaching at 900–1050 nm (0–0) and 800–900 nm (0–1). Notably, H-gDPP exhibited a distinct 0–2 transition peak at around 650 nm, indicative of enriched electronic states at higher molecular weights [41]. The spectra return to their original state after dedoping, indicating that the doping/dedoping process is highly reversible for all three polymers. Quantitative analysis (Figs S14 and S16) revealed that increasing molecular weights can significantly improve electrochemical doping efficiency in both P-type and N-type modes, as evidenced by the more rapid absorption intensity changes with doping voltage.

The planar OECT devices based on HOMO-gDPP polymers were then fabricated. The devices feature a planar structure with channel dimensions of length *L* = 50 μm and width *W* = 1000 μm [42]. Before OECT characterization, we optimized the post-treatment temperature. As shown in Fig. S17, the transconductance reached its highest value at an annealing temperature of 200°C; therefore, 200°C was chosen as the optimal annealing temperature for the following research. The OECT performance of these polymers was characterized in an aqueous electrolyte (1 M NaCl). The transfer curves and output curves show that all three polymers with different molecular weights exhibited excellent ambipolar characteristics (Figs S18–S21). To quantitatively evaluate the OECT performance of these three polymers with varying molecular weights, we extracted their key figure-of-merit μC* by linearly fitting *g*_m_ with different channel volume (achieved by varying film thickness) (Fig. S22 and Table S2) [43,44].


(1) \begin{eqnarray*}
{{{g}}}_{\mathrm{m}}\,{\mathrm{ = }}\left( {\frac{{{{Wd}}}}{{{L}}}} \right){\mathrm{\mu }}{{\mathrm{C}}}^{\mathrm{*}}{\mathrm{|(}}{{{V}}}_{{\mathrm{th}}}{\mathrm{ - }}{{{V}}}_{{\mathrm{GS}}}{\mathrm{)|}}.
\end{eqnarray*}

Herein, *g*_m_ denotes transconductance, *W, d*, and *L* represent channel width, depth, and length, respectively, *μ* stands for charge carrier mobility, C* corresponds to volumetric capacitance of the film, *V*_th_ and *V*_GS_ are the threshold voltage and the gate voltage. For the as-cast OECTs, the maximum N/P-type *g*_m_ and μC* of the S-gDPP-based OECT were 11.59 ± 1.80/3.55 ± 0.43 mS and 151.2 ± 3.0/59.6 ± 0.6 F cm^−1^ V^−1^ s^−1^, respectively. As the molecular weight increased, both the N/P-type maximum *g*_m_ and μC* rose to 32.45 ± 2.31/18.02 ± 5.54 mS and 301.0 ± 5.7/185.1 ± 21.5 F cm^−1^ V^−1^ s^−1^ for H-gDPP-based OECTs, respectively (Fig. S22 and Table S3).

**Figure 2.**
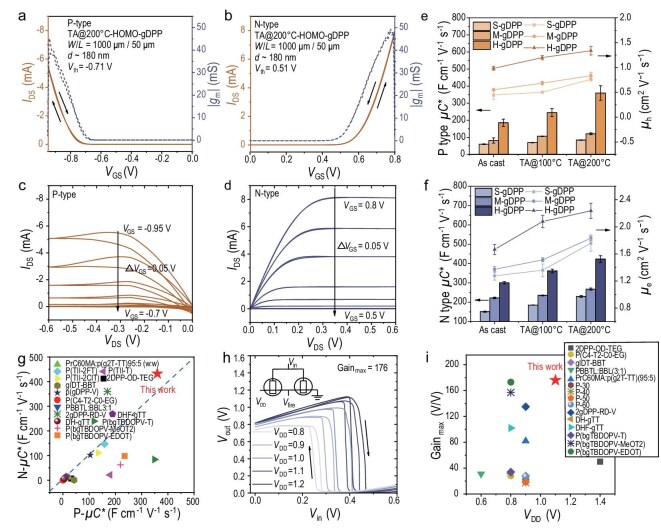
Characterizations of ambipolar OECTs and inverter. (a and b) P-type (a) and N-type (b) transfer characteristics curves of a 200°C annealed H-gDPP-based OECT (*V*_DS_ = ±0.4 V). (c and d) P-type (c) and N-type (d) output characteristics curves of a 200°C annealed H-gDPP-based OECT. (e) Statistical trends of P-type μC* and μ_h_ versus annealing temperature for three HOMO-gDPP polymers. Error bars for μC* and μ_h_ denote the fitting error. (f) Statistical trends of N-type μC* and μ_e_ versus annealing temperature for three polymers. Error bars for μC* and μ_e_ denote the fitting error. (g) μC* summary of reported ambipolar OMIECs. (h) Voltage transfer curves. (i) Gain values summary of the inverters based on ambipolar planar OECTs.

To further improve the molecular stacking and enhance the ambipolar charge transport behavior, we systematically performed thermal annealing (TA) on OECTs (Table 1 and Tables S3 and S4). Analysis of transfer and output characteristics (Fig. 2a–d, Figs S18–S21) demonstrated that all three polymers subjected to 200°C annealing exhibited superior ambipolar characteristics. Among these, H-gDPP-based OECTs achieved N/P-type maximum *g*_m_ of 43.0 ± 6.2/28.3 ± 9.4 mS and a high on/off ratio of 10^5^. The extracted μC* in N/P-type operation modes reached 423.3 ± 17.3/359.8 ± 42.6 F cm^−1^ V^−1^ s^−1^ (Fig. S22), which are 1.8 and 4.3 times higher than those of S-gDPP-based devices (Fig. 2e, f and Table 1). To decouple the influences of molecular weight optimization on charge transport and ion injection, we conducted an independent analysis of *μ* and C*. The *μ* values were measured using the pulsed gate current injection method (Figs S23–S28), and the C* (Fig. S29) values were obtained from electrochemical impedance spectroscopy (EIS) measurements [45,46]. As shown in Fig. 2e, f and Table 1, both electron mobility (*μ*_e_) and hole mobility (*μ*_h_) exhibit a trend of increase with varying molecular weights: *μ*_e_/*μ*_h_ rises from 1.28 ± 0.06/0.45 ± 0.09 cm^2^ V^−1^ s^−1^ for as-cast S-gDPP to 1.67 ± 0.08/0.98 ± 0.04 cm^2^ V^−1^ s^−1^ for as-cast H-gDPP. After thermal annealing, the *μ*_e/_*μ*_h_ further rise from 1.76 ± 0.12/0.76 ± 0.03 cm^2^ V^−1^ s^−1^ for S-gDPP to 2.24 ± 0.11/1.34 ± 0.09 cm^2^ V^−1^ s^−1^ for H-gDPP. As shown in Fig. S29 and Table 1, C* also exhibits a trend of increase with varying molecular weights. After thermal annealing at 200°C, the C* further rises from 120.82 ± 6.8/81.5 ± 5.7 F cm^−3^ for N/P-type S-gDPP to 170.72 ± 6.8/198.8 ± 17.7 F cm^−3^ for N/P-type H-gDPP. These results indicate that tuning the molecular weights of ambipolar OMIECs can simultaneously improve and balance their charge and ion transport properties. We compared the overall performance of H-gDPP and other ambipolar OMIECs. As shown in Fig. 2g, Fig. S30, and Table S5, H-gDPP exhibits outstanding performance among existing materials, achieving the record-high μC* values in both N-type and P-type operation modes to date. These findings confirm that molecular weight control combined with thermal annealing can cooperatively enhance and balance both charge and ion transport properties of ambipolar OMIECs.

**Table 1.** Device parameters of 200°C annealed S-gDPP, M-gDPP, and H-gDPP-based OECTs.

Polymer^a^	S-gDPP		M-gDPP		H-gDPP	
Polarity	N	P	N	P	N	P
*d* (nm)	130 ± 5		130 ± 6		180 ± 5	
*g*_m max_ (mS)	16.3 ± 2.9	5.5 ± 0.2	20.2 ± 2.1	8.3 ± 0.5	43.0 ± 6.2	28.3 ± 9.4
*V*_th_ (V)	0.52 ± 0.01	−0.72 ± 0.02	0.51 ± 0.01	−0.71 ± 0.01	0.51 ± 0.01	−0.71 ± 0.01
μC* (F cm^−1^ V^−1^ s^−1^)^b^	229.6 ± 4.4	83.9 ± 4.5	268.4 ± 5.5	120.8 ± 5.5	423.3 ± 17.3	359.8 ± 42.6
*μ* (cm^2^ V^−1^ s^−1^)^c^	1.76 ± 0.12	0.76 ± 0.03	1.83 ± 0.03	0.83 ± 0.07	2.24 ± 0.11	1.34 ± 0.09
C* (F cm^−3^)^d^	120.82 ± 6.84	81.50 ± 5.69	132.68 ± 16.43	111.85 ± 16.65	170.72 ± 6.83	198.78 ± 17.67
*τ*_on_ (ms)^e^	8.66 ± 1.40	11.48 ± 1.33	7.31 ± 0.60	10.08 ± 1.76	6.30 ± 1.37	7.29 ± 1.43
On/off ratio	10^4^	10^3^	10^5^	10^4^	10^5^	10^5^

^a^Channel dimension: *W* = 1000 μm, *L* = 50 μm. ^b^Calculated by linear fitting of *g*_m_ with different channel dimensions (Fig. S22). For each dimension, data from four devices were used for fitting. ^c^Measured by the pulsed gate current injection method. ^d^Measured by EIS. ^e^Calculated by single exponential fitting of the device transient response (*d* ≈ 130 nm/130 nm/180 nm for S-gDPP/M-gDPP/H-gDPP).

The ambipolar inverters are key components for homogeneous ANCs [10]. Leveraging the high and balanced ambipolar performance of H-gDPP-based OECTs, we successfully fabricated an ambipolar inverter (Fig. 2h, inset). By measuring its voltage transfer characteristics under different supply voltage (*V*_DD_) and input voltage (*V*_in_) scan rate, we calculated the gain value of the inverter. The results show that the H-gDPP-based inverter can achieve a maximum gain value of 176 V/V (Figs S31 and S32). As summarized in Fig. 2i and Table S6, this gain value represents one of the highest levels reported to date among inverters based on planar ambipolar OECTs. This record-breaking gain performance can be attributed to the optimized and balanced N-type and P-type performance of the H-gDPP-based OECT, an advantage that is expected to improve the performance of homogeneous ANCs.

**Figure 3.**
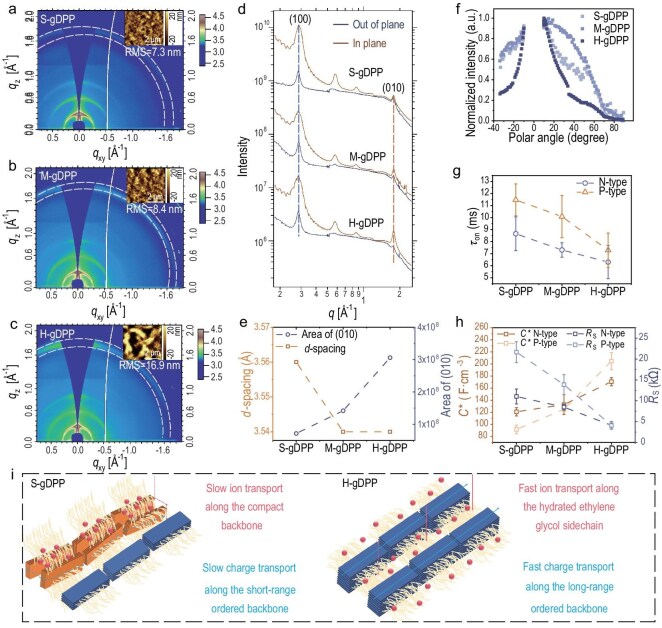
Effect of molecular weight on HOMO-gDPP microstructure and ion dynamics. (a–c) Two-dimensional GIWAXS patterns of three HOMO-gDPP polymer thin films with different molecular weights after 200°C annealing, with insets showing corresponding AFM images. (d) One-dimensional GIWAXS scattering profile along the in-plane and out-of-plane directions. (e) Evolution of (010) *d*-spacing and peak area with molecular weight. (f) Pole figure analysis of (010) peak. (g) OECT response time as a function of molecular weight. Error bars denote the s.d. for *n* = 4 devices, mean ± s.d. (h) Volumetric capacitance (C*) and ion transport resistance (*R*_S_) as functions of molecular weight. Error bars denote the s.d. for *n* = 4 devices, mean ± s.d.

We next investigated the key morphology factors affecting charge and ion transport. First, the influence of annealing temperature was studied. Grazing-incidence wide-angle X-ray scattering (GIWAXS) results (Fig. S33a and b) show that the crystallinity of H-gDPP films increased significantly after annealing at 200°C. In the 1D scattering profiles (Fig. S33c), the (010) π–π stacking peak in the out-of-plane direction (*q *= 1.76 Å^−1^) was analyzed by Gaussian fitting. The *d*-spacing decreased from 3.56 Å to 3.54 Å after annealing. Meanwhile, the peak area increased from 2.55 × 10^8^ to 3.06 × 10^8^ (Table S7). These results indicate that annealing promotes more ordered and compact π–π stacking, which is beneficial for charge transport along the polymer backbone. Based on these results, we fixed the annealing temperature at 200°C and further investigated the effect of molecular weight on film morphology and microstructure. Atomic force microscopy (AFM) characterization (Fig. 3a–c insets and Fig. S34) revealed significant surface morphological evolution with increasing molecular weight: S-gDPP films exhibited the smoothest surface (root mean square roughness, RMS = 7.3 nm), while M-gDPP (RMS = 8.4 nm) and H-gDPP (RMS = 16.9 nm) gradually presented a rough surface with coarse fibrous structures. This morphological transition toward fibrous structures indicates enhanced intermolecular aggregation in higher molecular weights, which promotes charge transport. To investigate the influence of molecular weight on crystallinity and molecular stacking orientation, we performed GIWAXS on HOMO-gDPP films with different molecular weights (Fig. 3a–c). One-dimensional scattering profiles (Fig. 3d) showed distinct (010) π-π stacking peaks at *q *= 1.76 Å^−1^ in the out-of-plane direction of the S-gDPP film. When the molecular weight increases, the location of the (010) peak gradually moves to high-*q* direction. Gaussian fitting analysis revealed that the *d*-spacing decreases from 3.56 Å for S-gDPP to 3.54 Å for H-gDPP. Meanwhile, as shown in Fig. 3e, the peak area significantly increases four times, from 7.17 × 10^7^ to 3.06 × 10^8^, confirming that increasing the molecular weight can lead to much more ordered and compact π–π stacking, which can greatly facilitate the charge transport along the polymer backbone.

Pole figure analysis of the (010) peak was then performed to show how the molecular weight influences the molecular orientation dependence (Fig. 3f). Interestingly, the face-on orientation proportion gradually increased from 69.3% for S-gDPP to 72.1% for M-gDPP and 85.5% for H-gDPP, indicating a nearly uniform face-on molecular orientation (Table S7). We analyze the possible reasons as follows: due to strong π-π interactions, high‑molecular‑weight H‑gDPP tends to form 2D lamellar supramolecular structures in chloroform [47]. During fast spin‑coating, its high viscosity restricts chain mobility, preventing rearrangement. Consequently, the pre‑formed 2D structure is locked into the solid film, resulting in high crystallinity and a predominant face‑on orientation. In contrast, low‑molecular‑weight polymers form 1D worm‑like structures in solution. Their low viscosity allows more time for rearrangement during film formation, leading to an edge‑on orientation. For planar OECTs, such a high face‑on orientation can significantly improve vertical ion transport in the channels, leading to enhanced volumetric capacitance and device response speed under repeated doping/dedoping cycles [48]. To verify this assumption, we measured the OECT response time and found that higher molecular weight leads to faster response (Fig. 3g and Fig. S35). Taking into account that the OECT is a kind of device whose response time is related to the *RC* constant, the higher face-on ratio leads to an increase in C* and a significant reduction in ion transport resistance. Here, we quantify the ion transport resistance using the following relationship [43]:


(2) \begin{eqnarray*}
{\mathrm{\tau = }}\,{{{R}}}_s{{C}}.
\end{eqnarray*}

Here, *τ* represents the OECT response time, *R*_s_ denotes the solution resistance (ion transport resistance), C = C^*^WdL denotes the channel capacitance. The calculating results are shown in Fig. 3h and Table 1. Specifically, the *R*_s_ of the N-type mode decreases from 11.0 kΩ (S-gDPP) to 4.1 kΩ (H-gDPP), while that of the P-type mode declines from 21.7 kΩ (S-gDPP) to 4.1 kΩ (H-gDPP). Further investigation revealed that the ion diffusion coefficient exhibited an increasing trend with increasing molecular weight (Figs S36 and S37), mirroring the evolutionary pattern observed in resistance. Specifically, the cation diffusion coefficient rise from 9.67 × 10^−9^ cm^2^ s^−1^ to 1.11 × 10^−6^ cm^2^ s^−1^, while the anion diffusion coefficient increased from 3.77 × 10^−9^ cm^2^ s^−1^ to 2.33 × 10^−7^ cm^2^ s^−1^.

The above results demonstrated that overall performance optimization stems from two synergistic effects of channel microstructure (illustrated in Fig. 3i): (1) Increasing molecular weight improves the crystallinity of OMIECs, which boosts hole and electron mobility. (2) The increase in face-on orientation ratio enhances ion transport and ion-charge coupling efficiency through the hydrated inter-side-chain pathways, which consequently improve C* and maintain fast device response. Combining these two effects, we successfully overcome the trade-off between the charge and ion transport in ambipolar OMIECs through the molecular weight optimization strategy, laying the foundation for homogeneous ANCs.

**Figure 4.**
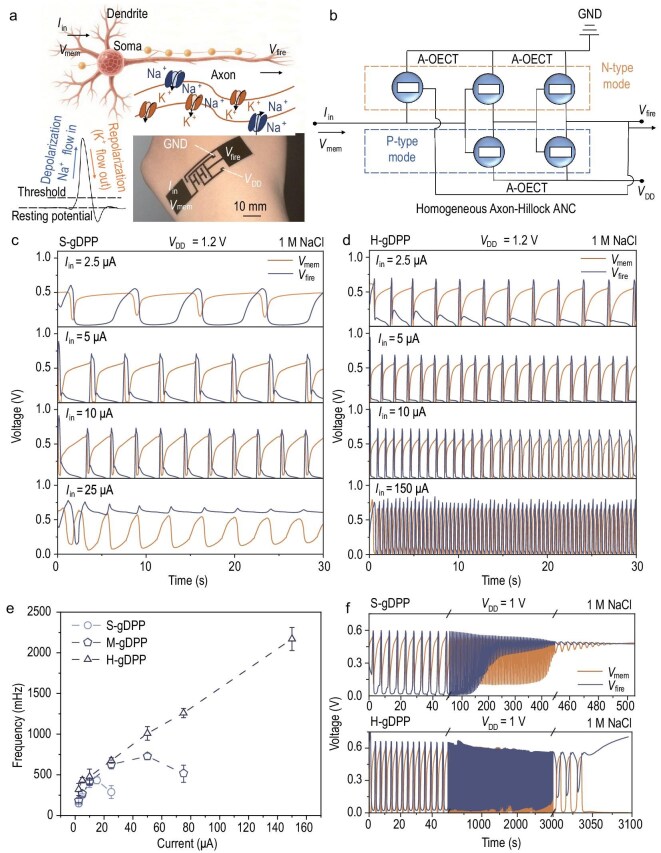
Homogeneous ANCs based on ambipolar OMIECs with different molecular weights and their biological analogy. (a) Schematic structure of a biological neuron and its working mechanism. Bottom panels show the different phases of a biological action potential and a photo of a flexible homogeneous ANC that mimics the biological neuron. (b) Circuit diagram of a homogeneous ANC based on the Axon Hillock circuit. (c and d) Spiking behavior of ANCs based on S-gDPP (c) and H-gDPP (d) with different input currents (*W* = 1000 μm, *L* = 50 μm, *d* ≈ 130 nm). (e) Plot of spiking frequency versus input current for ANCs based on HOMO-gDPP with different molecular weights. Error bars denote the s.d. for *n* = 4 ANCs, mean ± s.d. (f) Operation stability comparison of ANCs based on S-gDPP and H-gDPP.

We implemented homogeneous ANC using the Axon-Hillock (A-H) model to simulate the characteristics of biological neurons (Fig. 4a and b) [10,15]. The ANC design replicates key biological features: The rest state sustains a transmembrane potential via asymmetric ion distribution, creating an electrochemical driving force. Upon sufficient stimulation, voltage-gated Na^+^ channels open to initiate depolarization. Subsequently, K^+^ flows out through the activated channels, leading to repolarization. The distinct timing of these channels ensures unidirectional signal propagation and recovery to the resting state [14,49,50]. In our homogeneous ANC implementation, both the resetting transistor and amplifying block are uniformly fabricated with ambipolar OECTs (A-OECTs). The depolarization is achieved through constant current input (*I*_in _= 2.5–150 μA) charging the A-OECTs until the devices turn on in N-type mode and turn off in P-type mode, while repolarization is mediated by the discharge of resetting A-OECTs in N-type mode.

The key characteristic of ANCs is that the spiking frequency can be modulated by varying *I*_in_. As shown in Fig. 4c–e and Fig. S38, when *I*_in_ is low (<20 μA), we observed that all three polymers have similar spiking frequency, while higher currents (>20 μA) induce performance degradation in S-gDPP-based and M-gDPP ANCs. In contrast, the excellent linear correlation between spiking frequency and *I*_in_ was observed for H-gDPP-based ANCs. This can be attributed to the highly crystalline and entangled chain network in H-gDPP channels, which reduces defects and limits film swelling. This robust morphology helps the material resist side reactions and stress from doping, leading to an excellent linear correlation between spiking frequency and input current, and significantly extending the operational lifetime of H-gDPP-based ANCs [51,52]. Figure 4f and Fig. S39 show a clear molecular weight dependence. The H-gDPP-based ANC has better stability (lifetime > 3000 s). In contrast, the S-gDPP-based ANC decays quickly after 500 seconds. We found that this decay is mainly due to its poor P-type transistor stability—its transconductance drops sharply over time, so it cannot charge the membrane capacitor to produce pulses (Figs S40–S42). As a key advantage of OMIEC-based ANCs, the spiking threshold of *V*_mem_ can be readily modulated by ion concentration, similar to biological neurons. Notably, the H-gDPP-based ANC exhibits exceptional sensitivity, whose spiking behavior can be triggered by 0.04 M NaCl aq. (Fig. S43). This threshold is 10 times lower than that of S-gDPP-based ANC. Moreover, the five individual OECTs in the H-gDPP-based ANC all show good ambipolar characteristics with high device consistency (Figs S44–S46). This makes it feasible to fabricate high-density ANC arrays in future work for large-scale integration and spatial sensing.

**Figure 5.**
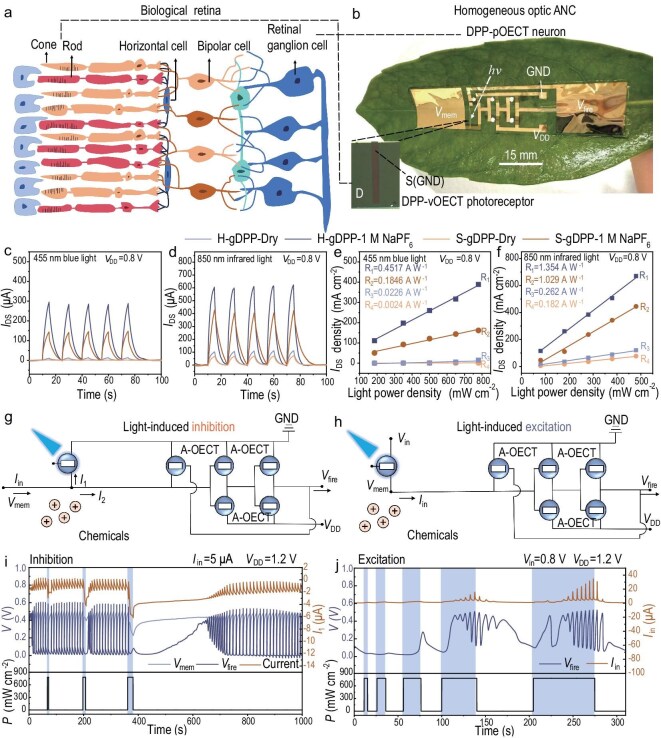
Homogeneous optic ANC with fused sensing-processing function. (a) Diagram illustrating the biological retinal pathway. (b) Photograph of a flexible optic ANC attached to a leaf. The inset shows a v-OECT photoreceptor. (c and d) Channel current response of H-gDPP and S-gDPP-based v-OECTs upon 455 nm (c) and 850 nm (d) light stimuli, tested in both dry film and 1 M NaPF_6_ environments (light intensity: 800 mW cm^−2^, *V*_DS_ = 0.8 V, gate is open circuit during the test). (e and f) Linear fit of channel current density versus light intensity under 455 nm (e) and 850 nm (f) light stimuli. The fitting *R*^2^ of *R*_1_, *R*_2_, *R*_3_, *R*_4_ in (e) are 0.99, 0.98, 0.72, 0.94; the fitting *R*^2^ of *R*_1_, *R*_2_, *R*_3_, *R*_4_ in (f) are 0.99, 0.99, 0.99, 0.99. (g and h) Circuit diagram of a homogeneous optic ANC based on a HOMO-gDPP-based v-OECT photoreceptor and Axon-Hillock neuron. The parallel circuit (g) and series circuit (h) can achieve light-induced inhibition and excitation functions, respectively. (i and j) The dynamic response of optic ANC upon light stimuli was tested in 1 M NaPF_6_ electrolyte under 455 nm light with an intensity of 780 mW/cm^2^. For (i), pulse width = 5/10/20 s, shaded areas indicate the light-stimulus periods, *V*_DD_ = 1.2 V, *I*_in_ = 5 μA. For (j), pulse width = 5/10/20/40/70 s, shaded areas indicate the light-stimulus periods, *V*_DD_ = 1.2 V, *V*_in_ = 0.8 V.

Visual signals contain over 90% of human input information. Building flexible and biological artificial optic nerves based on homogeneous ANC is crucial for fields like clinical vision restoration for the blind and machine vision. Besides being the core part of the neuron circuit, H-gDPP can also be used for photoreceptors, providing a material base for vision systems that combine sensing, memory, and processing. We first tested the photoresponse of planar OECT (p-OECT) and found it was weak (Fig. S47). To increase the photoresponse, we switched to a vertical OECT (v-OECT) structure. The photoresponse and electrical performance (Fig. S49) of the v-OECT in 1 M NaCl and 1 M NaPF₆ electrolytes were then tested (Figs S48 and S50). The results show that the v-OECT gives higher photoresponse and transconductance. Moreover, the photoresponse in NaPF₆ is about three times higher than in NaCl (Fig. S50). Based on these improvements, we used a single material, H-gDPP, to build both an artificial photoreceptor and a neuron, achieving a homogeneous artificial optic nerve that integrates sensing and processing. These two parts mimic the functions of cone/rod cells and ganglion cells in the retina, respectively (Fig. 5a and b). The neuron circuit uses a p-OECT, and the photoreceptor uses a high-sensitivity v-OECT [37,53]. To our knowledge, this is the first time that both the sensor and processing unit of ANC can be emulated by a single material.

We first evaluated the photoelectrical response characteristics of three HOMO-gDPP polymer-based v-OECTs under different light wavelengths, intensities, and chemical environments [26,54]. Similar to biological nerve, the photoresponse can be modulated by the surrounding chemical environment: introducing 1 M NaPF_6_ electrolyte significantly enhances photoresponse. More importantly, the photoresponse properties demonstrate a significant molecular weight dependence: under 455 nm blue light illumination, the light sensitivity (*R*) of S-gDPP-based v-OECT is 0.18 A W^−1^. In comparison, H-gDPP attains 0.45 A W^−1^ (Fig. 5c and e, and Fig. S50). The lights with two wavelengths (455 nm and 850 nm) were selected because they correspond to the two main absorption peaks of H-gDPP (Fig. 1d). Leveraging the narrow energy band gap of HOMO-gDPP, the photoreceptor can also be activated by near-infrared light that can penetrate the skin, making our ANCs applicable for *in vivo* applications. As shown in Fig. 5d and f, and Fig. S50, 850 nm infrared light irradiation can result in a light sensitivity of 1.03 A W^−1^ for S-gDPP-based v-OECT, while that of H-gDPP achieves 1.35 A W^−1^. These results indicate that increasing the molecular weight can effectively enhance the photosensitivity of HOMO-gDPP, which can be attributed to the microstructure-induced optimized ionic-charge coupling and charge transport in H-gDPP polymer [26].

As we have demonstrated the effectiveness of the molecular weight optimization strategy on in optimizing the performance of artificial neuron and photoreceptor, these two parts were next integrated onto an ultra-flexible parylene substrate to construct homogeneous optic ANCs. As shown in Fig. 5g and h and Fig. S51, our ANCs were designed to emulate the vision functions of the biological retina in different environments: (1) In the bright light environment, the action potential of neurons will be inhibited, thereby protecting the retina from impairment. This can be achieved through the parallel connection of receptor and neuron (Fig. 5g) with the following mechanism: the input current (*I*_2_) of the neuron depends on the resistance ratio of the v-OECT channel and neuron circuit, which will decrease when exposed to bright light and lead to neuron inhibition. (2) In the dark environment, the action potential of neurons will be generated directly by the light stimuli, which can be achieved through the series connection of receptor and neuron (Fig. 5h). As shown in Fig. 5i and j, 455 nm, 780 mW cm^−2^ light pulse stimuli with gradually increased pulse width can dynamically modulate the spiking frequency of ANC from ~140 mHz to 0 Hz for inhibition and 0 to ~230 mHz for excitation. After that, the spiking frequency can recover after the light stimuli. We also note that by introducing an ionic environment, the light-modulated effect can be more obvious than that in the dry state, evidenced by the fact that the light stimuli can more easily trigger the inhibition/excitation behavior (Fig. S52), which successfully emulates the chemical-modulated vision characteristics of the retina [55].

Finally, we also evaluated the potential of flexible homogeneous ANC for future implantable/wearable applications. First, in PC-12 cell culture tests, we found that the H-gDPP films exhibit much higher cell density and MTT activity than the S-gDPP films (Fig. S53), indicating that the high-molecular-weight material is more biocompatible. We deduce that the high face-on ratio of H-gDPP may be more suitable for cell growth, enabling long-term safe adhesion to tissues and the skin surface. Second, the ultrathin parylene substrate allows the ANC to be arbitrarily bent while maintaining stable operation (Fig. S54 and Video S1), enabling the ANC to adapt well to complex shape changes of tissues and organs and achieve excellent conformability.

## CONCLUSIONS

In conclusion, this work establishes molecular weight control as a powerful and effective strategy for overcoming the long-standing electronic-ionic transport trade-off in ambipolar OMIECs. By tuning the molecular weight of the HOMO-gDPP polymer, we achieved a synergistic optimization of the thin-film microstructure. This resulted in a highly ordered, face-on-dominated molecular orientation, which simultaneously enhanced hole/electron transport and ion transport. These synergistic improvements culminated in record-breaking OECT performance, with μC* values reaching 423.3 F cm^−1^ V^−1^ s^−1^ in N-type mode and 359.8 F cm^−1^ V^−1^ s^−1^ in P-type mode, the highest reported for ambipolar OMIECs to date. This material breakthrough directly enabled the demonstration of the first flexible, fully homogeneous ANC. H-gDPP simultaneously achieves a stable ANC and a highly sensitive photoreceptor, highlighting its promise for constructing homogeneous and multifunctional neuromorphic circuits. The monolithic integration of photoreceptors and spiking neurons using a single OMIEC material eliminated the performance and mechanical mismatches inherent in previous heterogeneous systems. The resulting optic ANC successfully emulated critical biological functions, including chemical-modulated light-inhibitory/excitatory behaviors, with an improvement in spiking frequency range, light sensitivity and operational stability. This research establishes a definitive pathway for designing high-performance ambipolar OMIECs, opens new avenues for sophisticated bio-interfacing systems in clinical nerve repair, and paves the way for next-generation, low-power adaptive machine vision.

## METHODS

All detailed experimental procedures and characterization methods are provided in the Supplementary data.

### Materials

Three HOMO-gDPP polymers with different molecular weights were synthesized as described in Note S1. All reagents were purchased from Sigma-Aldrich and used without further purification. Polymer solutions were prepared in chloroform and stirred overnight at 50°C before use.

### OECT and ANC fabrication

Planar OECTs were fabricated on glass substrates cleaned by ultrasonication and UV–ozone treatment. Cr/Au electrodes were deposited by thermal evaporation through shadow masks, defining channels of *W* = 1000 μm and *L* = 50 μm. HOMO-gDPP films were spin-coated and thermally annealed, yielding thicknesses of 40–180 nm measured using a stylus profiler. Flexible ANCs were fabricated on ~10 μm parylene substrates following similar procedures, with Ag ink used as the gate electrode. Detailed fabrication parameters are provided in the Supplementary data.

### Electrical characterization of OECTs, photoreceptor and optic ANCs

Electrical measurements were performed using Keithley 2602B source meters controlled by Kickstart software. OECTs were characterized in 1 M NaCl electrolyte with Ag/AgCl gate electrodes. Optical responses were investigated under 455 and 850 nm LED illumination. Detailed testing conditions are described in the Supplementary data.

### UV-Vis-NIR absorption spectroscopy measurements

UV-vis-NIR absorption spectra were collected using a Shimadzu UV-3600 Plus spectrophotometer.

### Spectroelectrochemical characterization

Spectroelectrochemical measurements were conducted on polymer-coated ITO substrates in 1 M NaCl electrolyte with applied potentials controlled by a sourcemeter.

### Atomic force microscopy (AFM)

AFM measurements were performed in tapping mode using a Veeco INNOVA atomic force microscope on films prepared on glass substrates; scan size 5 × 5 μm².

### Grazing-incidence wide-angle X-ray Scattering (GIWAXS)

GIWAXS measurements were performed at beamline 7.3.3 of the Advanced Light Source using polymer films spin-coated on Au-coated silicon substrates [56]. Scattering data were collected with a Dectris Pilatus 2 M detector and analyzed using Igor Pro with the Nika package [57].

### Cyclic voltammetry (CV)

CV measurements were performed using an Autolab PGSTAT302N workstation with a standard three-electrode configuration. Ferrocene was used as the internal standard for extracting electron affinity (EA) and ionization potential (IP). Scan-rate-dependent CV data were analyzed using the Randles–Ševčík equation [58–60].


\begin{eqnarray*}
{I}_{\mathrm{P}}\,{\mathrm{ = 0}}{\mathrm{.4463}}{nFAC}{{\mathrm{\Big(}}\frac{{{nFvD}}}{{RT}}{\mathrm{\Big)}}}^{{\mathrm{1/2}}},
\end{eqnarray*}

where *I*_p_ denotes the peak current, *n* the electron transfer number per reaction (here *n* = 1), *F* the Faraday constant, *A* the working electrode area, *C* the reactant molar concentration, *ν* the voltage scan rate, *D* the ion diffusion coefficient, *R* the gas constant, and *T* the temperature.

### Pulsed gate current injection technique and μ extraction

For planar OECTs, mobility (*μ*) was extracted from the transient drain current response under pulsed gate current injection [45,61].

### Electrochemical impedance spectroscopy (EIS)

EIS measurements were performed using an Autolab PGSTAT302N workstation in 1 M NaCl electrolyte, and volumetric capacitance (C*) was extracted by fitting the impedance spectra.

### Biocompatibility

PC-12 cells were cultured on polymer-coated and control substrates under standard conditions. Cell viability was evaluated after 1, 3 and 5 days using MTT assays and LIVE/DEAD fluorescence staining. Detailed experimental procedures are provided in the Supplementary data.

## Supplementary Material

nwag341_Supplementary_data_r
