# Supplementary material for: Face-on crystallization in ambipolar organic mixed conductors enables homogeneous light-modulated artificial neurons
Source: Natl Sci Rev. 2026 Jun 9;13(14):nwag341. doi: 10.1093/nsr/nwag341 (PMC13382900; doi:10.1093/nsr/nwag341)
Supplement: nwag341_Supplementary_data_r [file nwag341_supplementary_data_r.pdf]

RESEARCH ARTICLE

MATERIALS SCIENCE

Face-on crystallization in ambipolar organic mixed conductors enables homogeneous light-modulated artificial neurons

Yulin Zhang[1,†], Shijie Wang[1,†,*], Guangyu Qi[1,†], Xinmei Cai[1], Sen Zhang[1], Bingjun Wang[1], Chao Zhao[1], Yong Han[1], Qunping Fan[1], Laili Wang[2,*] and Wei Ma[1,*]

[1]State Key Laboratory for Mechanical Behavior of Materials, Xi'an Jiaotong University, Xi'an 710049, China;

[2]State Key Laboratory of Electrical Insulation and Power Equipment, Xi'an Jiaotong University, Xi'an 710049, China

***Corresponding authors.** E-mails: msewsj@xjtu.edu.cn, llwang@mail.xjtu.edu.cn, msewma@xjtu.edu.cn

[†]Equally contributed to this work.

METHODS

Materials

The detailed synthesis route of three HOMO-gDPP polymers with different molecular weights can be found in **Note S1**. All reagents were purchased from Sigma-Aldrich without further purification. The HOMO-gDPP polymers were dissolved in chloroform and stirred overnight at 50°C before use.

OECT and ANC fabrication.

For planar OECT fabrication, glass substrates were sequentially cleaned via ultrasonication in acetone and isopropanol (99.5%, Sigma-Aldrich) (30 min each) and subsequently treated with UV-ozone for 15 min. The substrates were then loaded into a thermal evaporator (Angstrom NEXDEP PVD platform), 5 nm Cr adhesion layer (0.2 Å s^{-1}) and a 50 nm Au electrode layer (1 Å s^{-1}) were sequentially deposited. Channel dimensions (width W = 1000 μm, length L = 50 μm) were defined using a high-resolution shadow mask. Finally, HOMO-gDPP thin films were spin-coated with different spin speeds in a nitrogen glovebox and subjected to thermal annealing (TA) as required. The channel thickness ranges from 40 to 180 nm. To measure the thickness, we used a stylus profiler (Dektak XT, Bruker). After device fabrication, a step was scratched at the channel edge with a blade, and four points within a 5 mm region along the scratched step were measured. The average of these four points was taken as the channel thickness of that device (**Table S2**). For the fabrication of flexible ANC, cleaned parylene substrates (parylene thickness: ~10 μm) underwent the same cleaning and UV-ozone procedure. During the thermal evaporation, spin-coating and thermal annealing, the parylene film was temporarily attached to a glass slide as a support substrate. After these procedures, the ANC can be peeled off from the glass to obtain a free-standing ultra-flexible ANC. Circuit patterns were defined using a high-resolution shadow mask. The HOMO-gDPP was spin-coated in the glovebox, followed by 2 hours of TA treatment. Finally, Ag ink (ELECTROLUBE, Ag content 50-85%) was drop-coated to form the gate electrodes.

Electrical characterization of OECTs

For OECT device characterization, 1 M NaCl aqueous solution served as the electrolyte, with an Ag/AgCl pellet (Warner Instruments E205) acting as the gate electrode. The measurements were conducted using a Keithley 2602B SMU connected to the three electrodes of each device via an M4 probe station (Shenzhen Uotest Electronic Technology Co., Ltd). The Kickstart software was employed to program the desired drain and gate voltage profiles while simultaneously recording the corresponding drain and gate currents. For transfer curve measurements, a constant drain voltage (V_{DS}) of ±0.4 V was applied.

Electrical characterization of photoreceptor and optic ANCs.

The photo-response of the v-OECT was investigated under irradiation from monochromatic light-emitting diode (LED-Thorlabs DC2200) sources with wavelengths of 455 nm and 850 nm. The light intensity for both LEDs was calibrated to specific values within the range of 150–800 mW·cm^{-2} using a certified optical power meter (Thorlabs PM100D). The electrical characteristics of the ANCs were measured with two Keithley 2602B SMUs using four channels. The first channel supplied a V_{DD} in the range of 1–1.2 V. While the second channel provided an input current (I_{in}) ranging from 2.5 to 150 μA and monitored the membrane potential (V_{mem}). The third channel was used to monitor the dynamic response of the output voltage (V_{fire}) of ANCs. The fourth channel is grounded and monitors the drain current of the v-OECT (I_1). The software Kickstart v2.4 was used to control the SMU.

UV-Vis-NIR absorption spectroscopy measurements

UV-vis-NIR absorption spectra were acquired using a Shimadzu UV-3600 Plus spectrophotometer. Solution-phase measurements were performed on HOMO-gDPP CF solutions contained in quartz cuvettes. For thin-film characterization, samples were prepared by spin-coating (2000 rpm) 20 mg·mL^{-1} CF solutions onto clean glass substrates, yielding uniform films with thicknesses of approximately 85 nm. All samples subsequently underwent thermal annealing (TA) at 200°C for 2 h in a nitrogen-

atmosphere glovebox. Consistent spin-coating and TA protocols were maintained for all experiments.

Spectroelectrochemical characterization

Spectroelectrochemical measurements employed ITO-coated glass substrates (Shenzhen Huanan Xiangcheng Co., Ltd.). The experimental setup consisted of the HOMO-gDPP sample immersed in 1 M NaCl aqueous electrolyte with an Ag/AgCl working electrode. Potentials were applied on Ag/AgCl and controlled using a Keithley 2602B sourcemeter operated via Kickstart software. Optical transmission (T) data were converted to absorbance (A) using the relationship $A = -\log_{10}T$.

Atomic force microscopy (AFM)

The AFM samples were fabricated on clean glass substrates using a method similar to device fabrication. The AFM characterization was performed in tapping mode using a Veeco INNOVA atomic force microscope, with the x and y axes of the AFM images scaled at 5×5 μm^2 and a scale bar of 1 μm.

Grazing-incidence wide-angle X-ray scattering (GIWAXS)

GIWAXS measurements were performed at beamline 7.3.3 at the Advanced Light Source (ALS).[1] The samples were prepared by spin-coating onto gold-coated silicon substrates. For dry film samples, no additional processing steps were required. During characterization, a 10 keV X-ray beam was incident on the samples at an incident angle of 0.14° for measurement in vacuum. Then an optimal angle was selected to maximize the scattering intensity. The scattered X-ray photons were detected using a two-dimensional Dectris Pilatus 2M detector. Data analysis was completed using Igor Pro software with the Nika package.[2]

Cyclic voltammetry (CV)

Electrochemical analysis was performed on an Autolab PGSTAT302N electrochemical workstation with a conventional three-electrode configuration. The reference, counter,

and working electrodes were an Ag/AgCl, a platinum sheet, and a polymer-coated ITO substrate, respectively. The working electrode was fabricated by spin-coating S-gDPP, M-gDPP, and H-gDPP, chloroform solutions onto pre-cleaned ITO substrates. The electrolyte was 0.1 M tetrabutylammonium hexafluorophosphate acetonitrile solution, and ferrocene (Fc) was added as the internal standard. The voltage scan range was −0.8 to 0.8 V. The electron affinity (EA) and ionization potential (IP) were extracted by:

$$\mathrm{EA}=q(\varphi_{\mathrm{red}}-\varphi_{\mathrm{ox,\,Fc}})+4.8 \qquad (\mathrm{eV})$$

$$\mathrm{IP}=q(\varphi_{\mathrm{ox}}-\varphi_{\mathrm{ox,\,Fc}})+4.8 \qquad (\mathrm{eV})$$

where q is the elementary charge, φ_{red} (φ_{ox}) the reduction (oxidation) onset potential of HOMO-gDPP and $\varphi_{\mathrm{ox,\,Fc}}$ the half-wave potential of the ferrocene/ferrocenium (Fc/Fc+) redox couple. For scan-rate-dependent CV tests, the electrolyte was 0.1 M NaCl aqueous solution, and data were analyzed according to the Randles–Ševčík equation:[3–5]

$$I_{\mathrm{P}}=0.4463nFAC\left(\frac{nFvD}{RT}\right)^{1/2}$$

where I_{p} denotes the peak current, n the electron transfer number per reaction (here n = 1), F the Faraday constant, A the working electrode area, C the reactant molar concentration, v the voltage scan rate, D the ion diffusion coefficient, R the gas constant, and T the temperature.

Pulsed gate current injection technique and μ extraction.

For planar OECTs, the mobility μ was extracted by applying gate current (I_{GS}) pulses to the device and recording the drain current (I_{DS}) response over time (t). From the linearly increasing I_{DS}, the slope $\partial I_{\mathrm{DS}}/\partial t$ was calculated, and μ was derived by:[6,7]

$$\mu = \frac{\partial I_{DS}}{\partial t} \cdot \frac{L^2}{I_{GS} V_{DS}}$$

where V_{DS} is the drain voltage (±0.4 V) and L is the channel length.

Electrochemical impedance spectroscopy (EIS)

EIS measurements were performed using an Autolab PGSTAT302N workstation. The three electrodes were the same as for CV measurements, except that the substrate on which the polymer film was deposited was changed to Au-coated glass. The electrolyte was 1 M NaCl. A small sinusoidal AC voltage of 50 mV amplitude with varying frequency (10^5 to 10^{-1} Hz) was applied to the sample, superimposed on a DC bias potential of −0.8/0.9 V for N/P-type. The measured frequency-dependent impedance Z was analyzed using NOVA 2.1 software, and the simplified Randles circuit model was chosen, consisting of a series resistor R_S, a parallel resistor R_P, and a parallel capacitor C. The C^* values were calculated by dividing the fitted C by the film volume.

Biocompatibility

PC-12 cells (BeNa Culture Collection, 100235) were cultured with MEM-α supplemented with 10% fetal bovine serum (FBS, Gibco, USA) and 1% penicillin-streptomycin (Gibco, USA). The cells were incubated in a humidified atmosphere incubator with 5% CO_2 at 37 °C, and the culture medium was refreshed every 2 days throughout the incubation period. The S-gDPP/glass, H-gDPP/glass, and bare glass samples were sterilized and placed centrally in 12-well plates, and PC-12 cells were seeded on each sample at a density of 10^4 cells/mL. Afterward, the cell-adhered samples were incubated for 1 and 3 days. In this experiment, the cells (10^4 cells/mL) cultured on bare glass samples were used as a control.

At each time point, the cells-adhered samples were rinsed twice with PBS and transferred into new 12-well TCP. 500 μL 3-(4,5-Dimethylthiazol-2-yl)-2,5-diphenyltetrazolium bromide (MTT) solution (5 mg/mL MTT in PBS) was added to each well with continuous culture at 37 °C for 4 h. After removal of the solution, 500 μL DMSO was added to each well and oscillated for 10 min. Finally, 100 μL of the

resultant solution was pipetted from each well and transferred to a new 96-well TCP, and the absorbance was measured at 470 nm using the Multiscan GO microreader. Four replicates from each group were used at each time point.

Live/dead staining using the LIVE/DEAD Viability/Cytotoxicity Kit (Molecular Probes, Invitrogen, France) was performed to identify viable and nonviable cells on the samples. At the end of each time period, the cell-adhered samples were washed three times using PBS followed by the addition of 500 μL of PBS containing ethidium-homodimer-1 (4 μM) and calcein-AM (2 μM) to each well prior to incubation at 37 °C for 25 min. The fluorescence-stained cells were analyzed using the laser confocal microscope for the collection of images.

Note S1. Materials Synthesis

Compound 2,5,8,11,14-pentaoxahexadecan-16-ol, 3,6-di(thiophen-2-yl)-2,5-dihydropyrrolo[3,4-c]pyrrole-1,4-dione and 1,1,1,2,2,2-hexabutyldistannane were purchased and used directly without additional purification. HOMO-gDPP polymers were synthesized according to the following procedures.

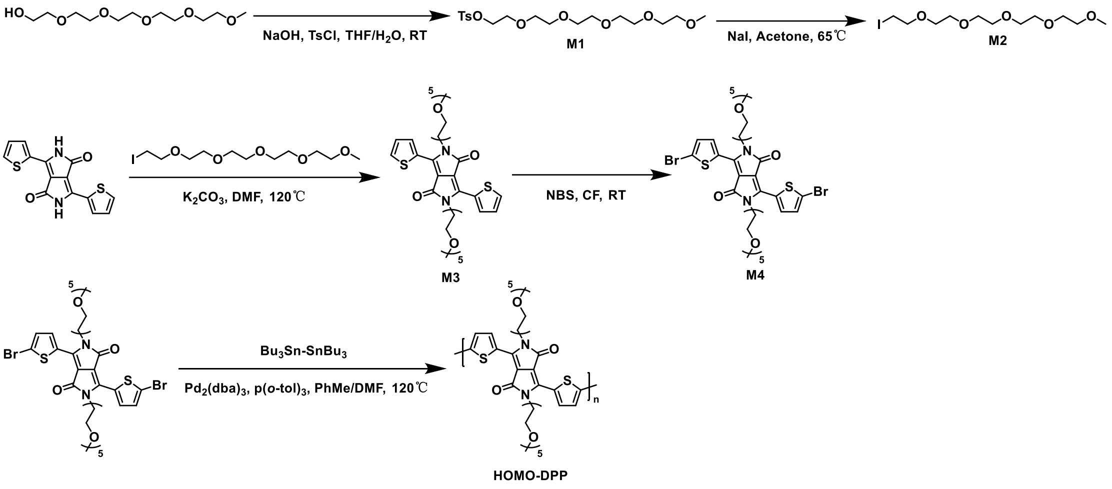

Figure S1. Synthesis routes of HOMO-gDPP

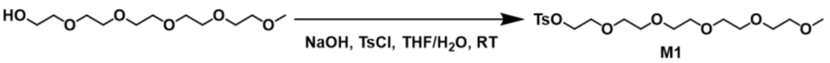

Compound **M1:** 2,5,8,11,14-pentaoxahexadecan-16-ol (10.0 g, 37.55 mmol, 1.0 equiv) and tetrahydrofuran (THF, 14 mL) were mixed in a dry 250 mL flask under N_2 atmosphere. Then, 3.6 M sodium hydroxide (NaOH) aqueous solution (31.3 mL, 112.64 mmol, 3.0 equiv) was added at 0 °C. After that, *p*-toluenesulfonyl chloride (TsCl, 9.31 g, 48.81 mmol, 1.3 equiv) was dissolved in THF (18 mL) and added to the mixture slowly. The mixture was stirred at room temperature for 12 h. After that, the mixture was poured into water and extracted with dichloromethane (DCM). The organic phase was dried over anhydrous sodium sulfate (Na_2SO_4) and the organic solvent was removed under vacuum. The product was obtained without further purification for direct use (15.0 g, yield 98.3 %, colorless liquid). ^{1}H NMR (400 MHz, $CDCl_3$) (ppm): δ 7.77 (d, J = 8.3 Hz, 2H), 7.32 (d, J = 8.4 Hz, 2H), 4.13 (t, J = 4.0 Hz, 2H), 3.66 (t, J = 4.0 Hz, 2H), 3.64 – 3.58 (m, 10H), 3.56 (s, 4H), 3.52 (dd, J = 5.6, 3.6 Hz, 2H), 3.35 (s, 3H), 2.42 (s, 3H).

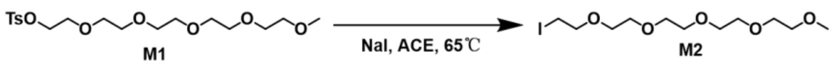

Compound **M2: M1** (15.0 g, 35.63 mmol, 1.0 equiv) and Sodium monoiodide (NaI, 7.96 g, 53.44 mmol, 1.5 equiv) were mixed in a dry 100 mL flask under N_2 atmosphere. Then, 40 mL of acetone was added. After that, the mixture was stirred at 56 °C overnight. After that, the mixture was poured into water and extracted with dichloromethane (DCM). Organic phase was dried over anhydrous sodium sulfate (Na_2SO_4) and organic solvent was remove under vacuum. The product was purified by silica gel column chromatography with eluent of ethyl acetate (EA): petroleum ether (PE) (1:1, v/v) to give compound **M2** (11.5 g, yield 89.3 %, yellow oil liquid). ^{1}H NMR (400 MHz, $CDCl_3$) (ppm): δ 3.74 (t, J = 8.0 Hz, 2H), 3.67 – 3.61 (m, 14H), 3.57 – 3.50 (m, 2H), 3.36 (s, 3H), 3.24 (t, J = 8.0 Hz, 2H).

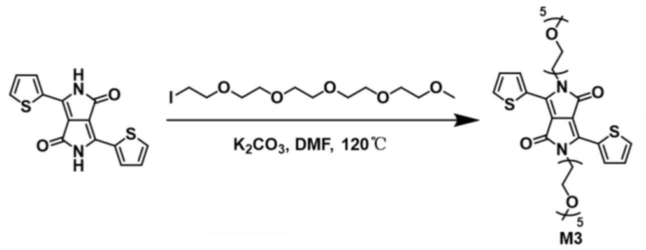

Compound **M3**: 3,6-di(thiophen-2-yl)-2,5-dihydropyrrolo[3,4-c]pyrrole-1,4-dione (0.98 g, 3.27 mmol, 1.0 equiv), **M2** (4.13 g, 9.80 mmol, 3.0 equiv), K_2CO_3 (4.52 g, 32.7 mmol, 10.0 equiv) and *N*, *N*-dimethylformamide (DMF, 16 mL) were mixed in a dry 50 mL flask under N_2 atmosphere, and then stirred at 120 °C for 12 h. The reaction was cooled down to room temperature and the mixture was poured into water and extracted with dichloromethane (DCM). The combined extracts were dried over anhydrous Na_2SO_4, and then the solvent was removed under vacuum. The residue was purified by silica gel column chromatography with eluent of ethyl acetate (EA) to give compound **M3** (1.12 g, yield 44.6 %, purple-black solid). ^{1}H NMR (400 MHz, $CDCl_3$) (ppm): δ 8.70 (dd, J = 4.0, 1.2 Hz, 2H), 7.60 (dd, J = 5.1, 1.2 Hz, 2H), 7.21 (dd, J = 5.0, 3.9 Hz, 2H), 4.22 (t, J = 6.3 Hz, 4H), 3.73 (t, J = 6.3 Hz, 4H), 3.61 (t, J = 2.5 Hz, 4H), 3.59 – 3.57 (m, 12H), 3.55 – 3.52 (m, 12H), 3.50 – 3.47 (m, 4H), 3.32 (s, 6H).

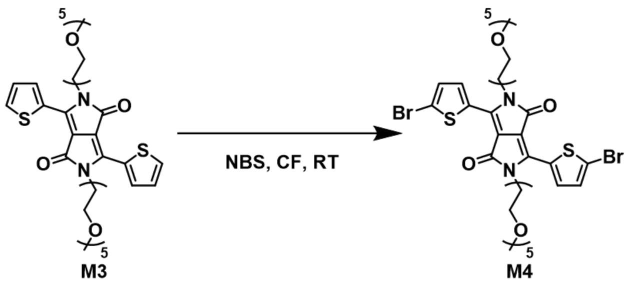

Compound **M4**: M3 (0.5 g, 0.65 mmol, 1.0 equiv) and chloroform ($CHCl_3$, 20 mL) were mixed in a dry 50 mL flask under N_2 atmosphere. N-Bromosuccinimide (NBS, 0.25 g, 1.43 mmol, 2.2 equiv) was added slowly at 0 °C and stirred at 0 °C for 30 min. Then, the mixture was transferred to room temperature and stirred overnight. After reaction was completed, the mixture was poured into the $Na_2S_2O_4$ solution and extracted with dichloromethane (DCM). The combined extracts were dried over anhydrous Na_2SO_4, and then the solvent was removed under vacuum. The residue was purified by silica gel column chromatography with eluent of ethyl acetate (EA) to give compound **M4** (0.23 g, yield 38.2 %, purple-black solid). ^1H NMR (400 MHz, $CDCl_3$) (ppm): δ 8.49 (d, J = 4.2 Hz, 2H), 7.21 (d, J = 4.2 Hz, 2H), 4.17 (t, J = 6.0 Hz, 4H), 3.77 (t, J = 6.0 Hz, 4H), 3.68 – 3.61 (m, 16H), 3.61 – 3.56 (m, 12H), 3.55 – 3.52 (m, 4H), 3.37 (s, 6H). ^{13}C NMR (100 MHz, $CDCl_3$) (ppm): δ 161.29, 139.51, 134.87, 131.43, 131.11, 119.37, 107.98, 71.93, 70.76, 70.62, 70.58, 70.55, 70.51, 69.69, 68.95, 59.04, 42.24. MALDI-TOF-MS (m/z): calculated: $C_{36}H_{50}Br_2O_{12}S_2$, $[M+H]^+$, 925.12, found 925.12.

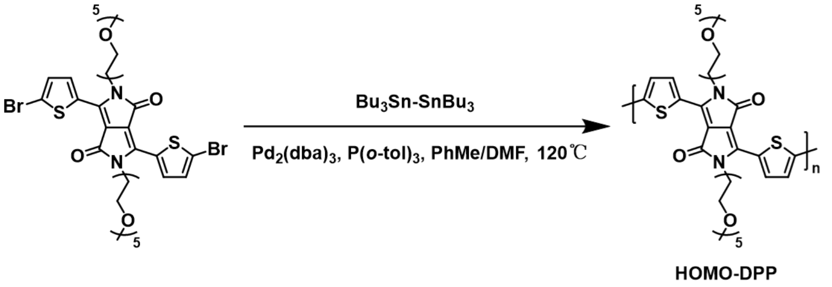

S-gDPP: Compound **M4** (70 mg, 0.076 mmol, 1.0 equiv), $Pd_2(dba)_3$ (1.79 mg, 0.002 mmol, 0.026 equiv) and P(*o*-tol)$_3$ (4.59 mg, 0.015 mmol, 0.2 equiv) were added in a dry 25 mL flask mixed under N_2 atmosphere, then the 1,1,1,2,2,2-hexabutyl-distannane (43.82 mg, 0.076 mmol, 1.0 equiv), toluene (1.5 mL) and *N*, *N*-dimethylformamide (1.5 mL) was added and the mixture was stirred at 120 °C for 30 min. After cooling to room

temperature, the polymer was precipitated into around 30 mL of methanol, and filtered. The polymer was extracted using Soxhlet apparatus with hexane, acetone, methanol, ethyl acetate and dichloromethane in order. The dichloromethane fraction was concentrated and precipitated into methanol and filtered to obtain S-gDPP (36 mg, yield 61.6 %, black solid). GPC (Hexafluoroisopropanol): M_n = 19.4 kDa and $Đ$ =1.6.

M-gDPP: Compound **M4** (70 mg, 0.076 mmol, 1.0 equiv), $Pd_2(dba)_3$ (1.79 mg, 0.002 mmol, 0.026 equiv) and P(*o*-tol)$_3$ (4.59 mg, 0.015 mmol, 0.2 equiv) were added in a dry 25 mL flask mixed under N_2 atmosphere, then the 1,1,1,2,2,2-hexabutyl-distannane (43.82 mg, 0.076 mmol, 1.0 equiv), toluene (1.5 mL) and *N*, *N*-dimethylformamide (1.5 mL) was added and the mixture was stirred at 120 °C for 7 hours. After cooling to room temperature, the polymer was precipitated into around 30 mL of methanol, and filtered. The polymer was extracted using Soxhlet apparatus with hexane, acetone, methanol, ethyl acetate and dichloromethane in order. The dichloromethane fraction was concentrated and precipitated into methanol and filtered to obtain M-gDPP (47 mg, yield 80.8 %, black solid). GPC (Hexafluoroisopropanol): M_n = 33.5 kDa and $Đ$ = 1.3.

H-gDPP: Compound **M4** (70 mg, 0.076 mmol, 1.0 equiv), $Pd_2(dba)_3$ (1.79 mg, 0.002 mmol, 0.026 equiv) and P(*o*-tol)$_3$ (4.59 mg, 0.015 mmol, 0.2 equiv) were added in a dry 25 mL flask mixed under N_2 atmosphere, then the 1,1,1,2,2,2-hexabutyl-distannane (43.82 mg, 0.076 mmol, 1.0 equiv), toluene (1.5 mL) and *N*, *N*-dimethylformamide (1.5 mL) was added and the mixture was stirred at 120 °C for 10.5 hours. After cooling to room temperature, the polymer was precipitated into around 30 mL of methanol, and filtered. The polymer was extracted using a Soxhlet apparatus with hexane, acetone, methanol, ethyl acetate and dichloromethane in order. The dichloromethane fraction was concentrated and precipitated into methanol and filtered to obtain H-gDPP (41 mg, yield 70.8 %, black solid). GPC (Hexafluoroisopropanol): M_n = 56.0 kDa and $Đ$ = 1.1.

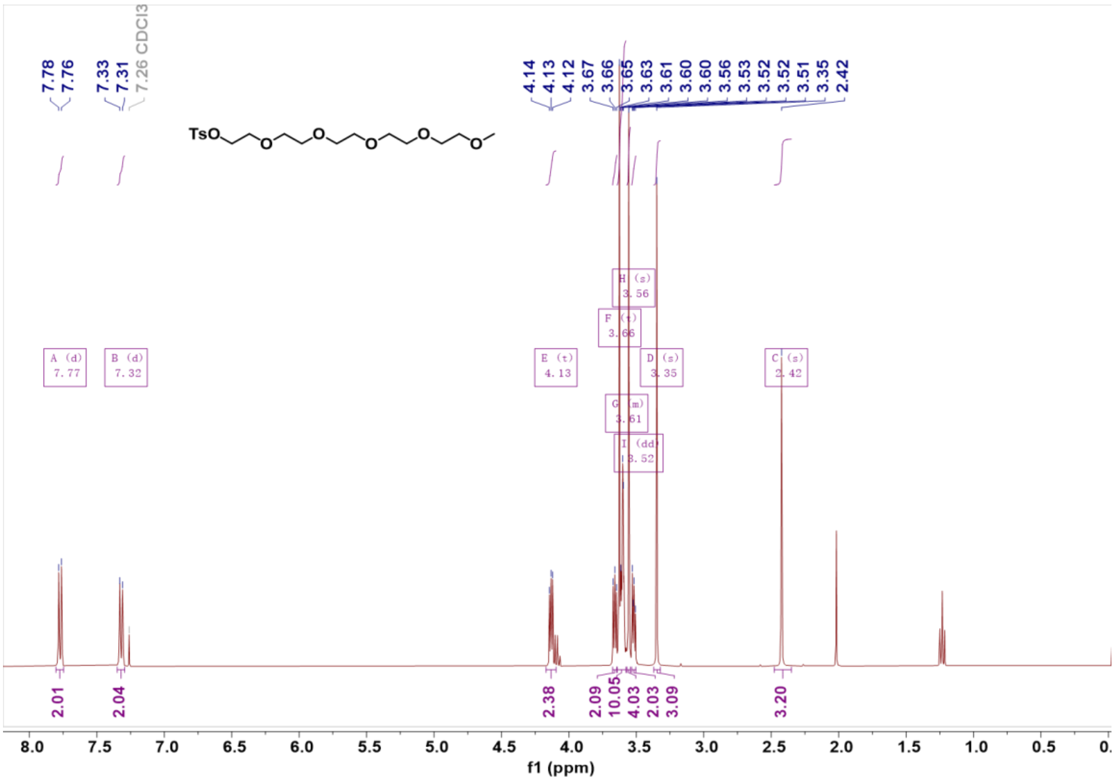

Figure S2: ^{1}H NMR image of compound **M1**.

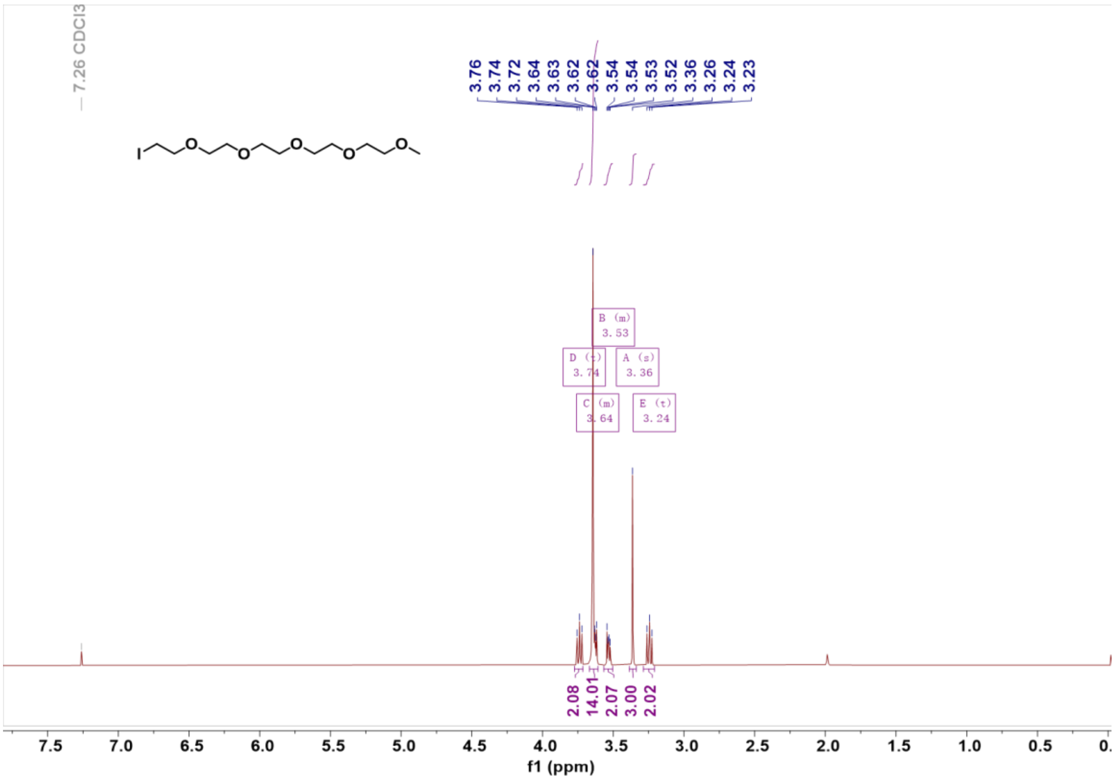

Figure S3: ^{1}H NMR image of compound **M2**.

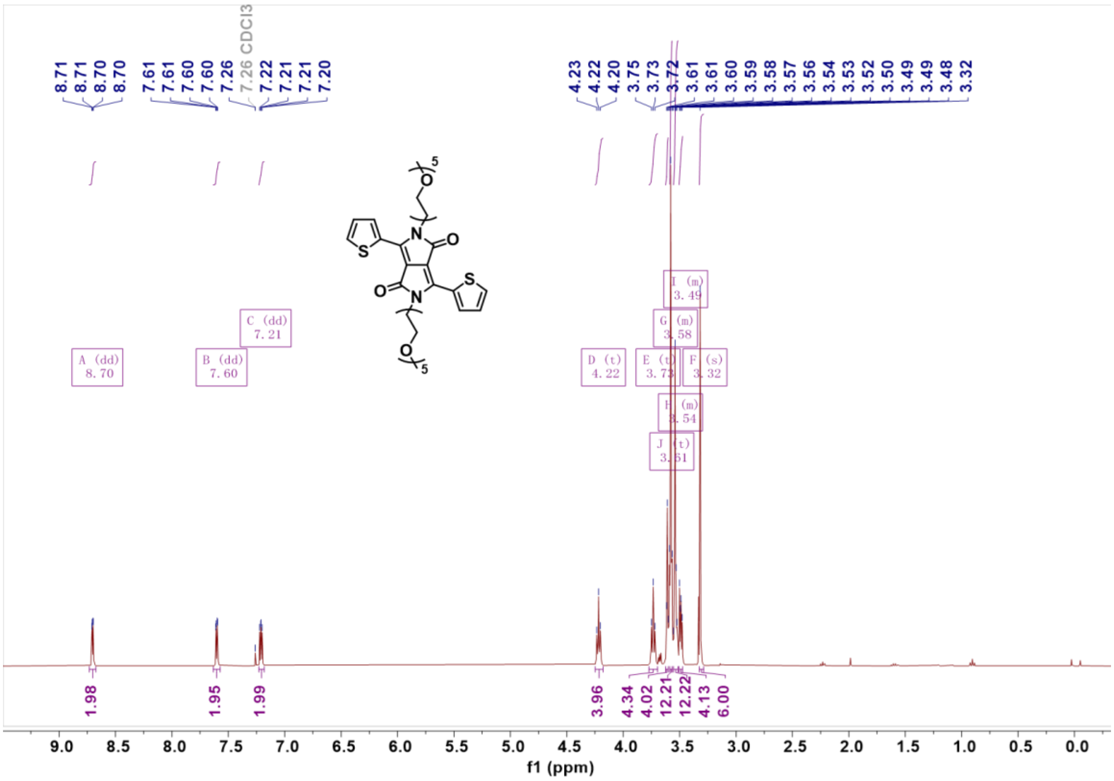

Figure S4: ^{1}H NMR image of compound **M3**.

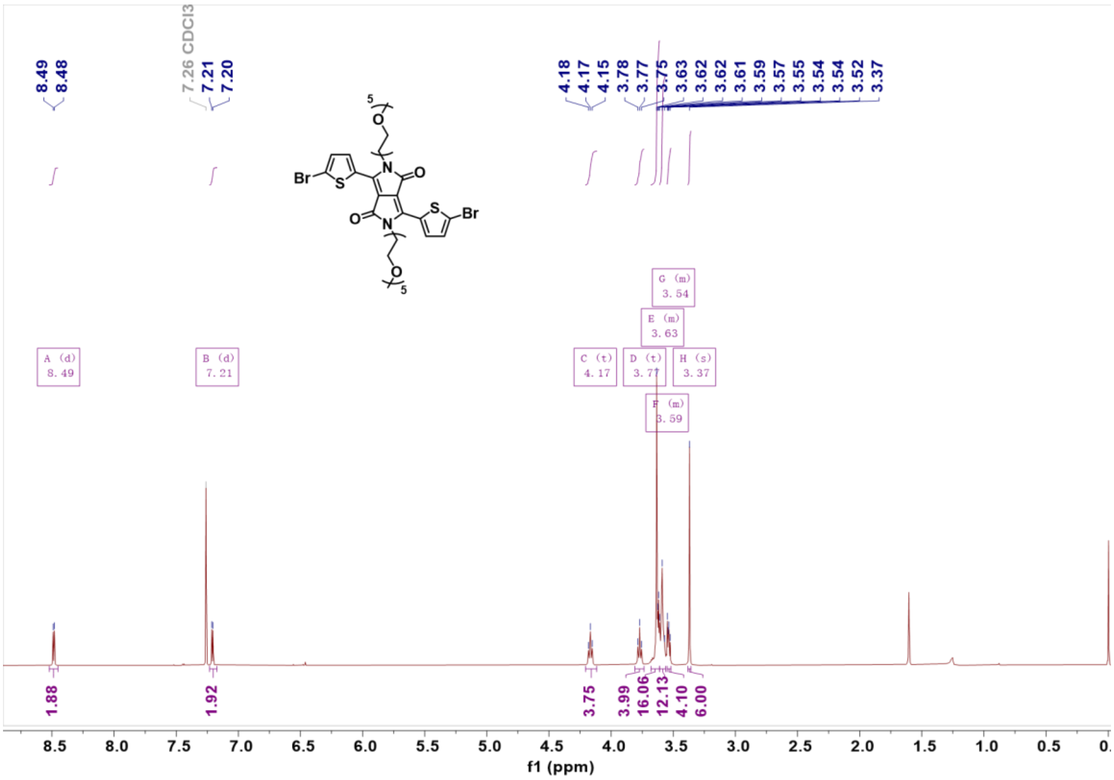

Figure S5: ^{1}H NMR image of compound **M4**.

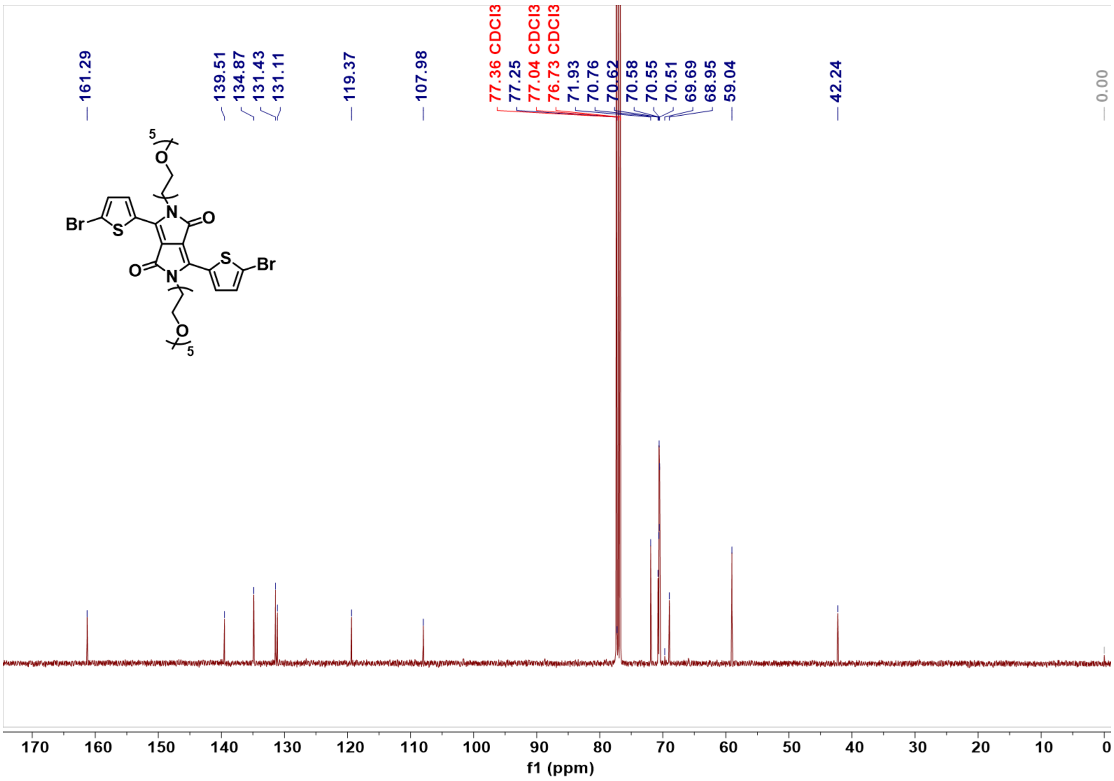

Figure S6: ^{13}C NMR image of compound **M4**.

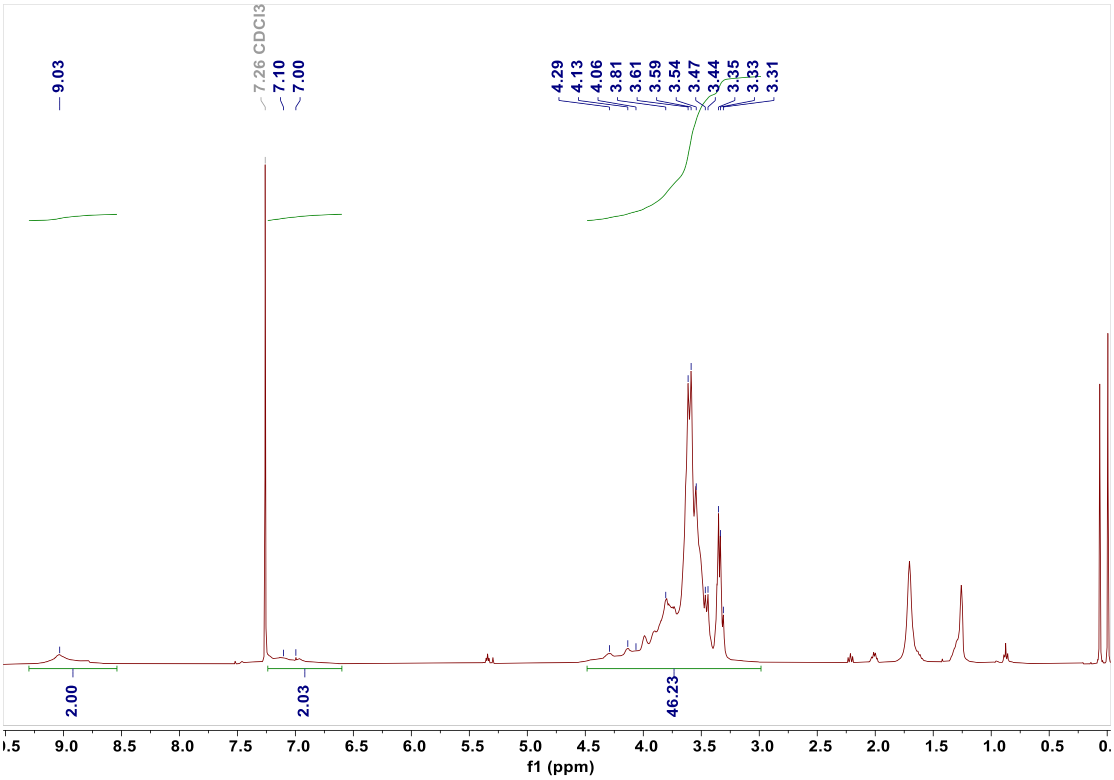

Figure S7: ^{1}H NMR image of compound **S-gDPP**.

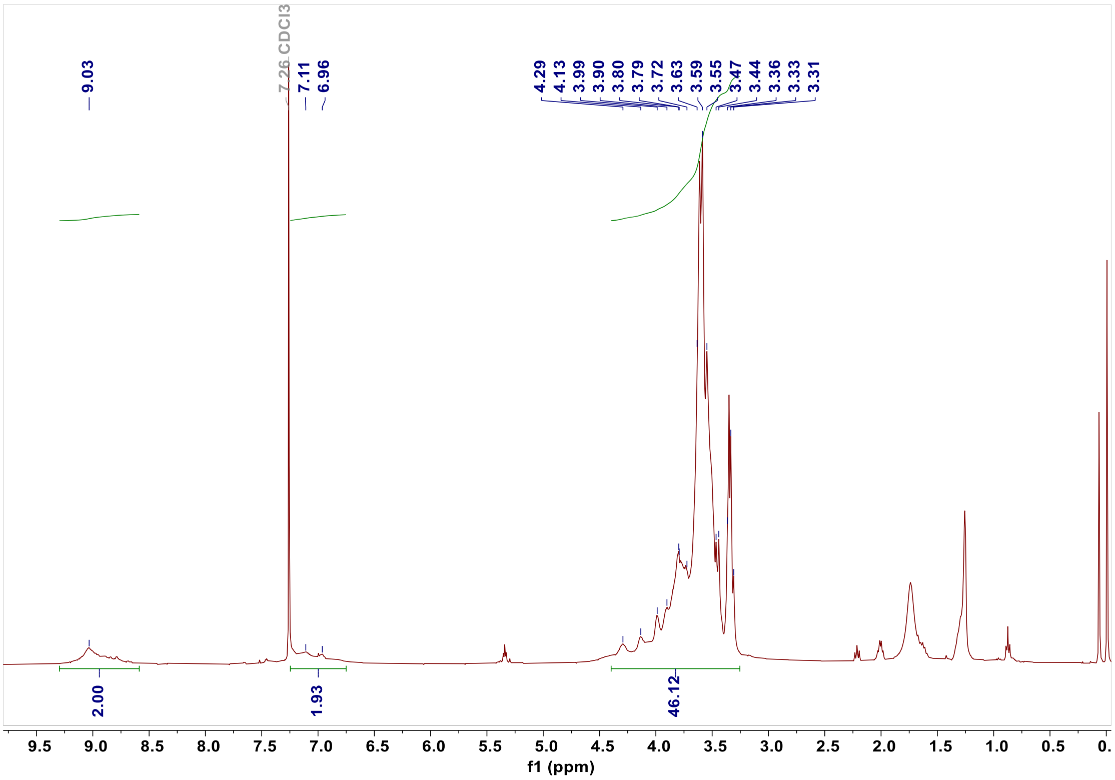

Figure S8: ^{1}H NMR image of compound **M-gDPP**.

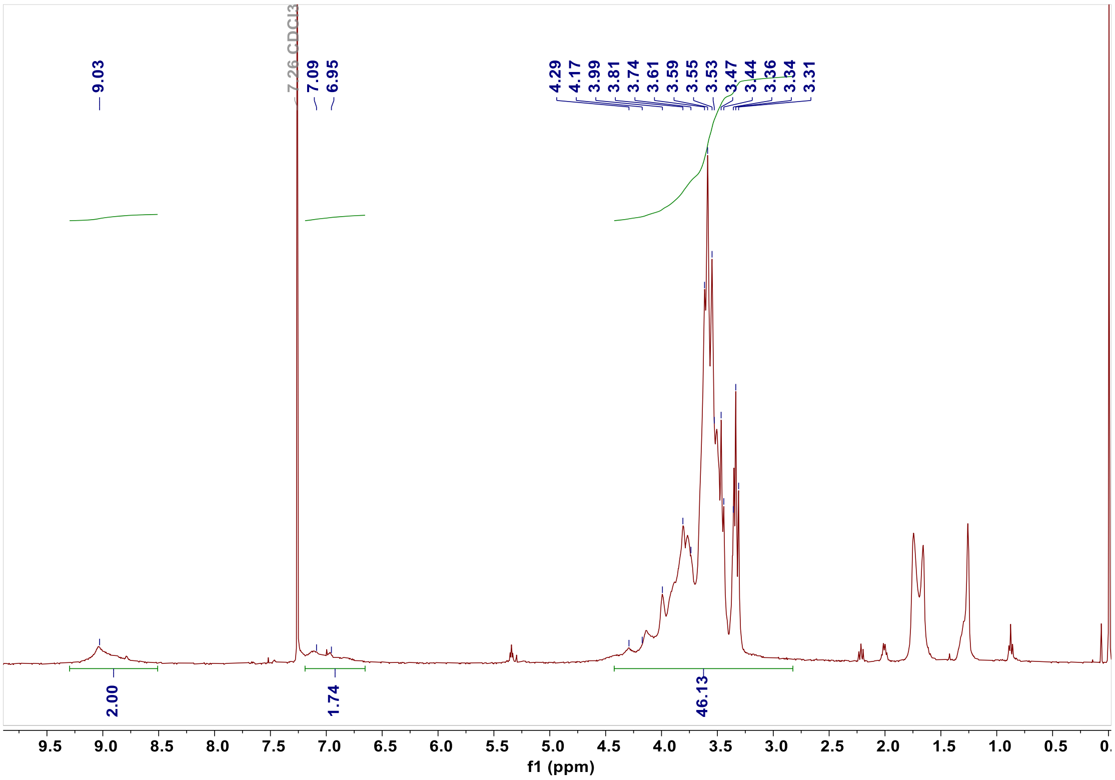

Figure S9: ^{1}H NMR image of compound **H-gDPP**.

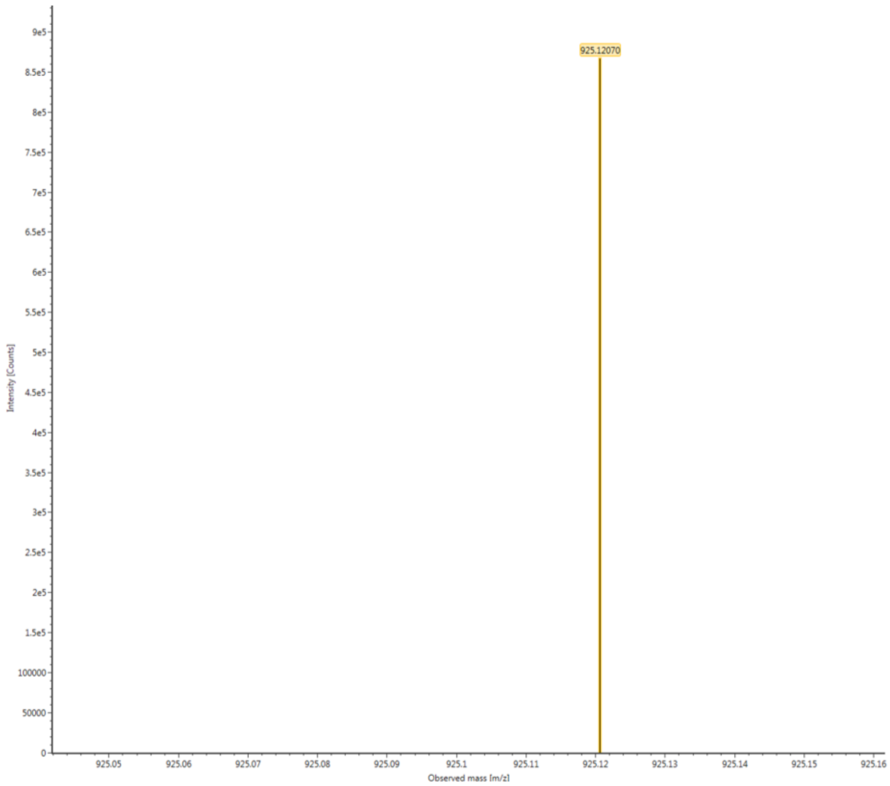

Figure S10: Mass spectrum of compound **M4**.

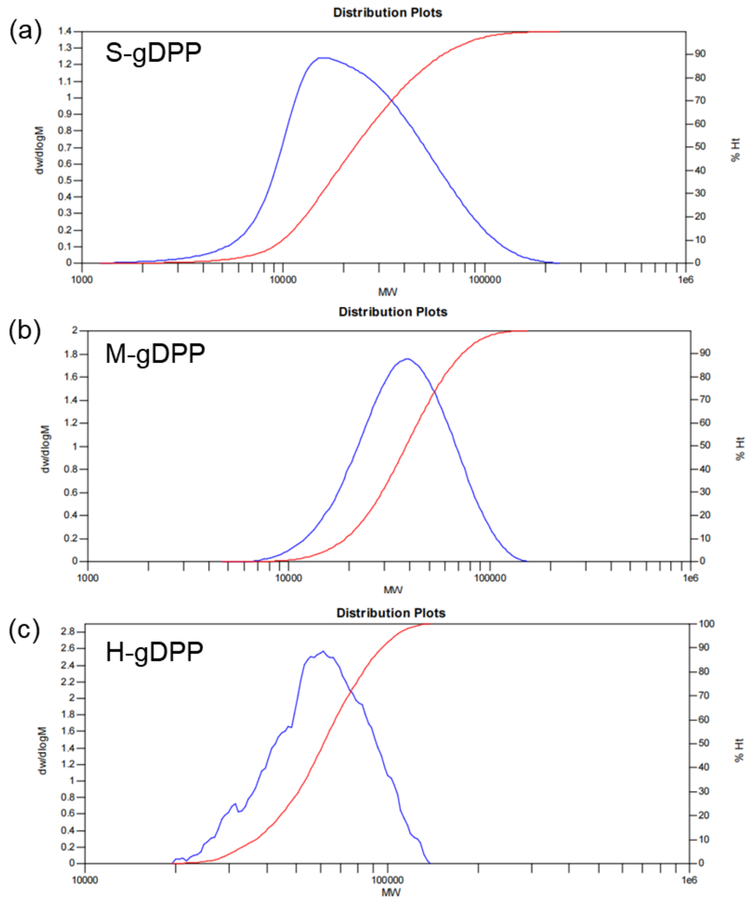

Figure S11. GPC image of HOMO-gDPP polymers. GPC measurements were performed using hexafluoroisopropanol (HFIP) as the eluent.

Table S1. Molecular weights of three HOMO-gDPP polymers. GPC measurements were performed using hexafluoroisopropanol (HFIP) as the eluent.

	**Polymers**	M_n **(kDa)**	M_w **(kDa)**	M_P **(kDa)**	M_z **(kDa)**	**Polydispersity**
1	S-gDPP	19.4	31.9	51.1	51.1	1.6
2	M-gDPP	33.5	43.1	37.5	53.8	1.3
3	H-gDPP	56.0	63.6	61.5	71.3	1.1

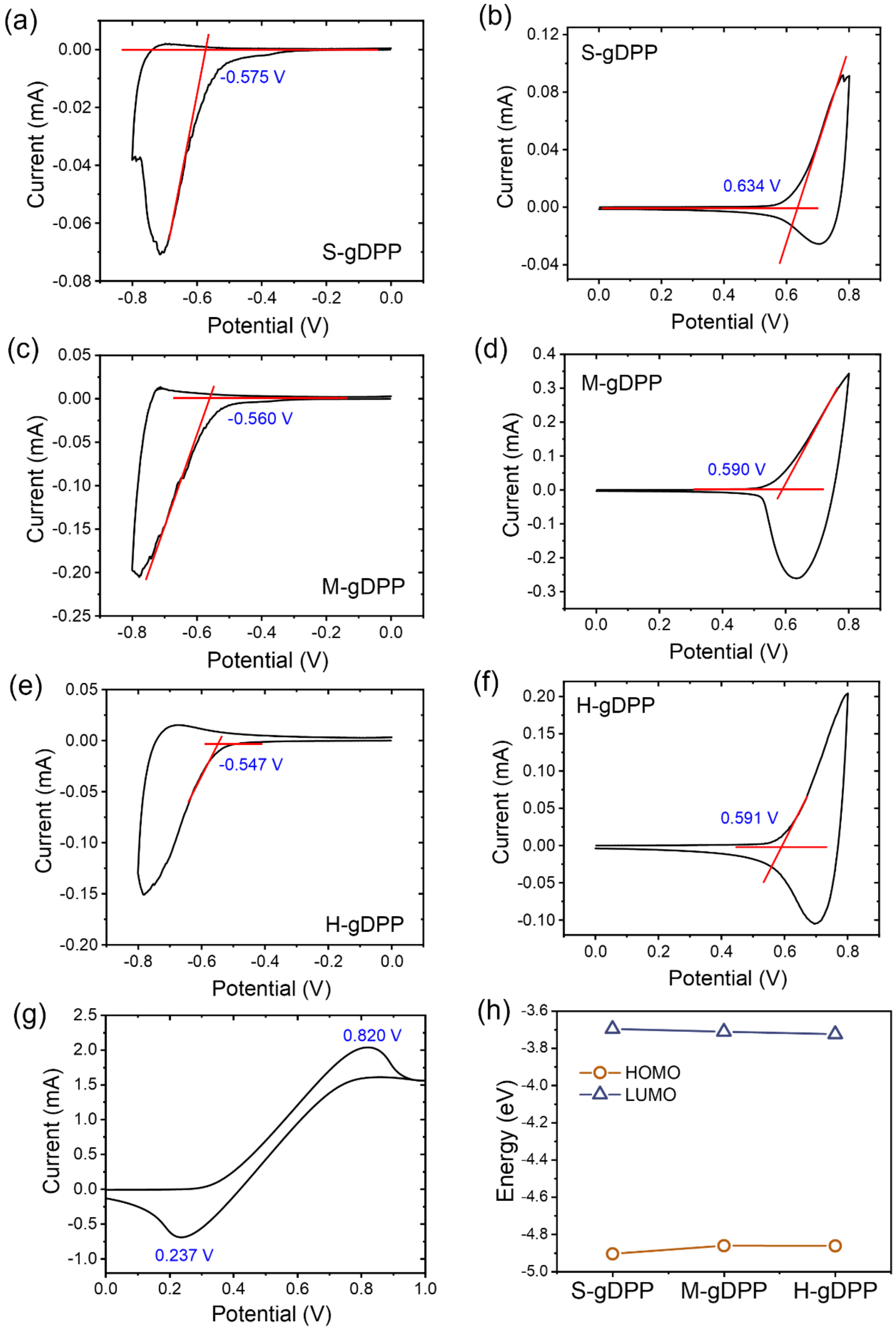

Figure S12. Cyclic voltammetry measurements of HOMO-gDPP polymers tested in 0.1 M tetrabutylammonium hexafluorophosphate acetonitrile solution at a scan rate of 100 mV/s.

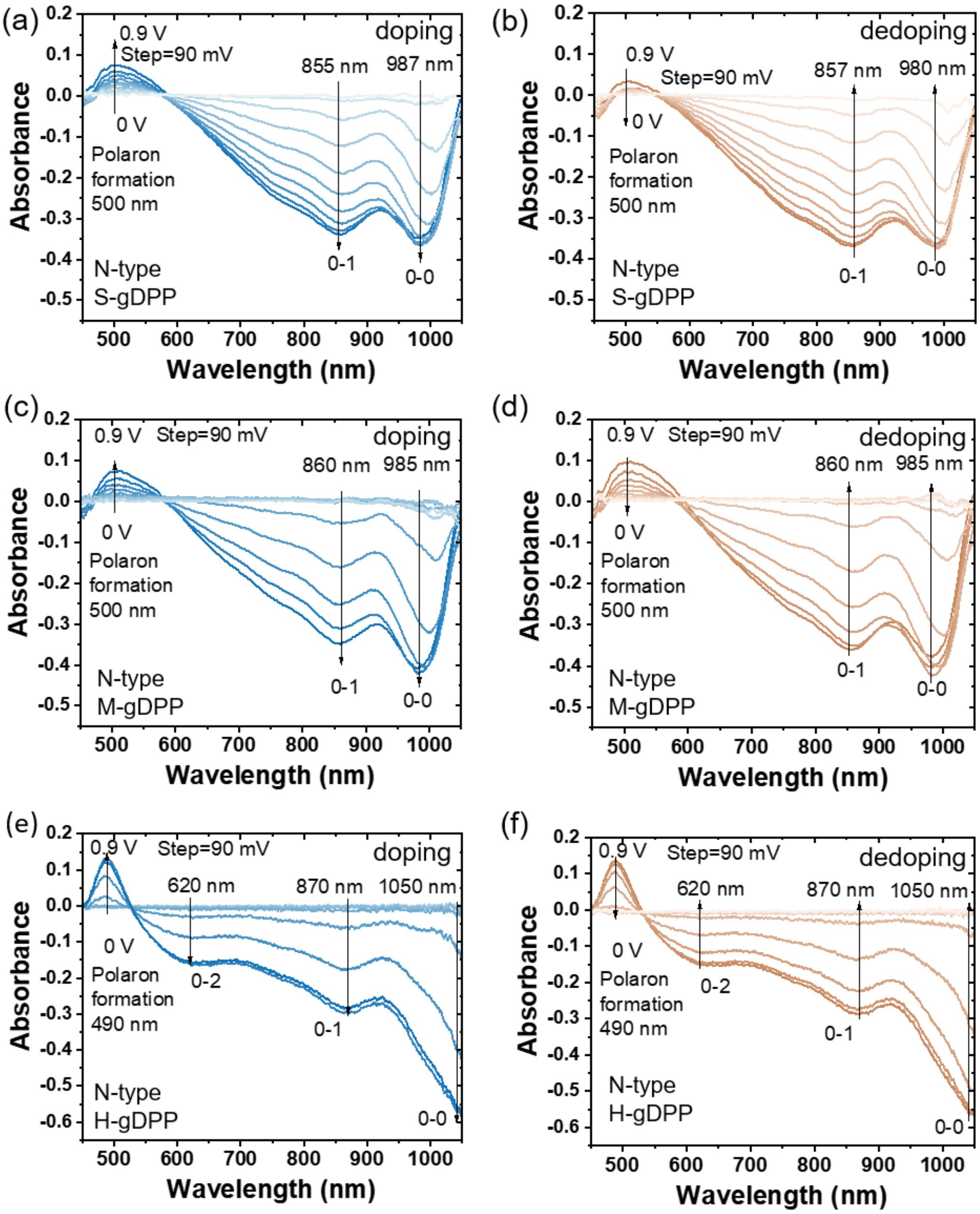

Figure S13. In situ UV-Vis absorption spectral changes during N-type electrochemical doping/dedoping of HOMO-gDPP materials with different molecular weights.

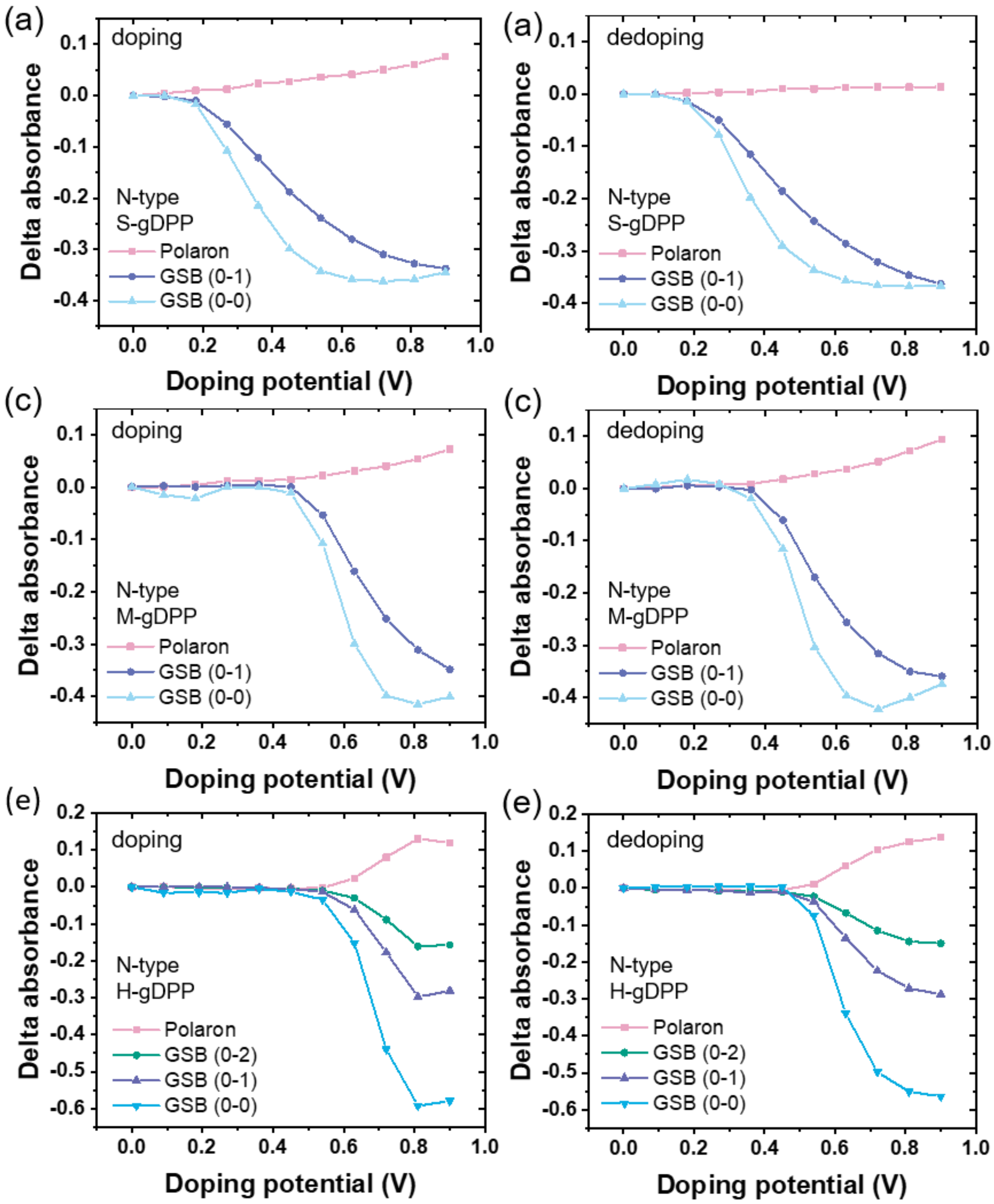

Figure S14. Evolution of absorption intensity of the ground-state bleaching (GSB) and polaron peaks during N-type electrochemical doping/dedoping was recorded as a function of doping potential for HOMO-gDPP with different molecular weights.

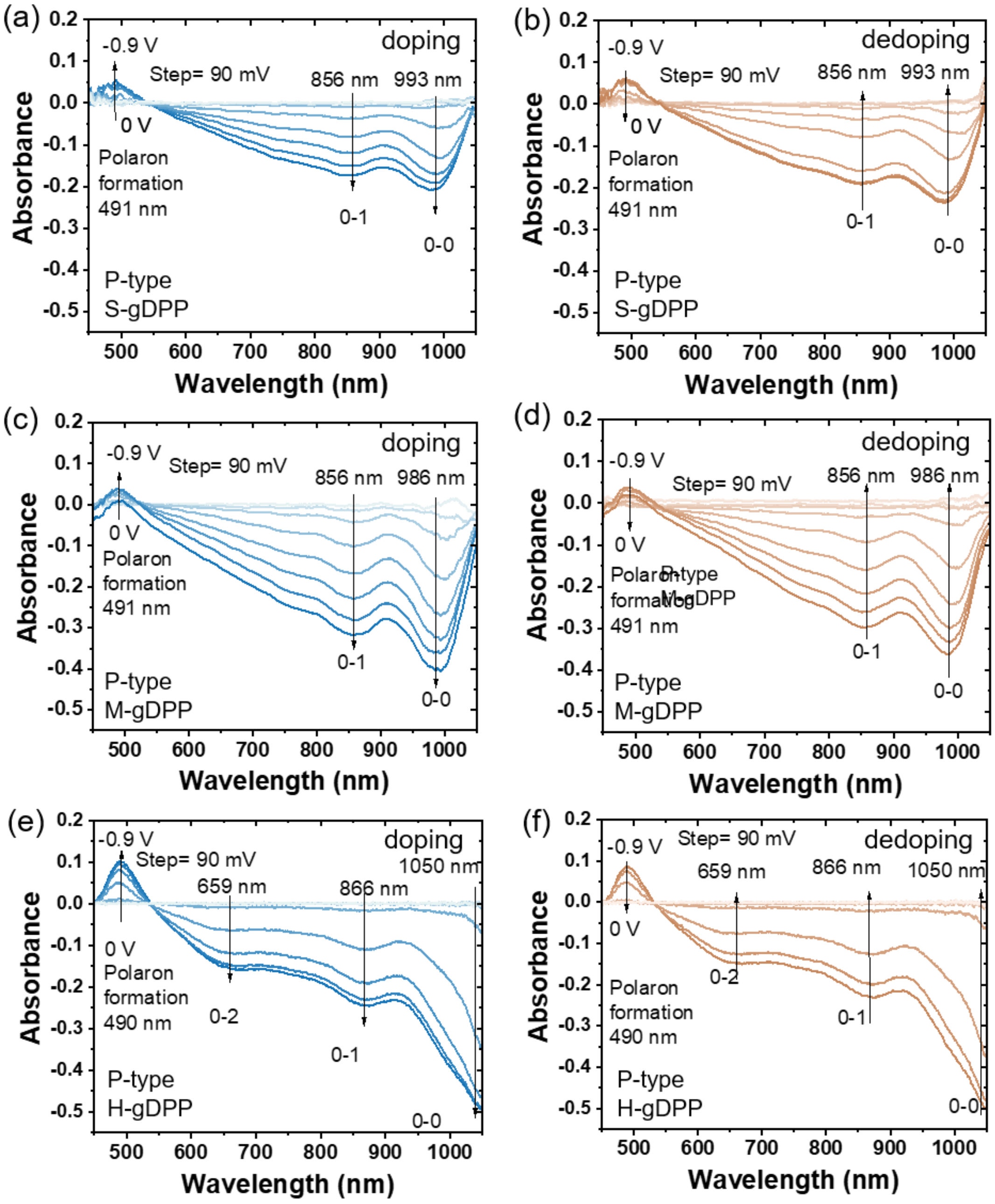

Figure S15. In situ UV-Vis absorption spectral changes during P-type electrochemical doping/dedoping of HOMO-gDPP materials with different molecular weights.

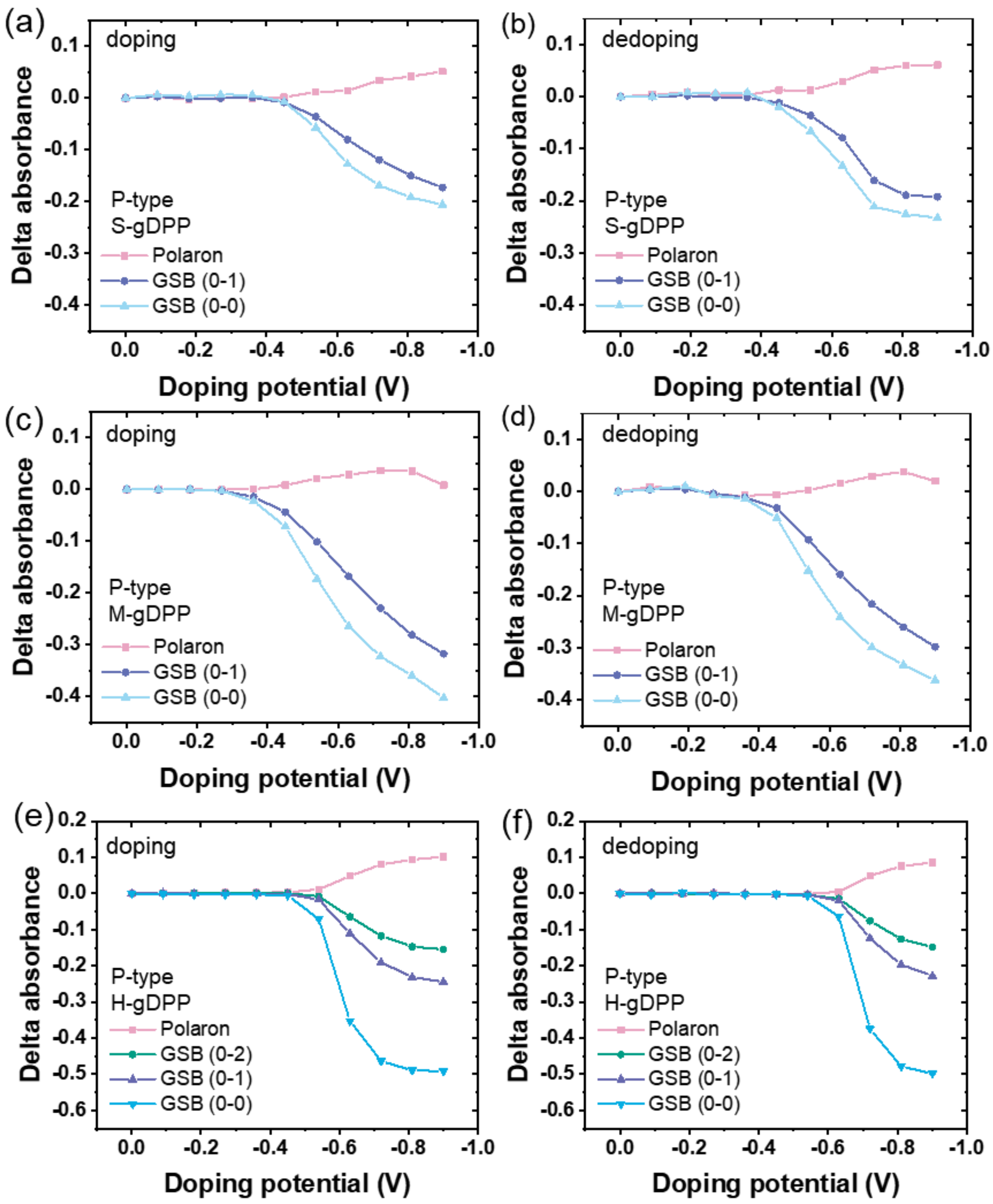

Figure S16. Evolution of absorption intensity of the ground-state bleaching (GSB) and polaron peaks during P-type electrochemical doping/dedoping was recorded as a function of doping potential for HOMO-gDPP with different molecular weights.

Table S2. Film thickness statistics of planar OECTs based on HOMO-gDPP polymers with different molecular weights (*n* = 4 devices).

Name	Thickness 1 (nm)	Thickness 2 (nm)	Thickness 3 (nm)	Thickness 4 (nm)	Average thickness (nm)
S-gDPP	42.2	43.0	37.1	39.1	40.4
	62.7	68.2	76.9	75.4	70.8
	114.3	106.1	106.8	107.3	108.6
	125.8	129.9	134.9	131.3	130.3
M-gDPP	42.8	45.3	49.8	50.5	47.1
	73.7	68.9	76.3	69.2	72.0
	102.5	110.0	118.5	116.2	111.8
	121.6	133.5	130.2	136.2	130.4
H-gDPP	48.2	52.5	57.8	58.3	54.2
	84.4	89.0	96.5	98.9	92.2
	122.4	128.5	133.5	138.4	130.7
	181.7	176.9	185.4	175.1	179.8

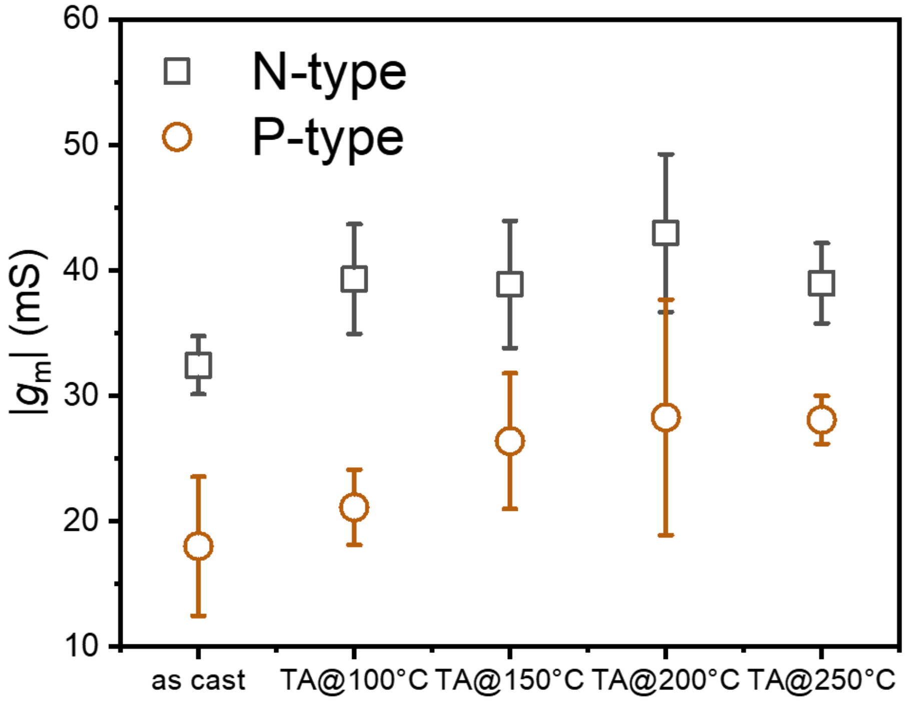

Figure S17. Transconductance of H-gDPP-based OECTs with different annealing temperatures (n=4 devices for each temperature, W=1000 μm, L=50 μm, d≈180 nm).

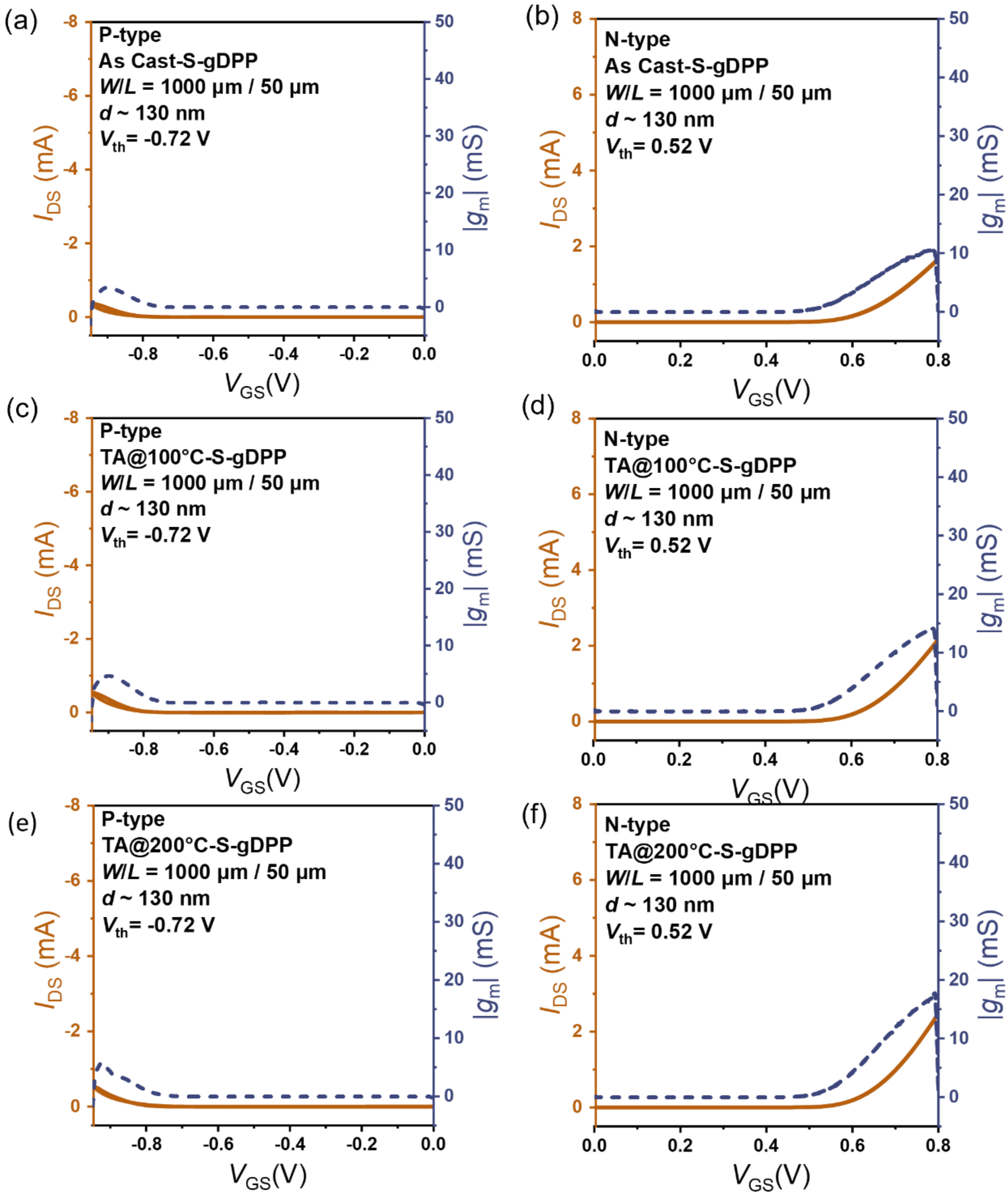

Figure S18. Transfer curves of the S-gDPP-based OECTs with different annealing temperatures.

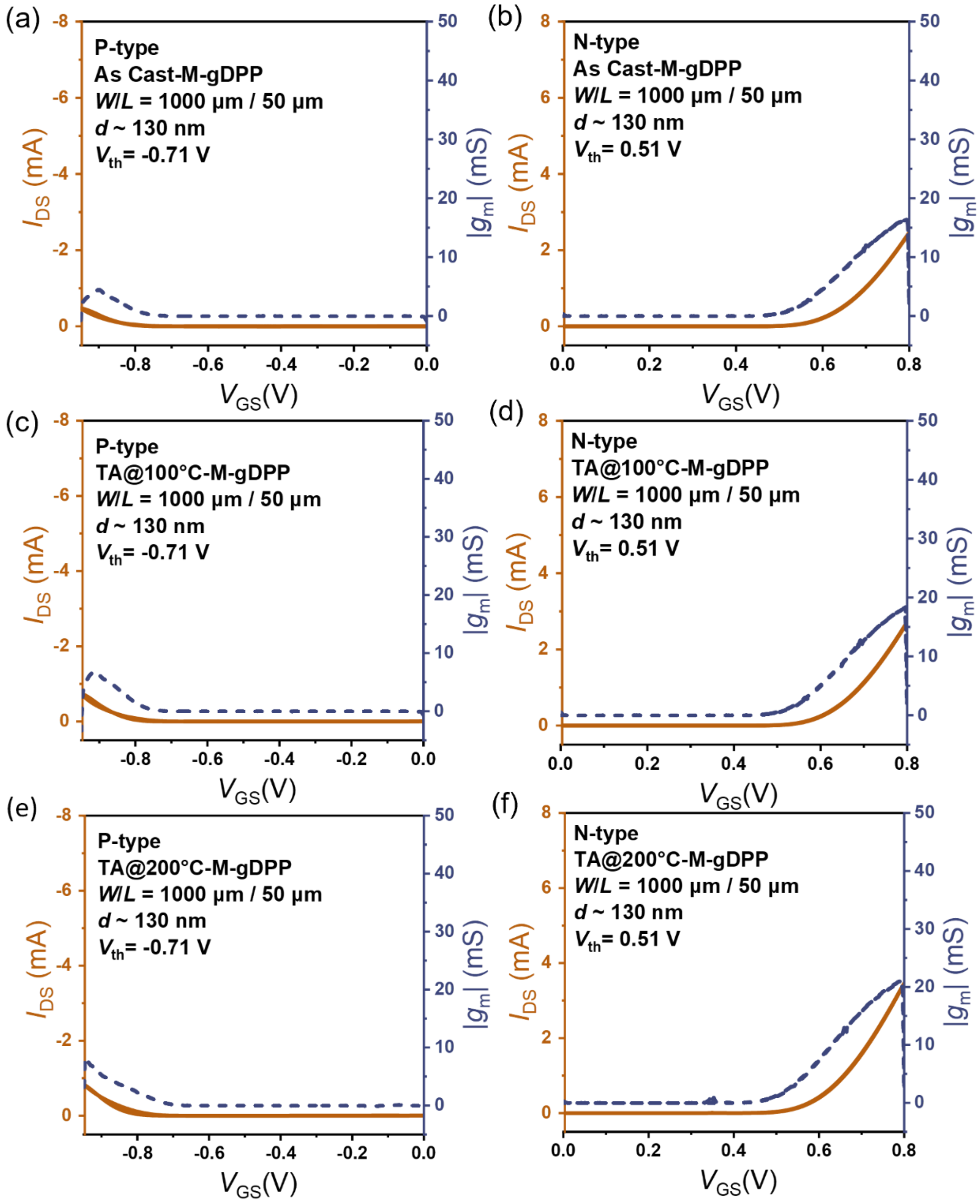

Figure S19. Transfer curves of the M-gDPP-based OECTs with different annealing temperatures.

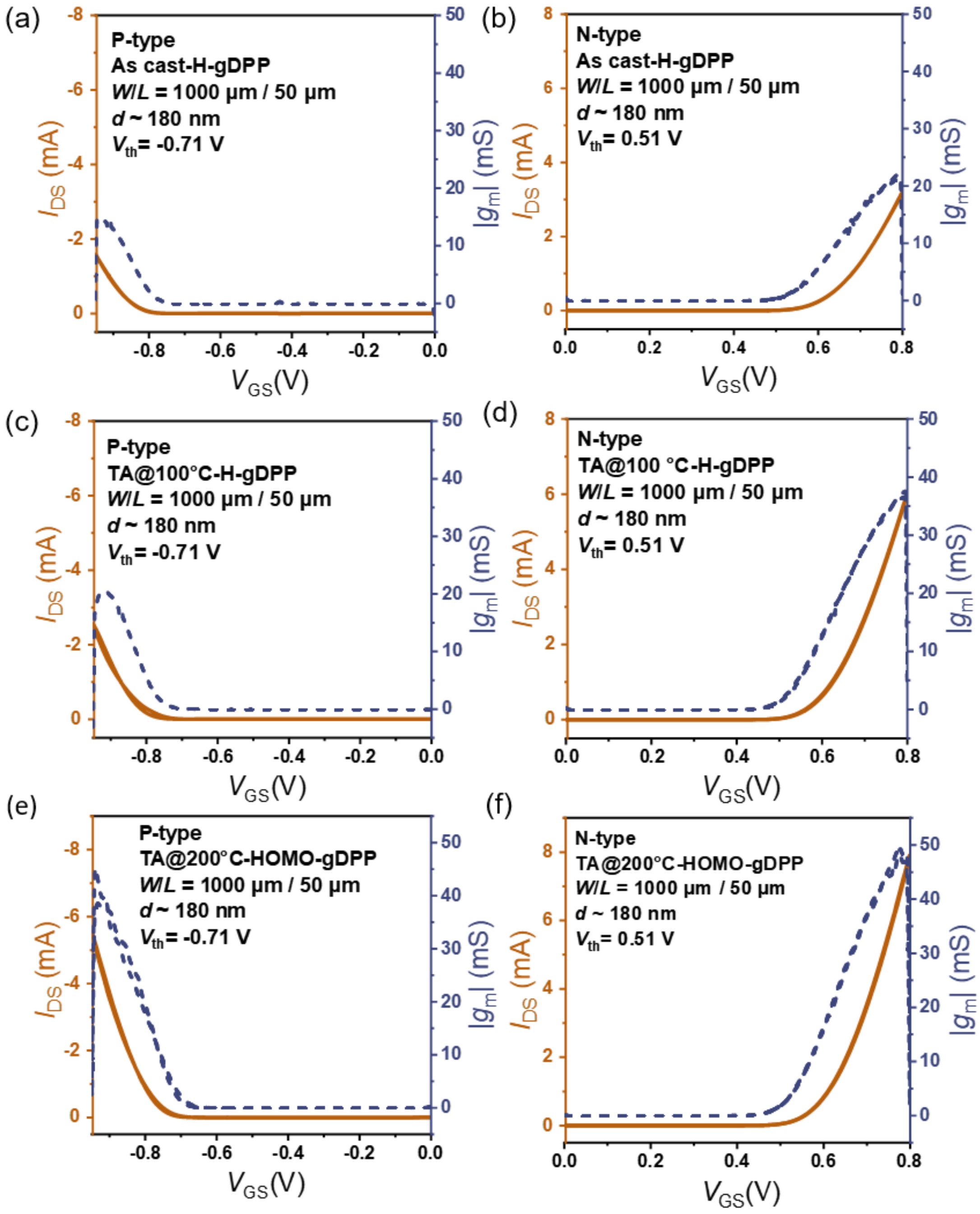

Figure S20. Transfer curves of the H-gDPP-based OECTs with different annealing temperatures.

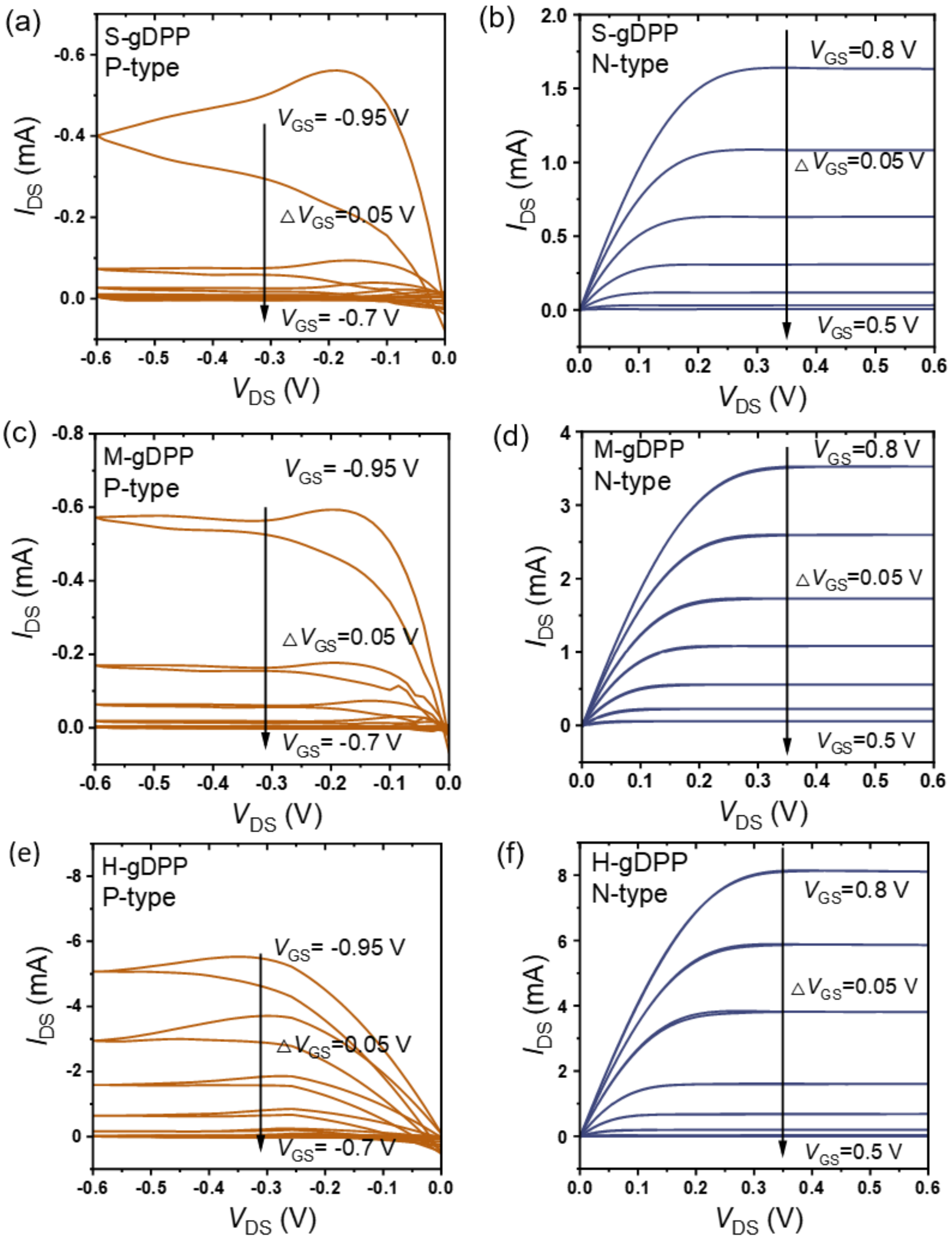

Figure S21. Output curves of the 200°C annealed HOMO-gDPP-based OECTs.

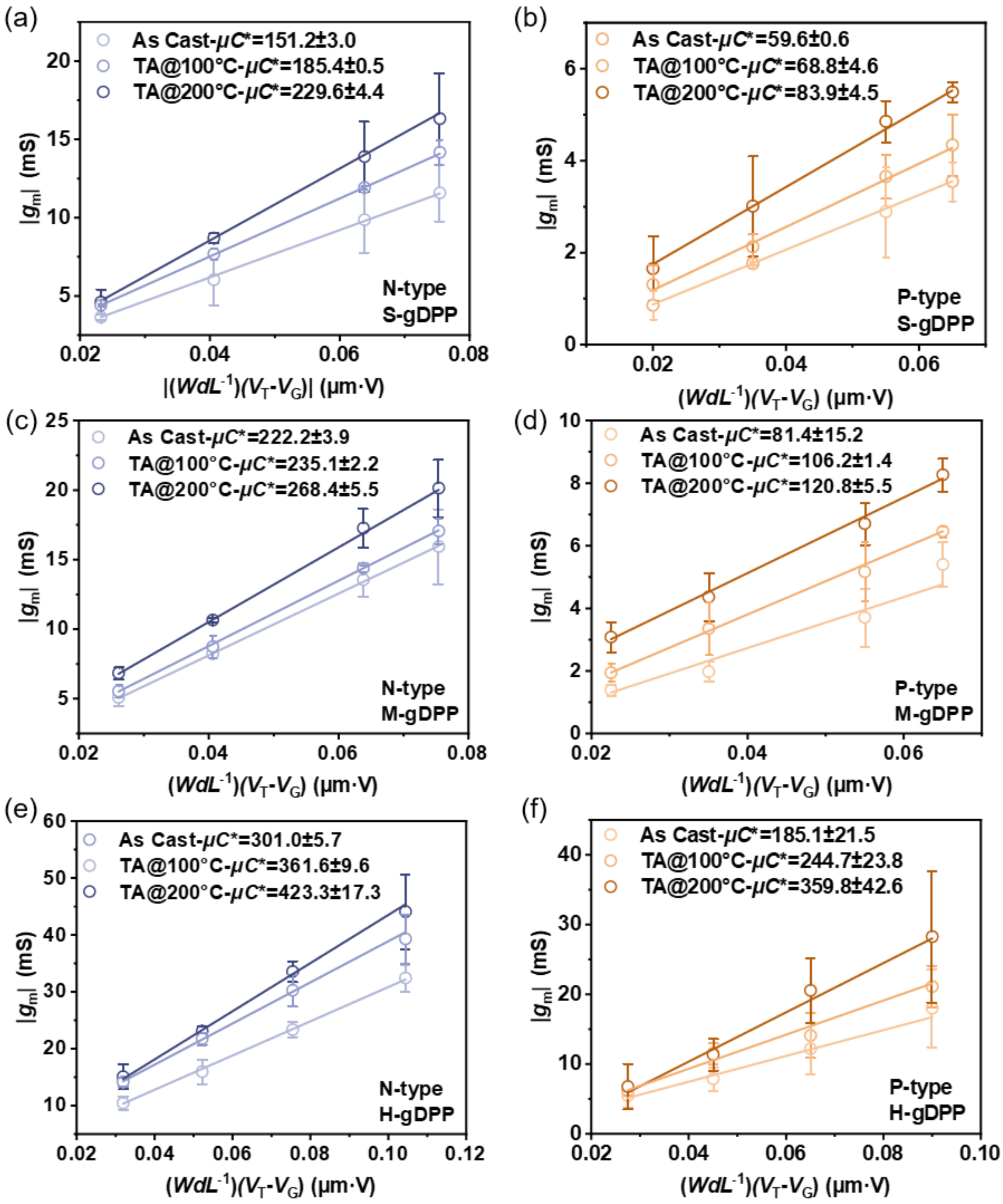

Figure S22. Schematic illustration of the figures of merit (μC^*) fitting for three polymers with different molecular weights under n-type and p-type characteristics. Error bars denote the s.d. for $n = 4$ devices, mean ± s.d.

Table S3. Device parameters of as-cast S-gDPP, M-gDPP and H-gDPP-based OECTs. The different thickness data used for fitting are shown in **Fig. S22**, while the table lists only the representative-thickness device performance.

**Polymer** [a)]	**Polarity**	**S-gDPP**	**M-gDPP**	**H-gDPP**
$g_{m\ max}$ (mS)	N	11.59±1.80	15.95±2.06	32.45±2.31
	P	3.55±0.43	5.41±0.72	18.02±5.54
d (nm)	N<br>P	130±5	130±6	180±5
V_{th} (V)	N	0.52±0.01	0.51±0.01	0.51±0.01
	P	−0.72±0.02	−0.71±0.01	−0.71±0.01
μC^* (F cm^{-1} V^{-1} s^{-1}) [b)]	N	151.2±3.0	222.2±3.9	301.0±5.7
	P	59.6±0.6	81.4±15.2	185.1±21.5
μ (cm^2 V^{-1} s^{-1}) [c)]	N	1.28±0.06	1.37±0.04	1.67±0.08
	P	0.45±0.09	0.55±0.02	0.98±0.04
C^* (F cm^{-3}) [d)]	N	88.06±5.96	106.77±9.63	134.23±6.25
	P	49.08±4.80	65.22±6.12	113.85±19.32
on/off ratio	N	10^4	10^4	10^4
	P	10^3	10^3	10^4

a) Channel dimension: W=1000 μm, L=50 μm. b) Calculated by linear fitting the g_m with different channel dimensions. c) Calculated using the pulsed gate current injection method. d) Measurement from EIS.

Table S4. Device parameters of 100 °C annealed S-gDPP, M-gDPP and H-gDPP-based OECTs. The different thickness data used for fitting are shown in **Fig. S22**, while the table lists only the representative-thickness device performance.

**Polymer** [a]	**Polarity**	**S-gDPP**	**M-gDPP**	**H-gDPP**
$g_{m\ max}$ (mS)	N	14.19±0.81	17.06±0.90	39.33±4.36
	P	4.35±0.67	6.47±0.17	21.12±3.03
d (nm)	N<br>P	130±5	130±6	180±5
V_{th} (V)	N	0.52±0.01	0.51±0.01	0.51±0.01
	P	−0.72±0.02	−0.71±0.01	−0.71±0.01
μC^* (F cm^{-1} V^{-1} s^{-1}) [b]	N	185.4±0.5	235.1±2.2	361.6±9.6
	P	68.8±4.6	106.2±1.4	244.7±23.8
μ (cm^2 V^{-1} s^{-1}) [c]	N	1.36±0.09	1.51±0.03	2.08±0.09
	P	0.50±0.02	0.69±0.04	1.20±0.04
C^* (F cm^{-3}) [d]	N	100.46±9.72	116.51±15.37	155.42±14.60
	P	75.68±6.07	95.17±9.00	140.45±13.47
on/off ratio	N	10^4	10^4	10^5
	P	10^3	10^3	10^4

a) Channel dimension: W=1000 μm, L=50 μm. b) Calculated by linear fitting the g_m with different channel dimensions. c) Calculated using the pulsed gate current injection method. d) Measurement from EIS.

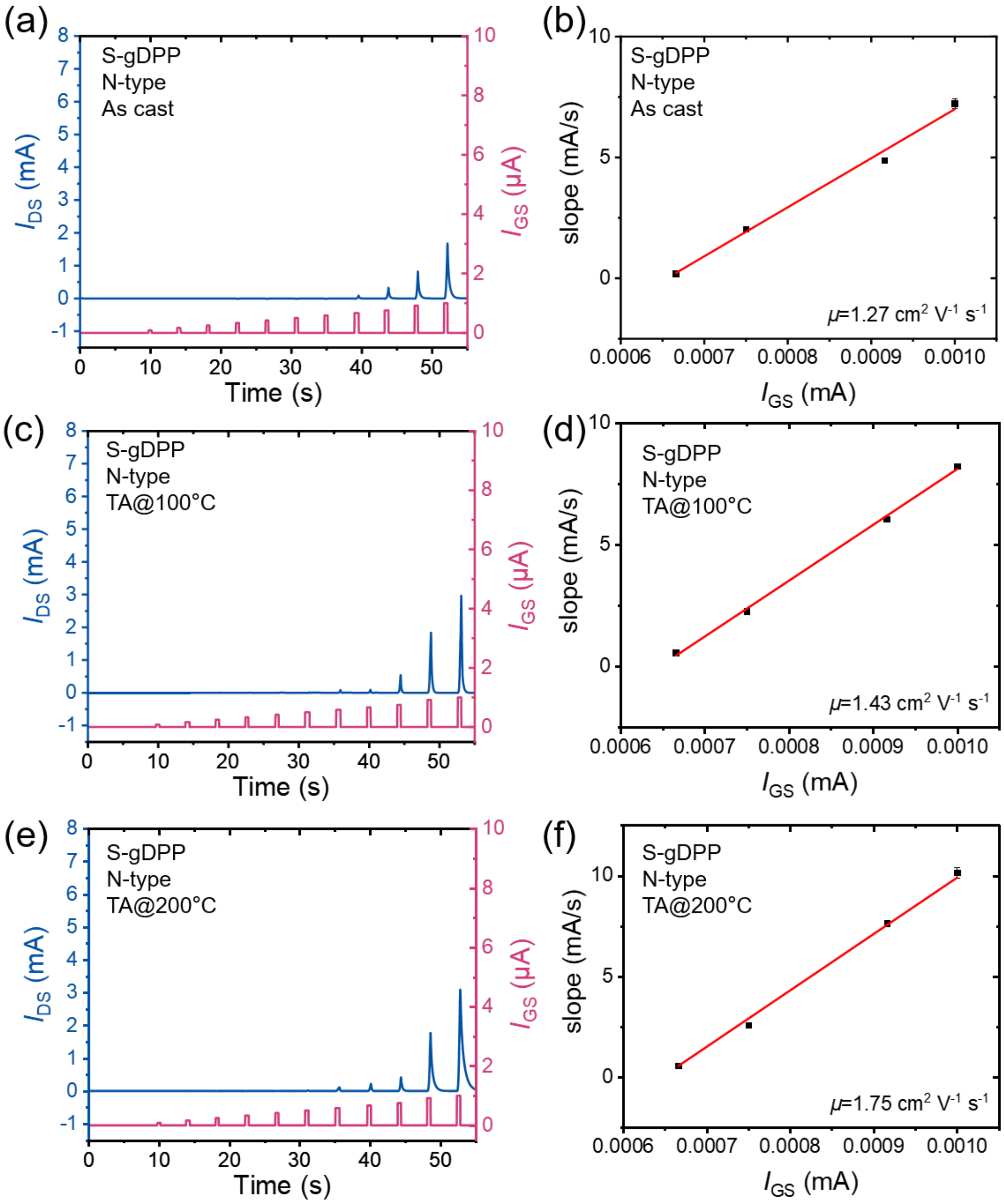

Figure S23. Electron mobility measurement of S-gDPP.

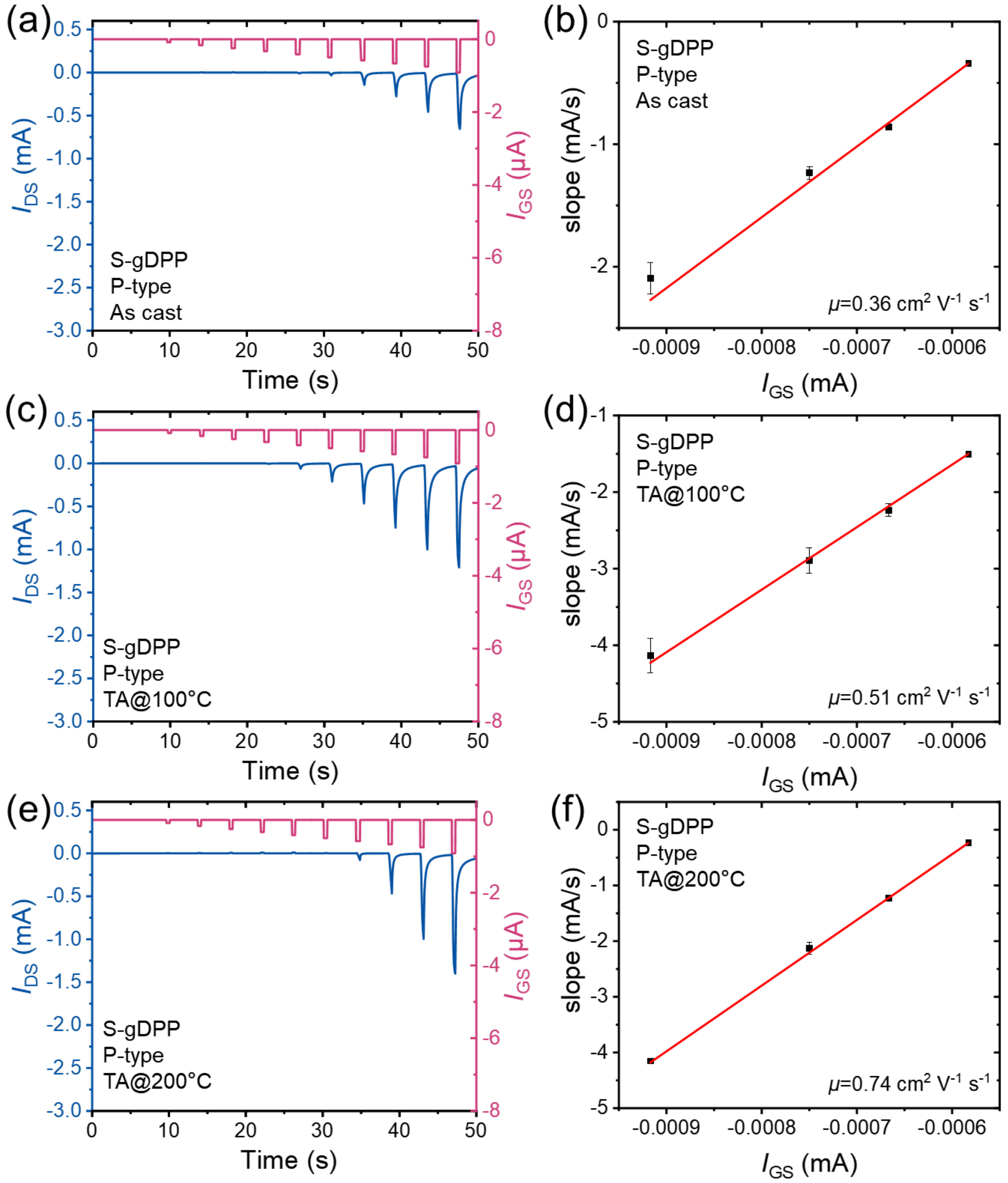

Figure S24. Hole mobility measurement of S-gDPP.

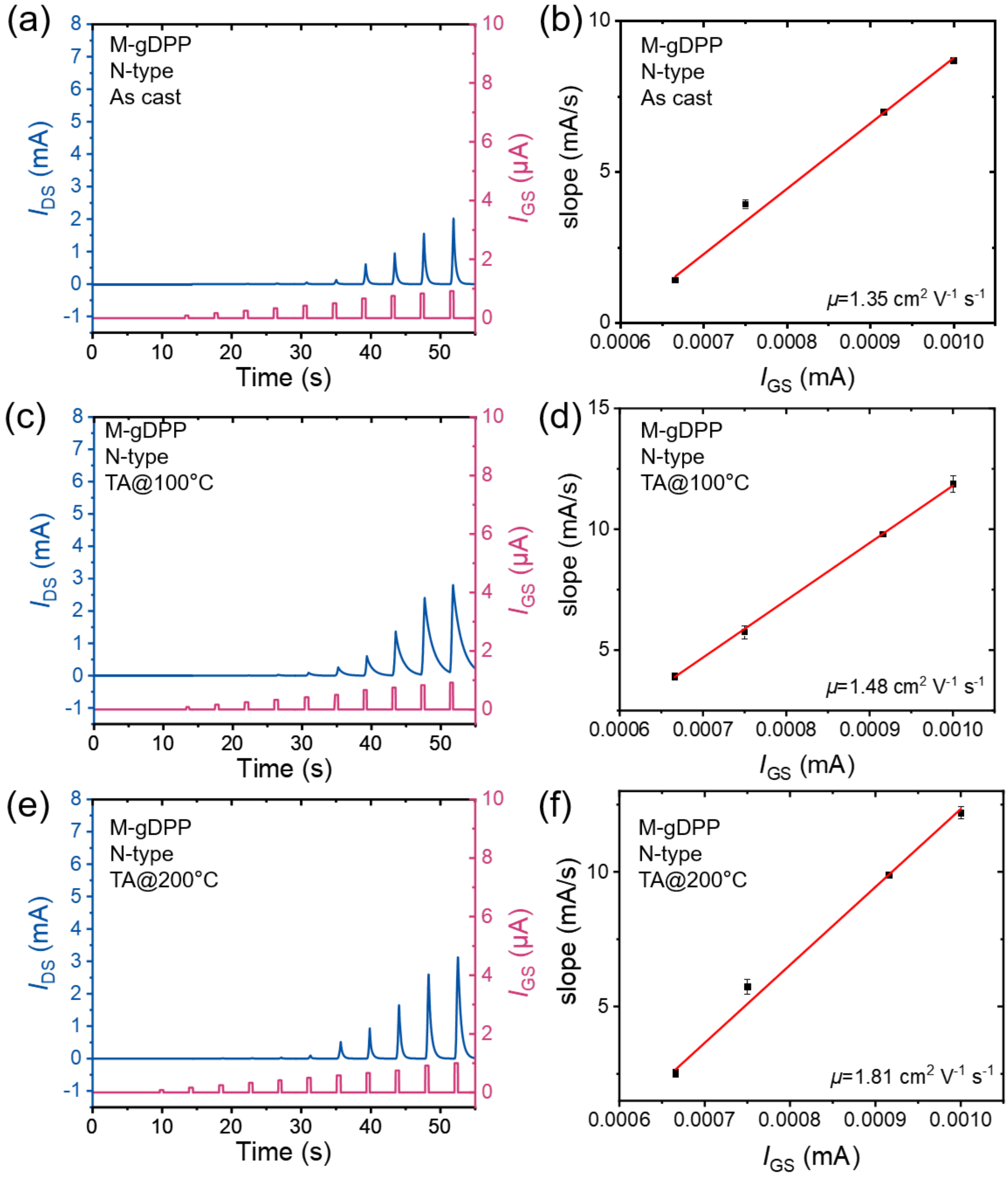

Figure S25. Electron mobility measurement of M-gDPP.

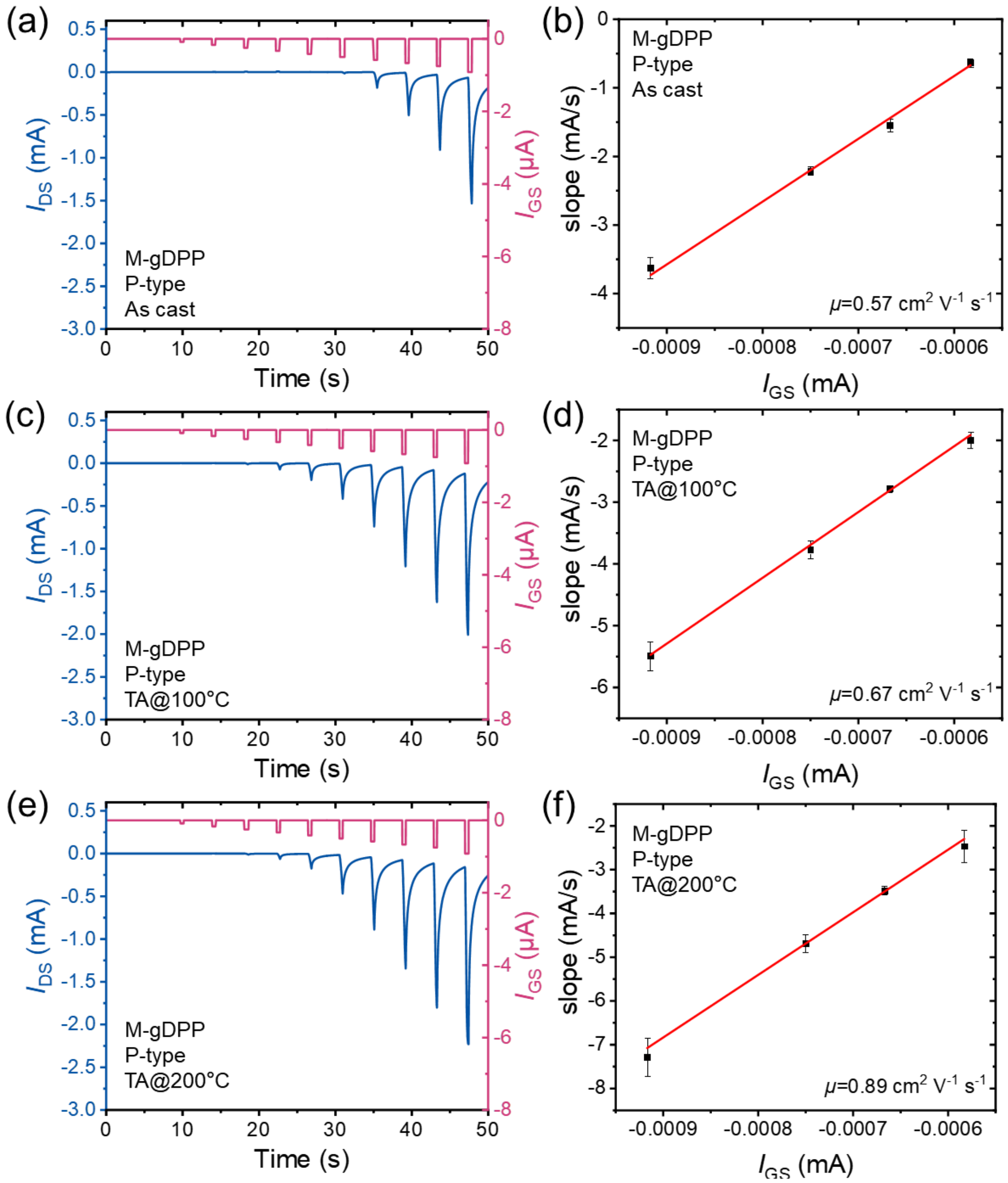

Figure S26. Hole mobility measurement of M-gDPP.

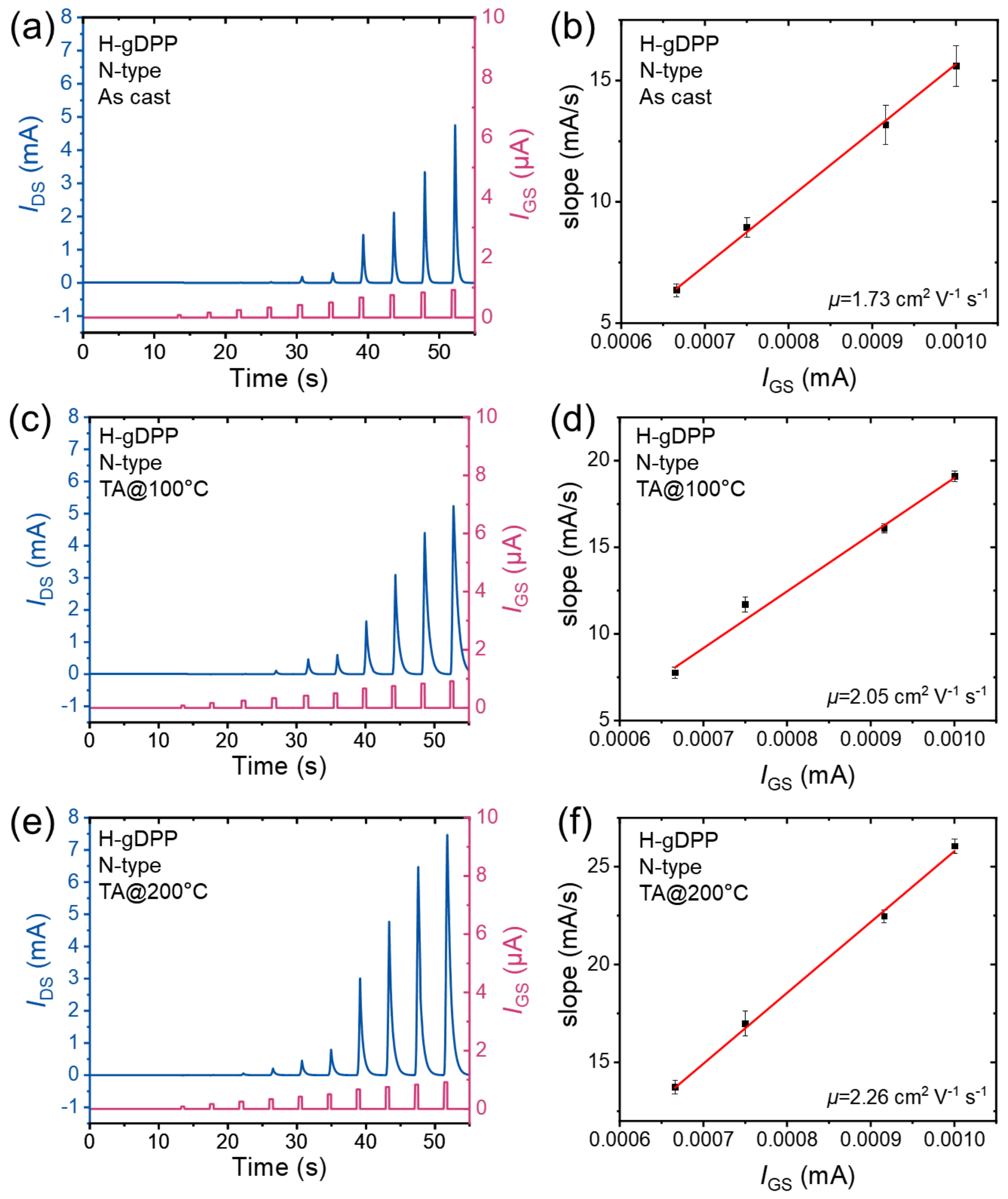

Figure S27. Electron mobility measurement of H-gDPP.

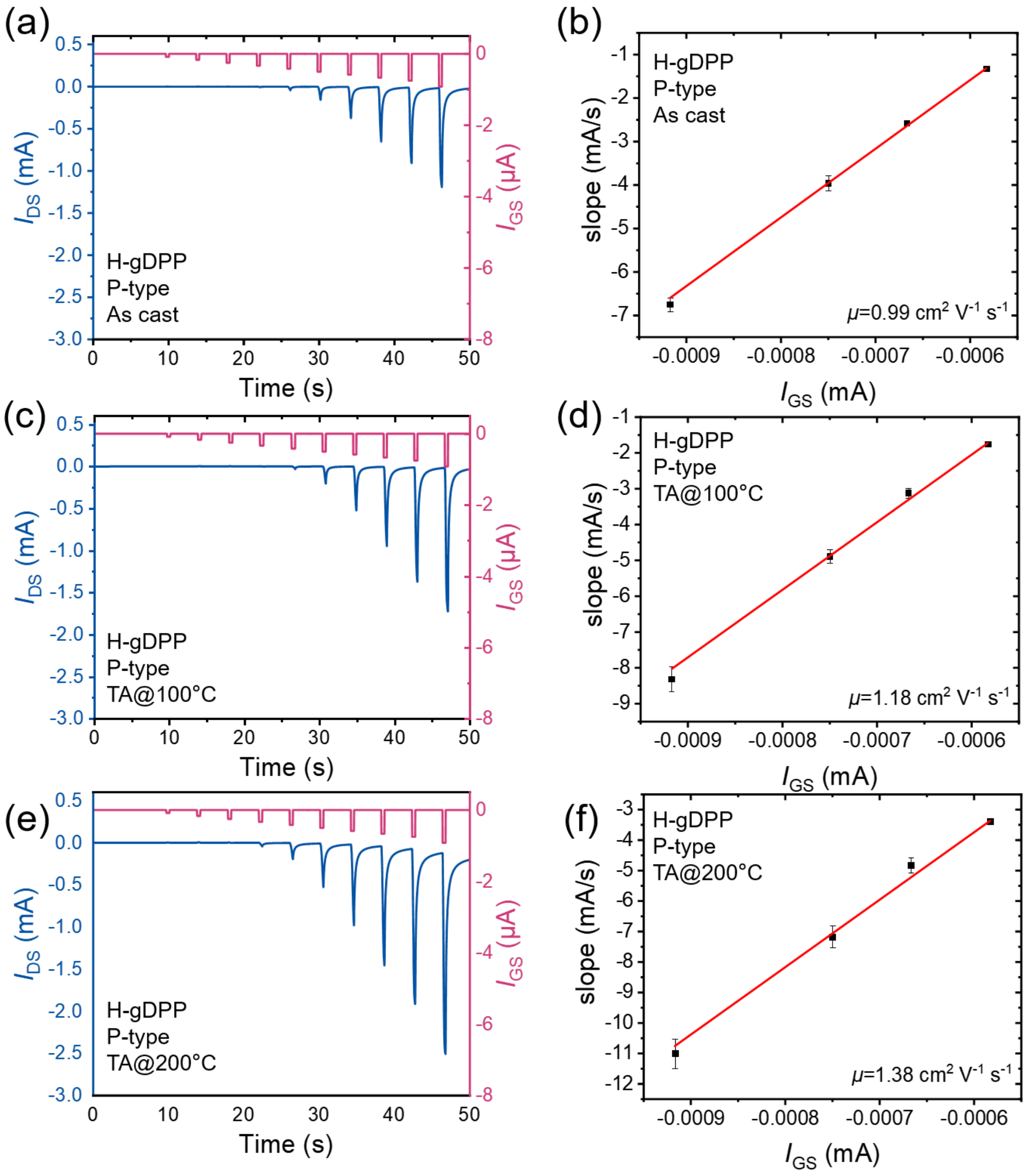

Figure S28. Hole mobility measurement of H-gDPP.

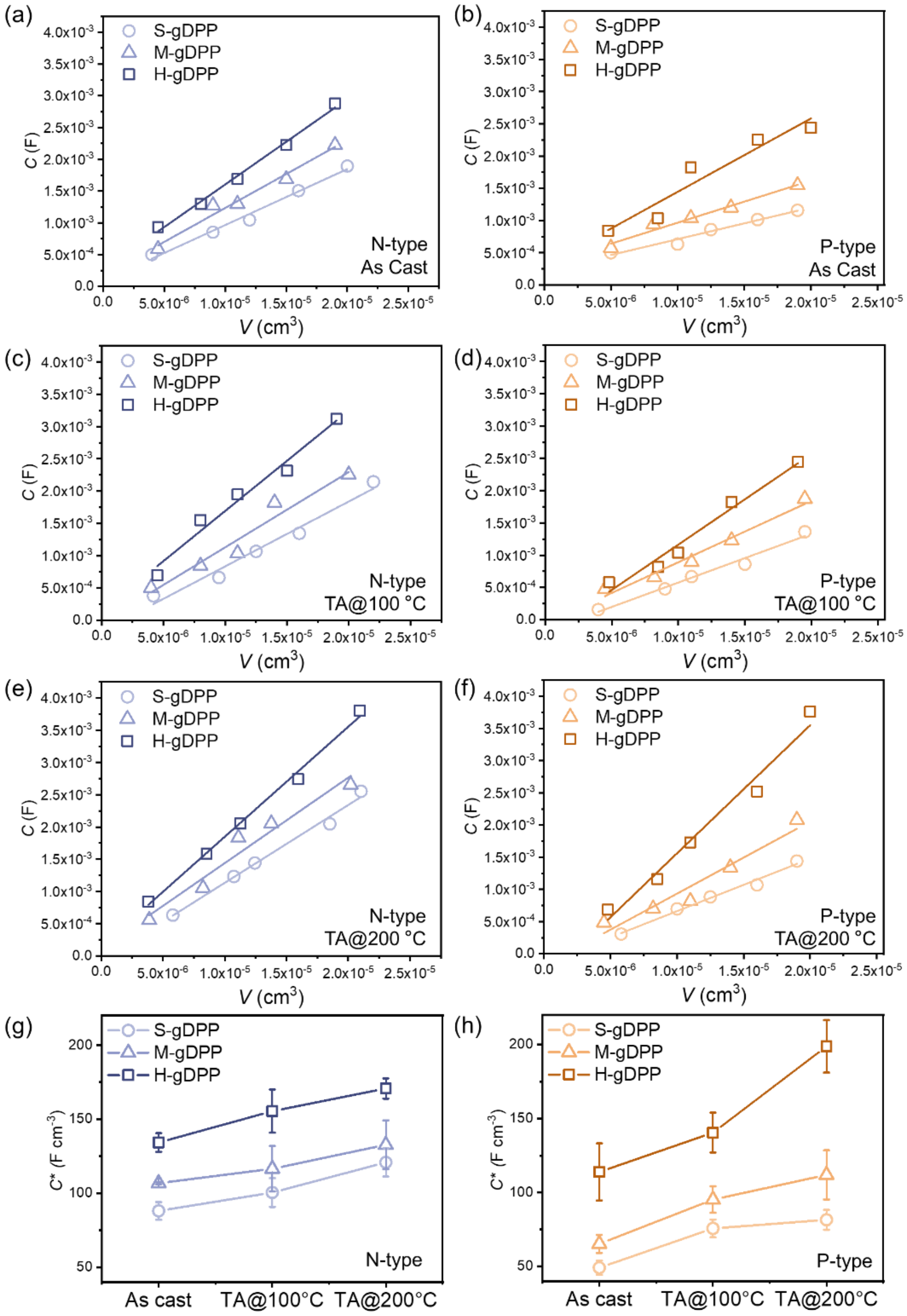

Figure S29. Fitting curves of volumetric capacitance for HOMO-gDPP films with different molecular weights.

Table S5. Summary of reported ambipolar planar OECT performances.

Polymer	Polarity	μC^* (F cm^{-1} V^{-1} s^{-1})	μ_{OECT} (cm^2 V^{-1} s^{-1})	Ref.
P(gNDI-gT2)	P	N/A	N/A	[8]
	N	0.18	N/A	
2DPP-OD-TEG	P	31.8	N/A	[9]
	N	6.8	N/A	
P(C_4-T2-C0-EG)	P	0.13	0.0012	[10]
	N	0.16	N/A	
P(C_4-T2-C0-EG)	P	~0.2	N/A	[11]
	N	0.22	N/A	
PrC60MA:p(g2T-TT) 95:5	P	22.8	N/A	[12]
	N	11.8	N/A	
PBBTL:BBL 3:1	P	2.72	0.022	[13]
	N	1.36	0.008	
P(TII-2FT)	P	158.6	1.07	[14]
	N	147.4	1.80	
P(TII-T)	P	179.7	0.78	
	N	21.4	0.08	
P(TII-2ClT)	P	133.8	0.54	
	N	111.3	0.30	
gIDT-BBT	P	42.31	N/A	[15]
	N	0.72	N/A	
p(gDPP-V)	P	104.0	1.61	[16]
	N	102.0	0.98	
DHF-gTT	P	12.5	0.298	[17]
	N	14.0	0.134	
DH-gTT	P	5.6	0.078	

	N	5.4	0.040	
2gDPP-RD-V	P	169.6	1.01	[18]
	N	360.0	0.89	
P(bgTBDOPV-T)	P	349.0	3.46	
	N	84.0	0.62	
P(bgTBDOPV-MeOT2)	P	219.0	1.78	[19]
	N	62.4	0.37	
P(bgTBDOPV-EDOT)	P	235.0	1.57	
	N	98.0	0.45	
H-gDPP	P	359.8	1.34	This work
	N	423.3	2.24	
M-gDPP	P	120.8	0.83	This work
	N	268.4	1.83	
S-gDPP	P	83.9	0.76	This work
	N	229.6	1.76	

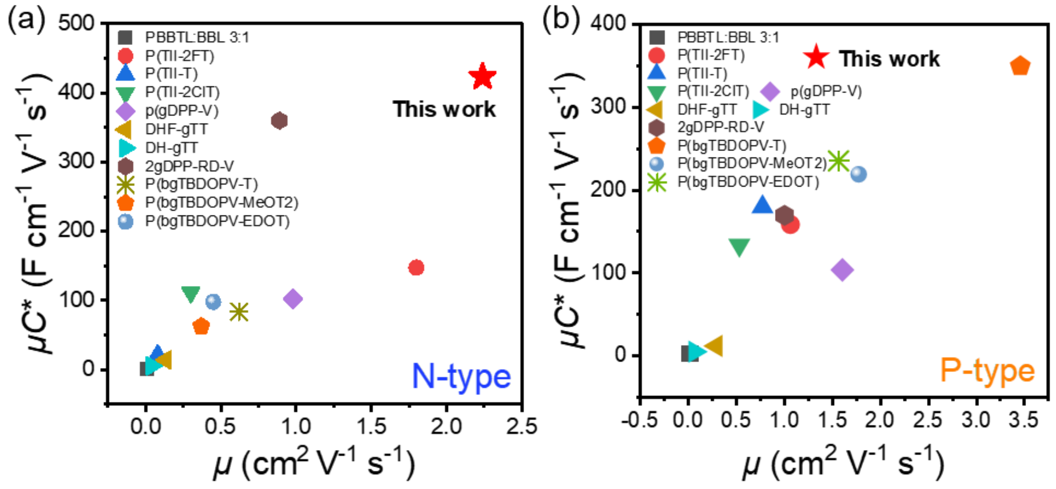

Figure S30. Performance comparison between H-gDPP in this work and state-of-the-art ambipolar OMIECs reported in the literature.

Table S6. The summary of inverters based on planar ambipolar OECTs.

**Materials**	**Gain (V/V)**	V_{DD}	**Ref.**
2DPP-OD-TEG	50	1.4	[9]
P(C_4-T2-C0-EG)	28	0.8	[11]
PrC60MA:p(g2T-TT) (95:5)	82, 62	0.9, −0.8	[12]
PBBTL:BBL (3:1)	31, 42	0.6, −0.6	[13]
gIDT-BBT	28	0.9	[15]
DH-gTT	31.4	0.8	[17]
DHF-gTT	102	0.8	
2gDPP-RD-V	135	0.9	[18]
P(bgTBDOPV-T)	34	0.8	[19]
P(bgTBDOPV-MeOT2)	157	0.8	
P(bgTBDOPV-EDOT)	173	0.8	
P-30	22.5	0.9	[20]
P-40	17.5	0.9	
P-50	18.5	0.9	
P-60	27.4	0.9	
H-gDPP	176	1.1	This work

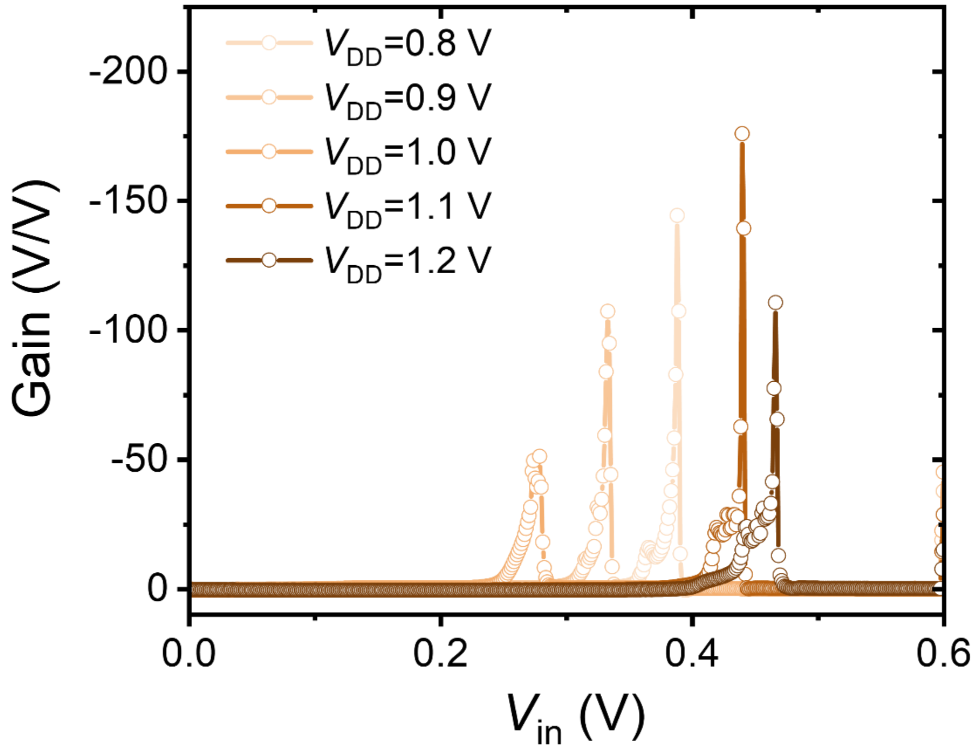

Figure S31. Gain curves of the H-gDPP-based ambipolar inverter.

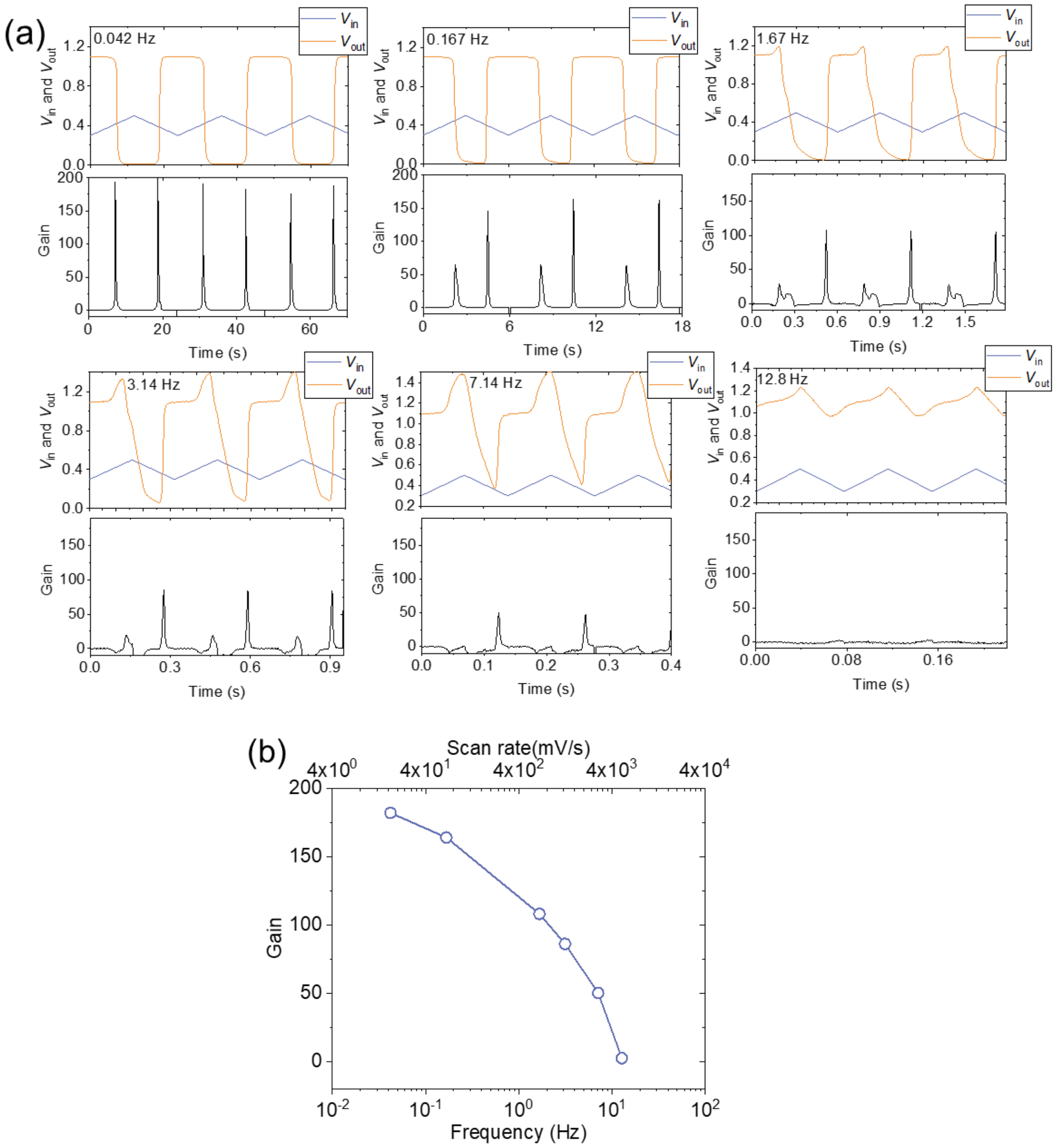

Figure S32. Gain values of H-gDPP-based ambipolar inverter with different scanning rates.

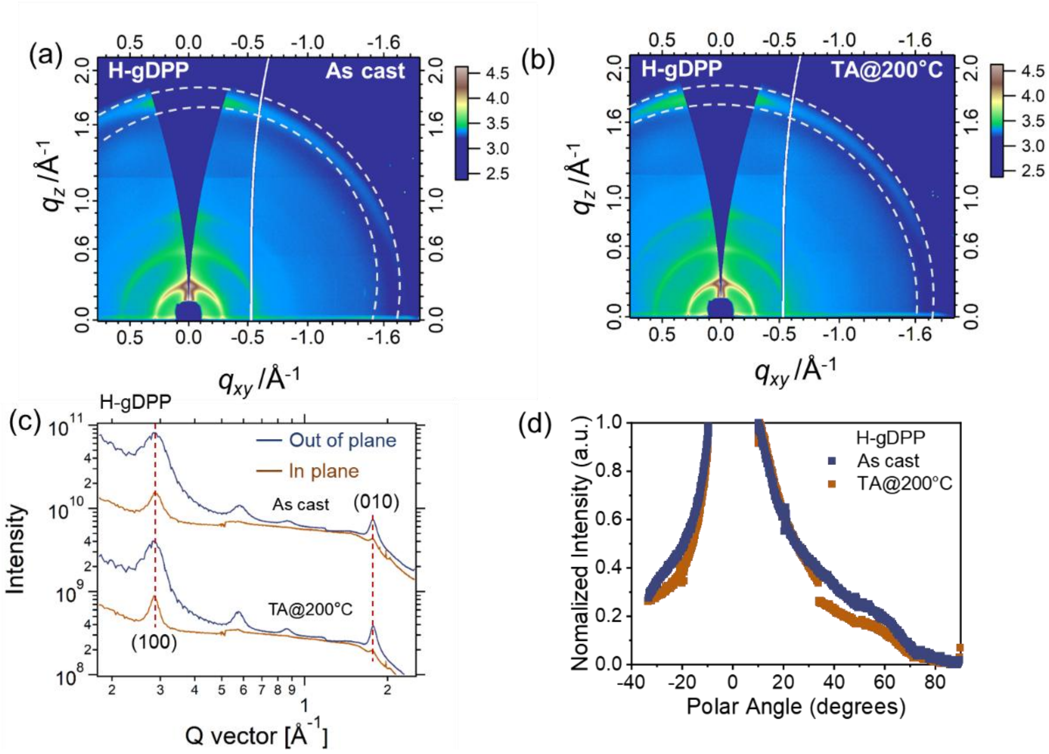

Figure S33. (a-b) Two-dimensional GIWAXS patterns of as-cast and 200 °C annealed H-gDPP polymer films. (c) One-dimensional GIWAXS scattering profile along the in-plane and out-of-plane directions. (d) Pole figure analysis of (010) peak.

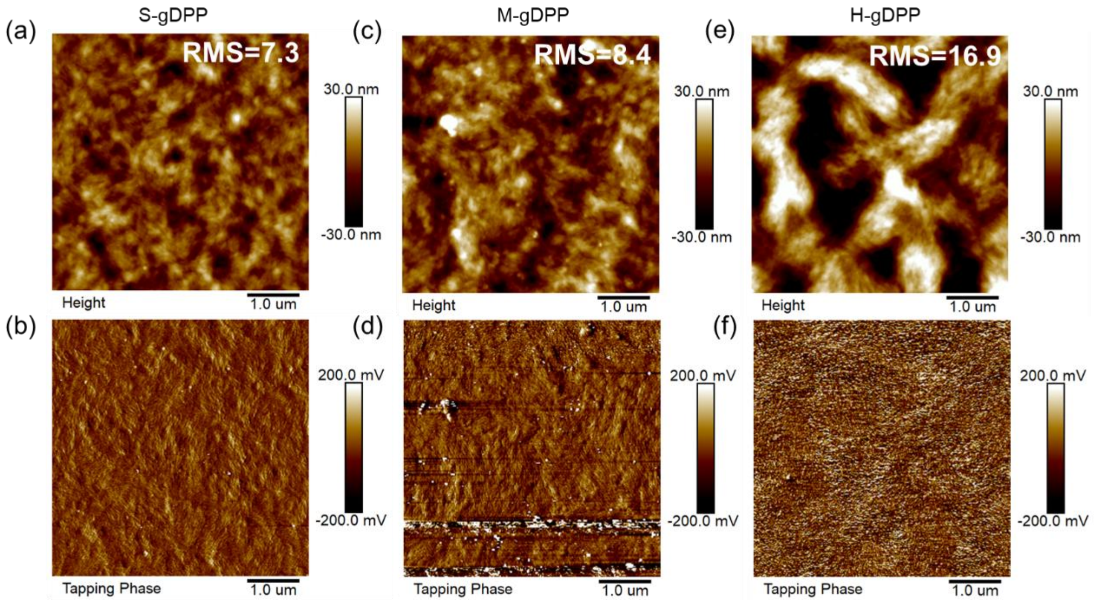

Figure S34. AFM images of (a-b) S-gDPP, (c-d) M-gDPP and (e-f) H-gDPP.

Table S7. (010) peak *d*-spacing, area and face-on orientation ratios of HOMO-gDPP materials with different molecular weights.

	S-gDPP (TA@200°C)	M-gDPP (TA@200°C)	H-gDPP (as cast)	H-gDPP (TA@200°C)
(010) *d*-spacing (Å)	3.56	3.54	3.56	3.54
(010) peak area (a.u.)	7.17×10^{7}	1.42×10^{8}	2.55×10^{8}	3.06×10^{8}
0-45°integral area /0-90°integral area	22.87/33.00	30.31/42.04	19.96/25.38	19.21/22.48
Face-on Population	69.3%	72.1%	78.6%	85.5%

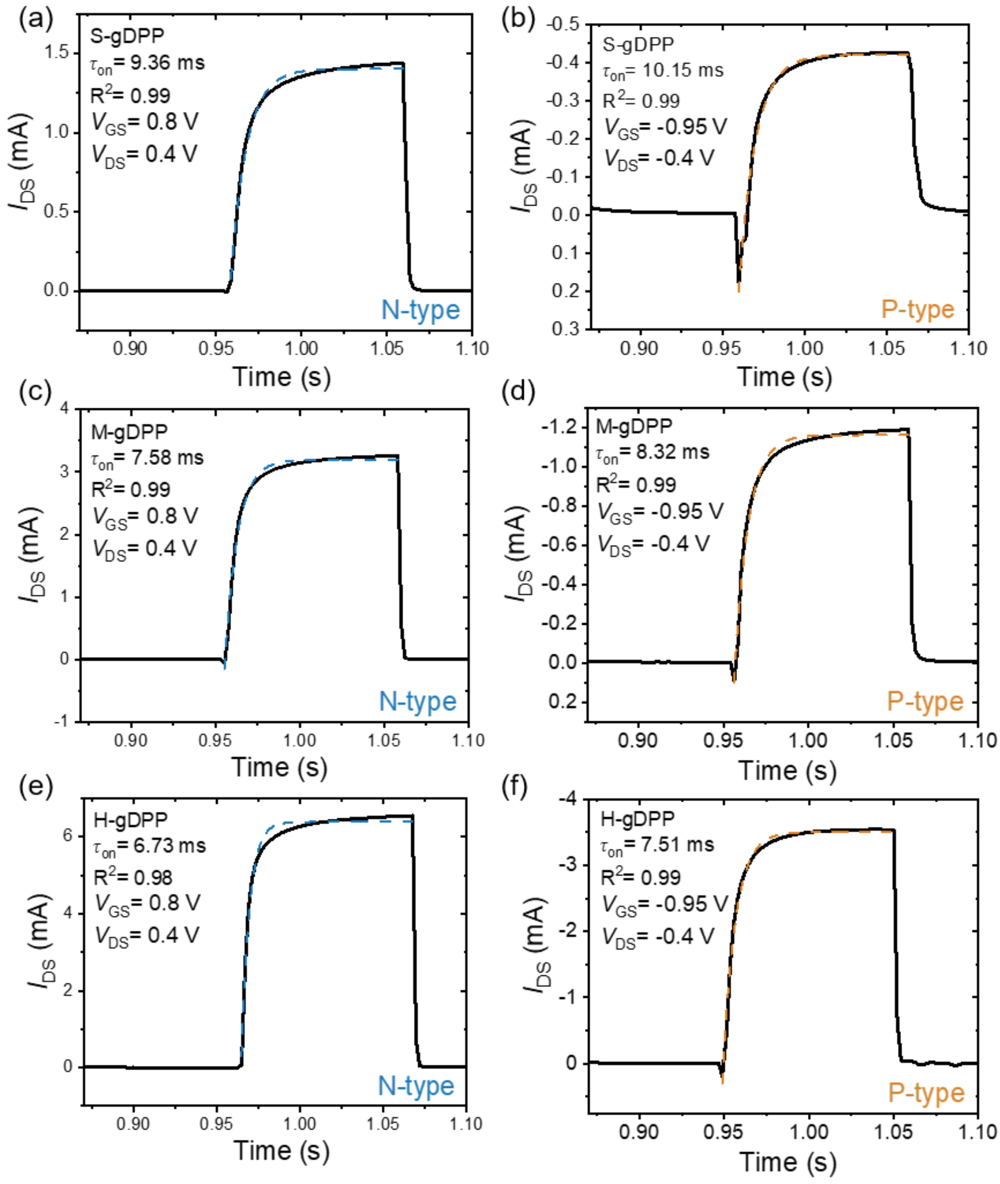

Figure S35. N-type and P-type transient response behaviors of three HOMO-gDPP polymers with different molecular weights (W=1000 μm, L=50 μm, d≈130 nm/130 nm/180 nm for S-gDPP/M-gDPP/H-gDPP).

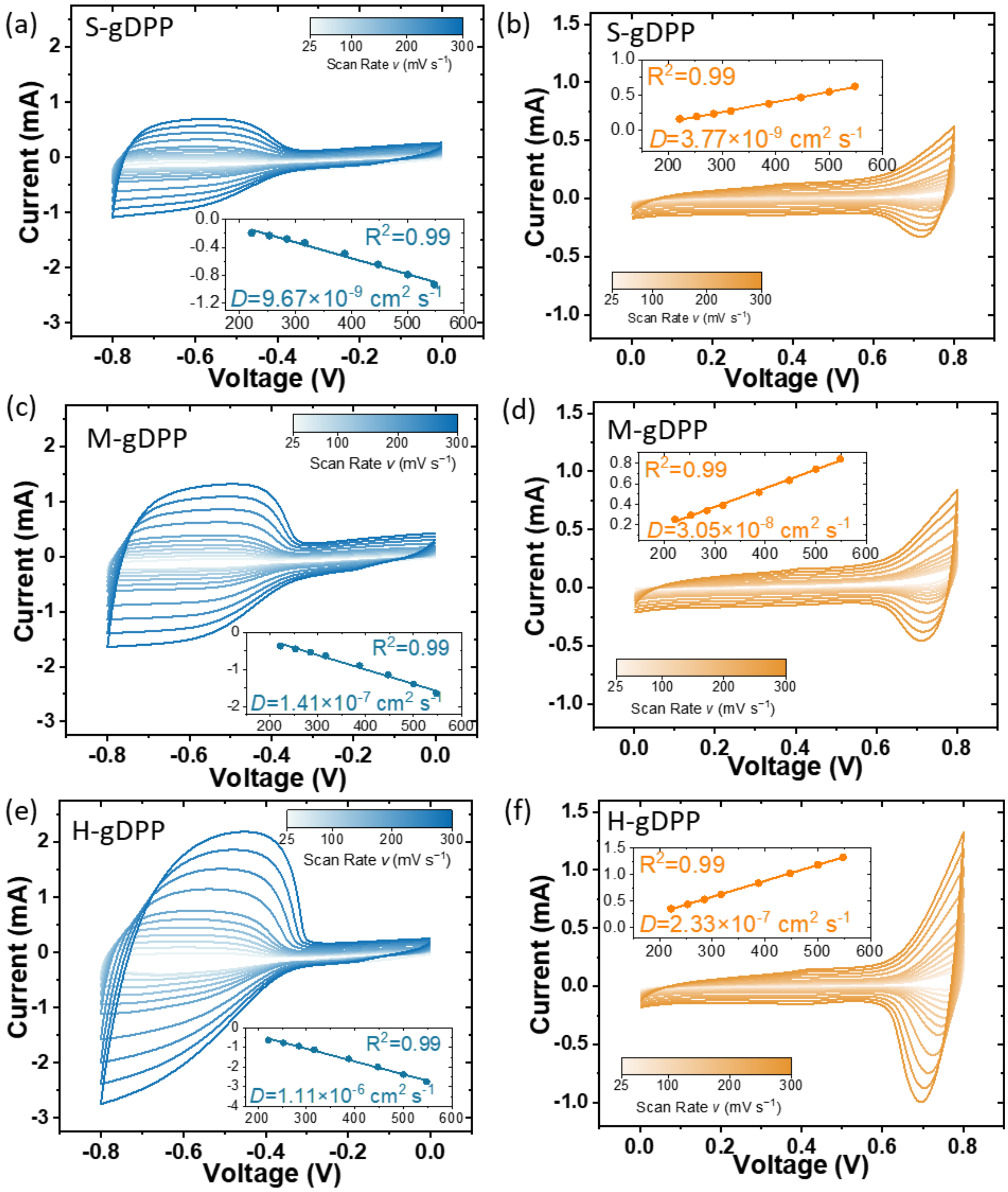

Figure S36. N-type (blue line) and P-type (orange line) scan rate-dependent CV curves of three HOMO-gDPP thin films with different molecular weights. The insets show the linear relationship between peak current and the square root of scan rate. The extracted ion diffusion coefficients (*D*) are also labeled.

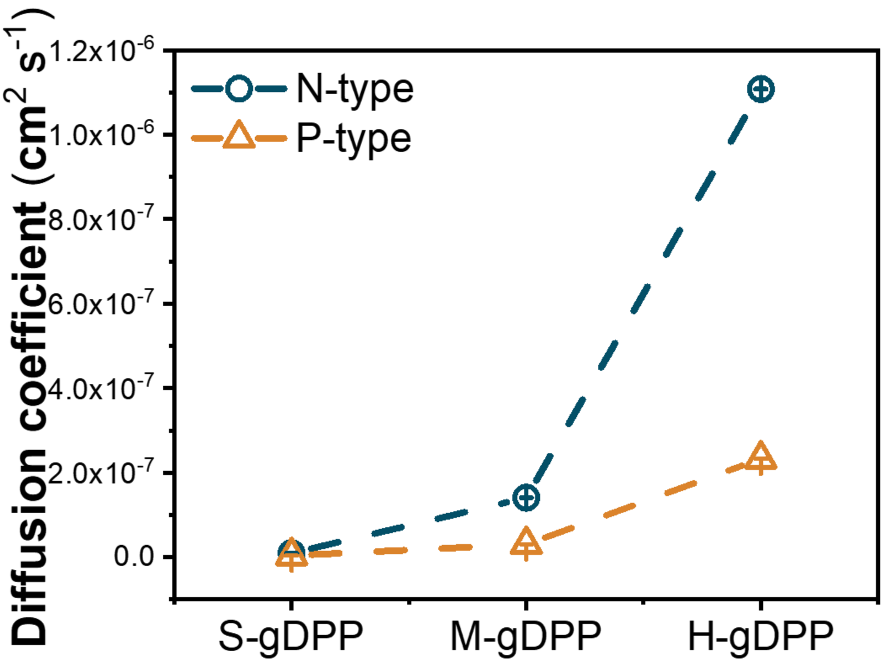

Figure S37. Variation of anion/cation diffusion coefficients (*D*) with molecular weights.

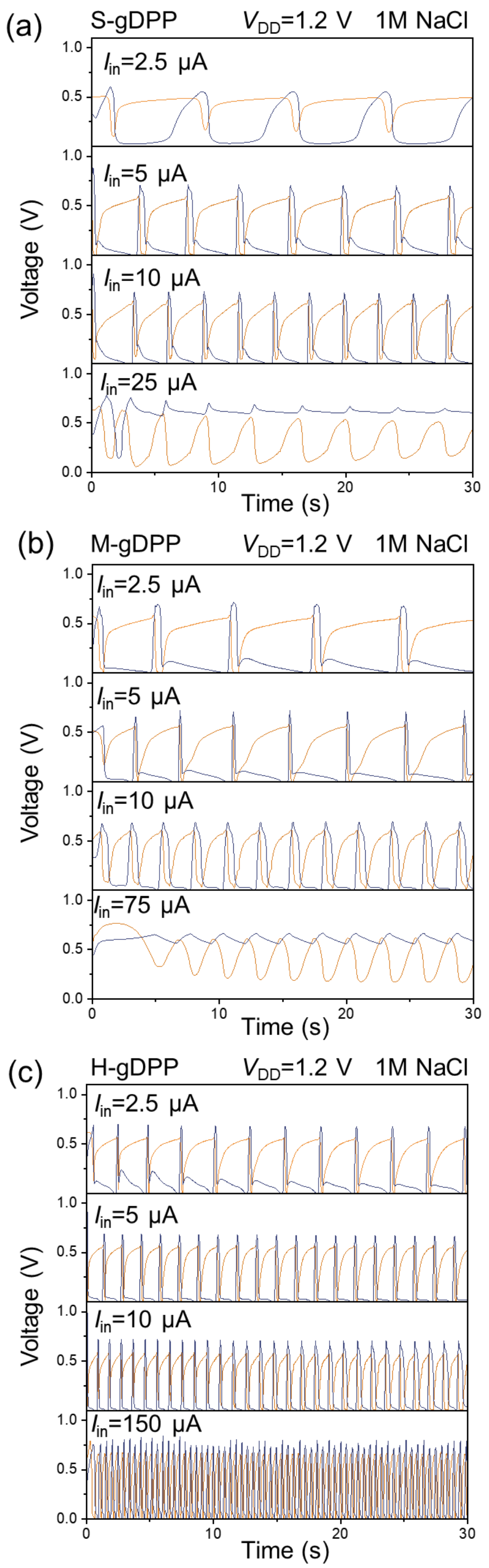

Figure S38. Input current-dependent spiking frequency characteristics of homogeneous ANC based on HOMO-gDPP with different molecular weights (W=1000 μm, L=50 μm, d≈130 nm).

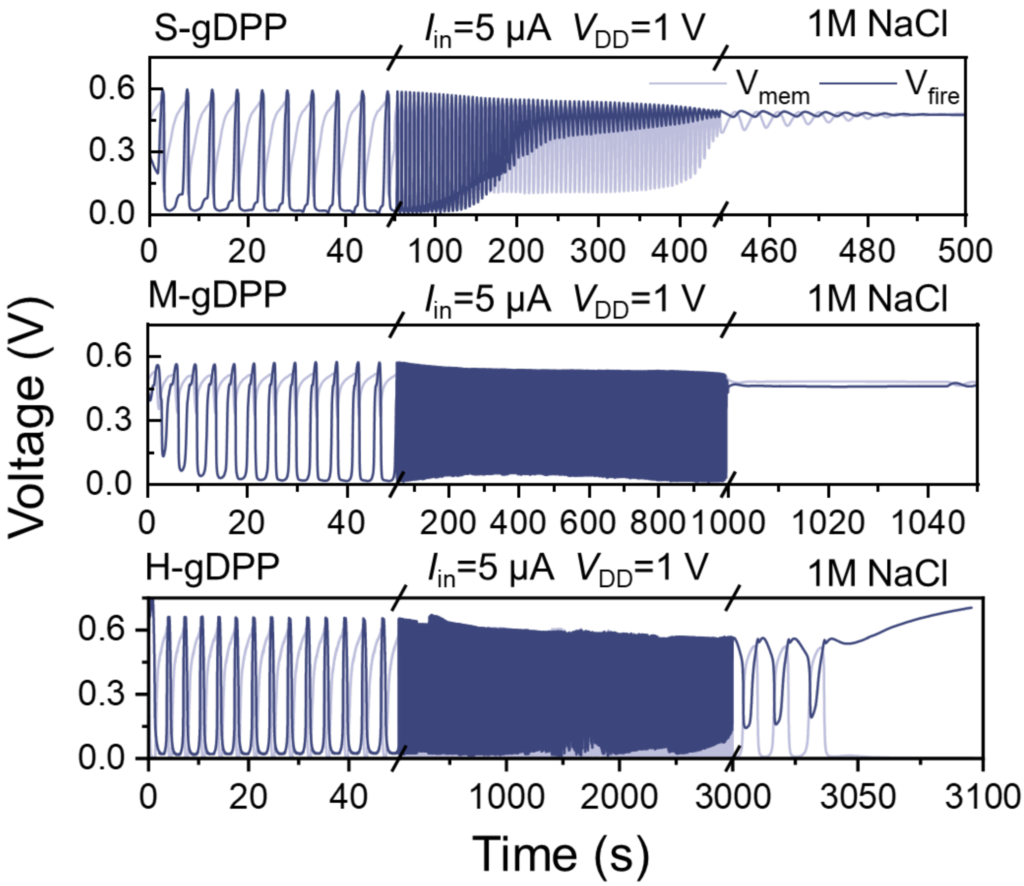

Figure S39. Cycling stability comparison of homogeneous ANC constructed with three HOMO-gDPP polymers with different molecular weights.

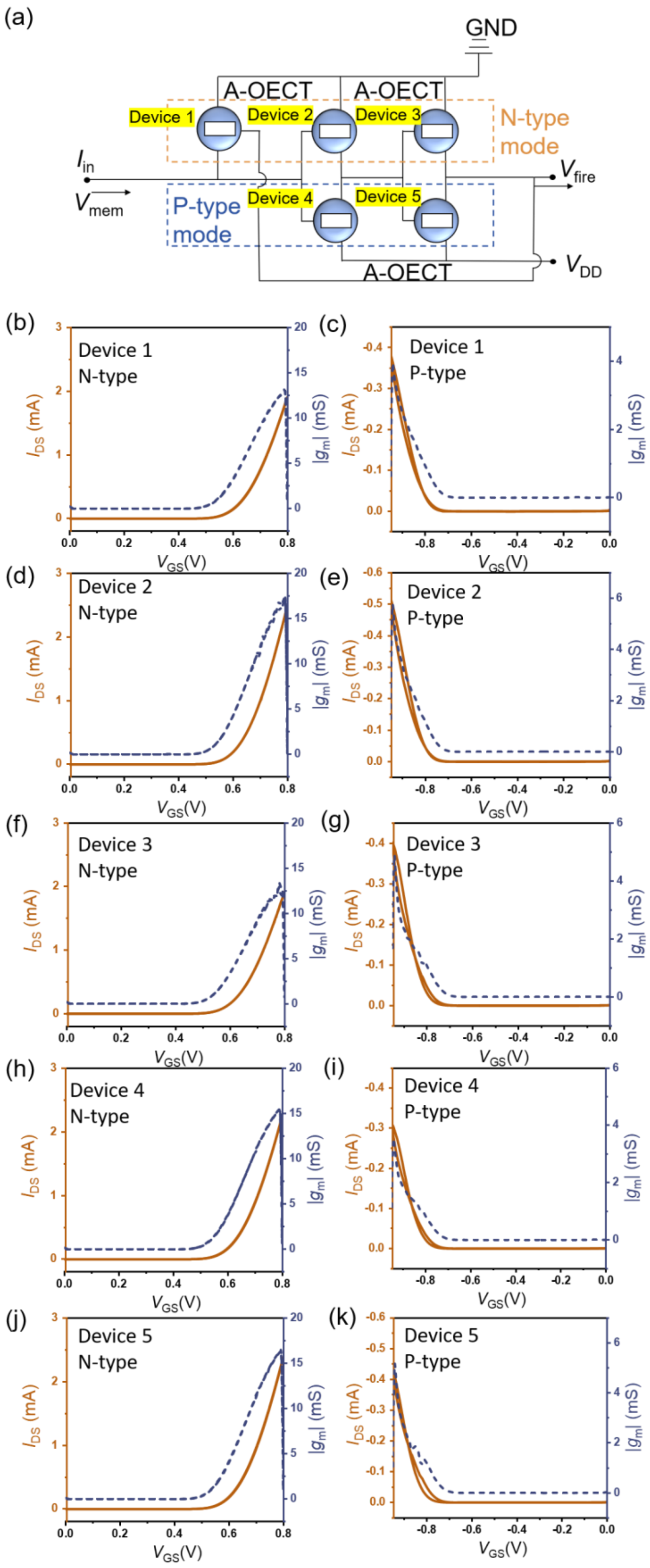

Figure S40. N-type and P-type transfer characteristics of five OECTs in the S-gDPP-based-ANC before the test.

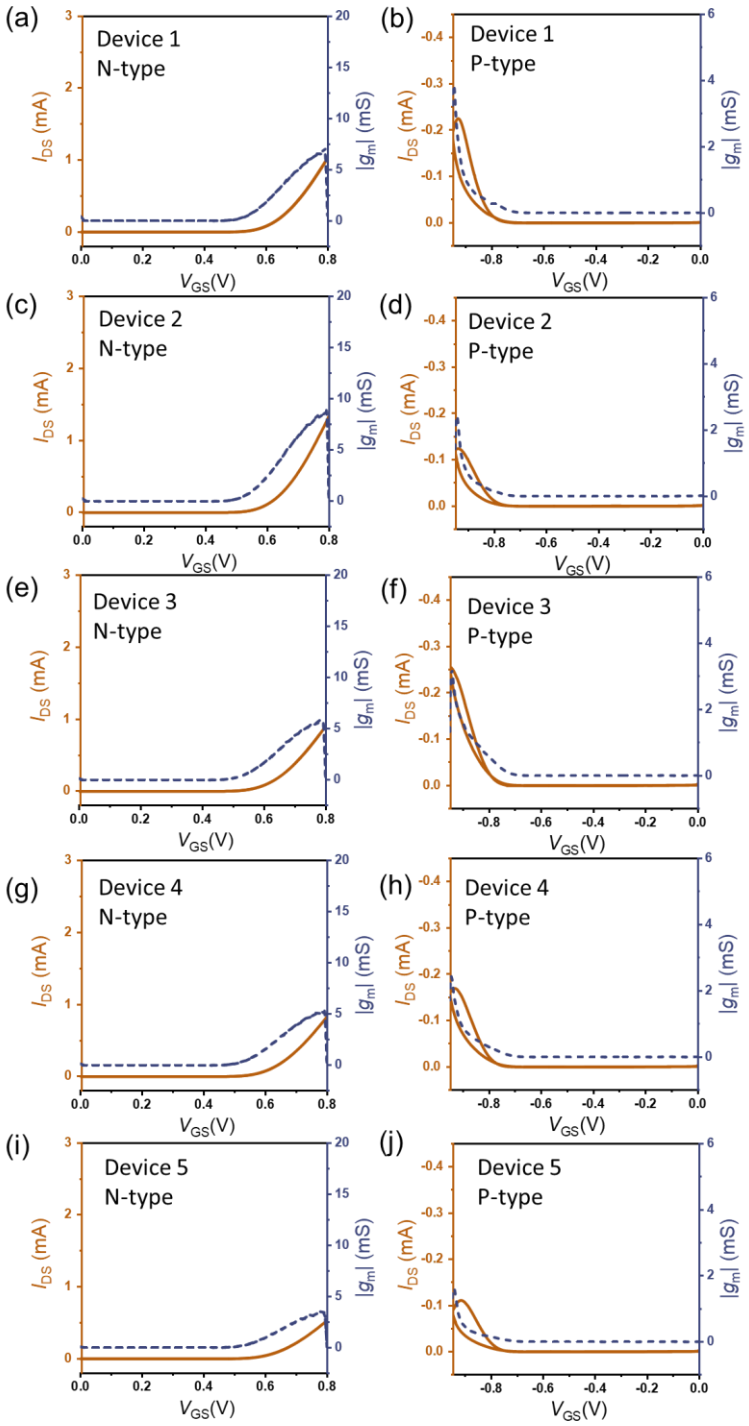

Figure S41. N-type and P-type transfer characteristics of five OECTs in the S-gDPP-based-ANC after 200 seconds of test.

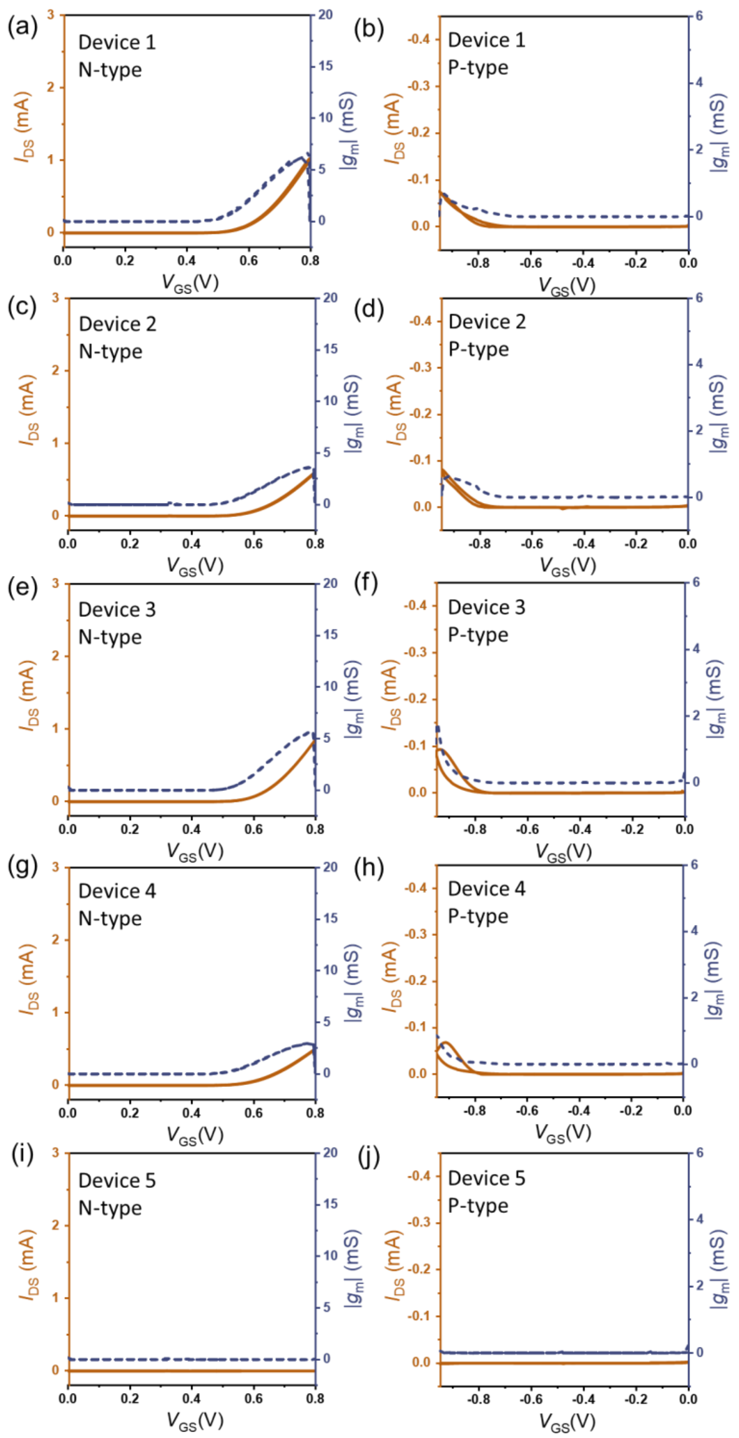

Figure S42. N-type and P-type transfer characteristics of five OECTs in the S-gDPP-based-ANC after circuit failure.

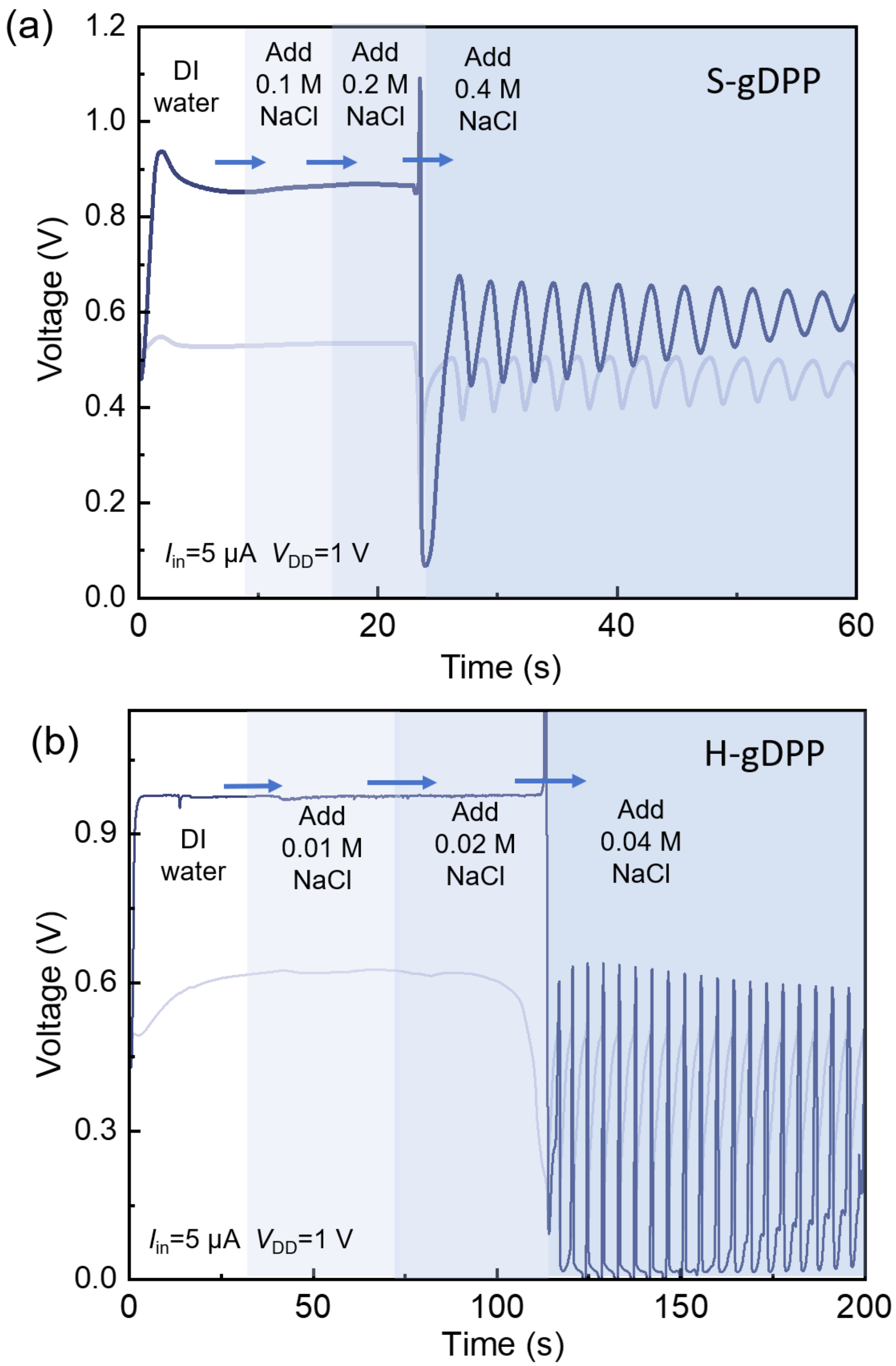

Figure S43. Chemical sensitivity of S-gDPP and H-gDPP-based ANCs.

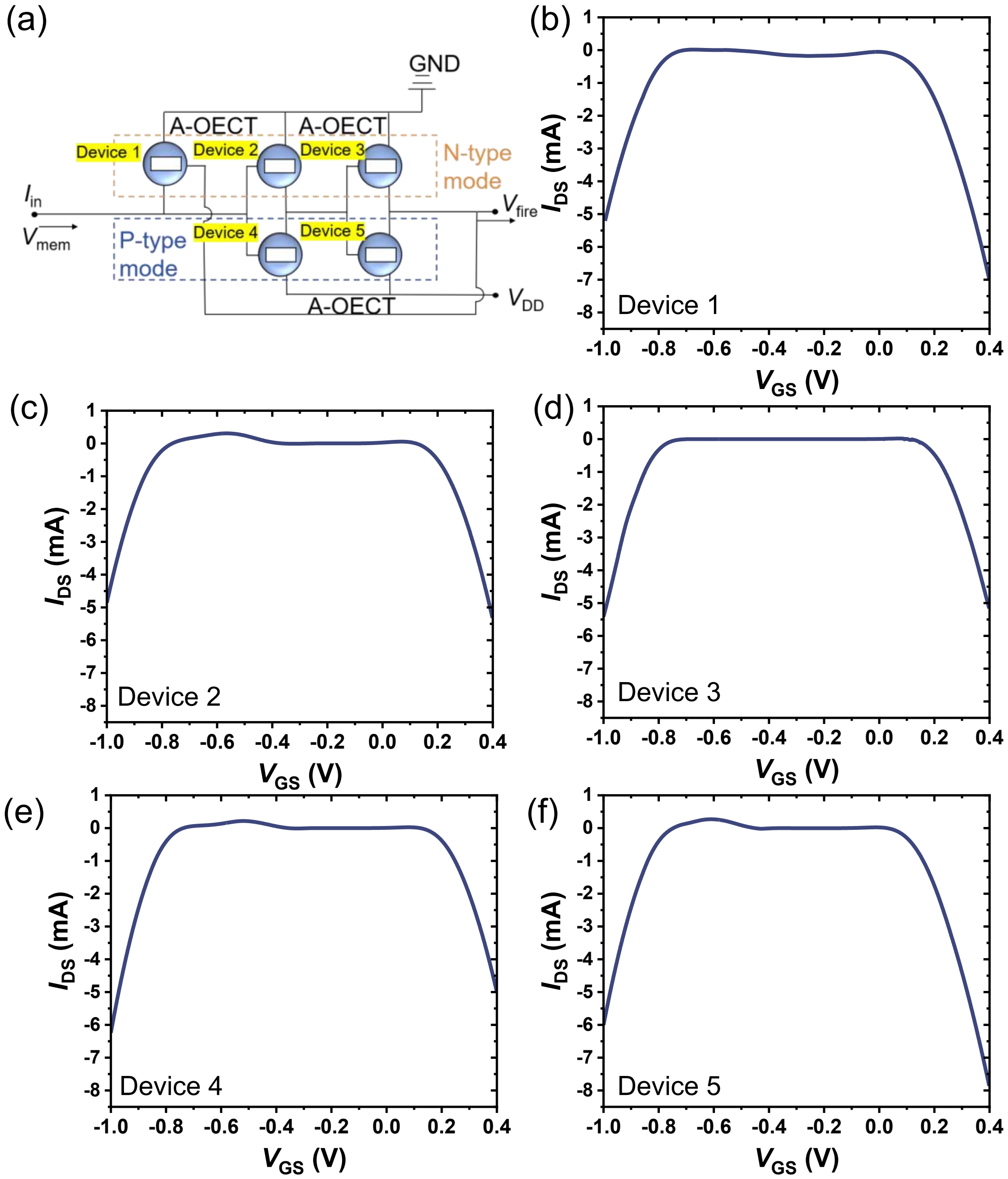

Figure S44 Ambipolar characteristics of five individual OECTs in the H-gDPP-based ANC (ANC 1).

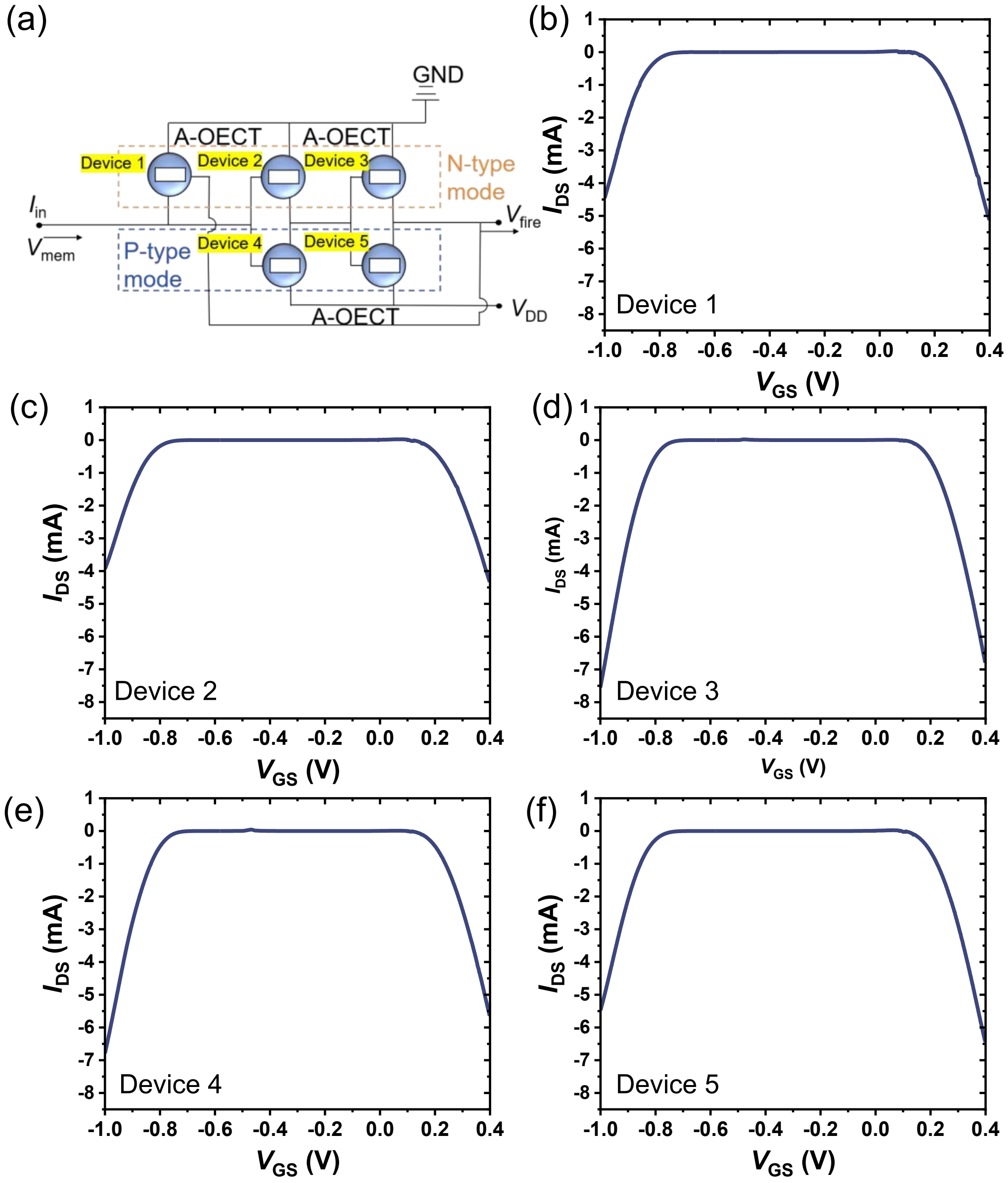

Figure S45. Ambipolar characteristics of five individual OECTs in the H-gDPP-based ANC (ANC 2).

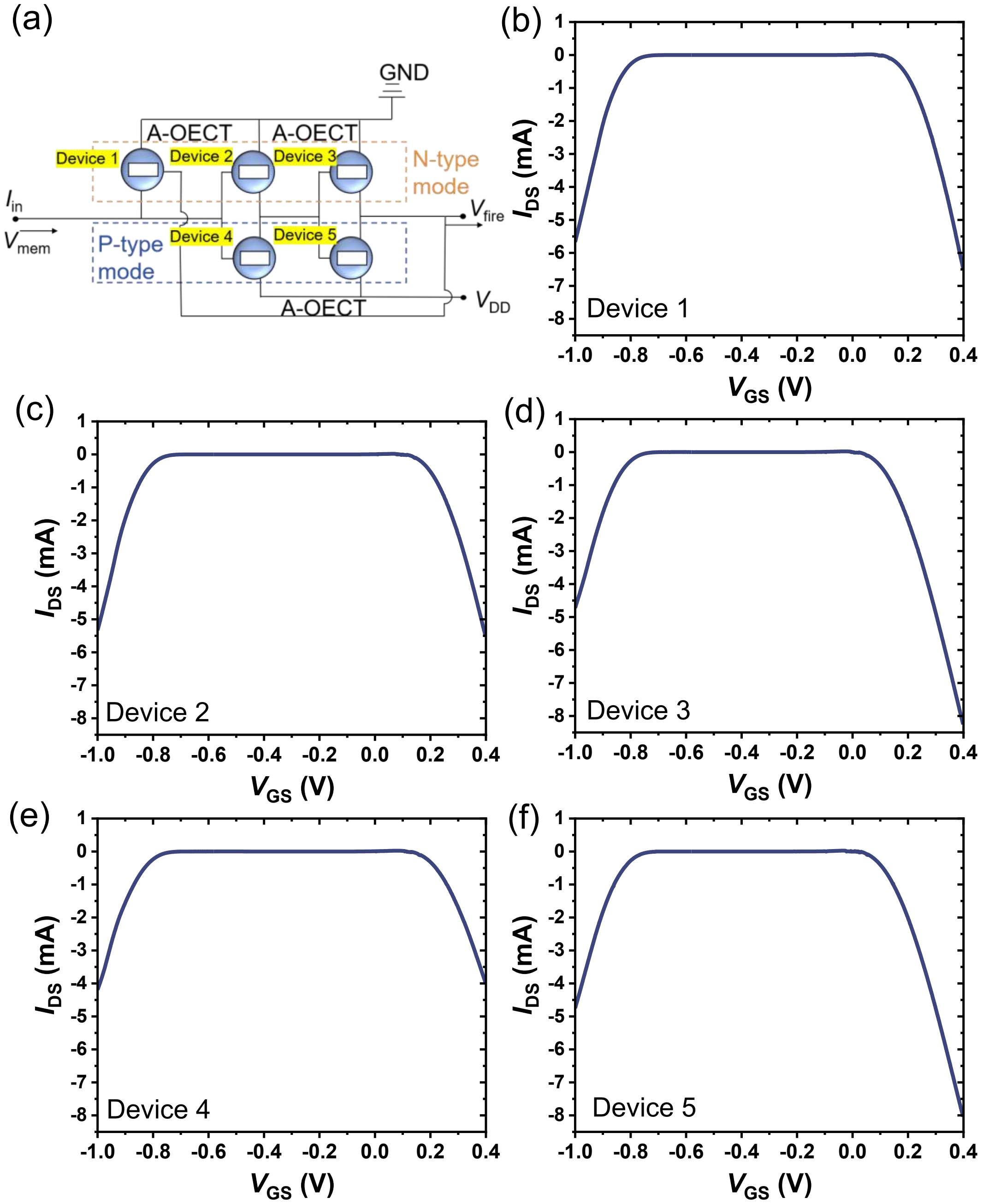

Figure S46. Ambipolar characteristics of five individual OECTs in the H-gDPP-based ANC (ANC 3).

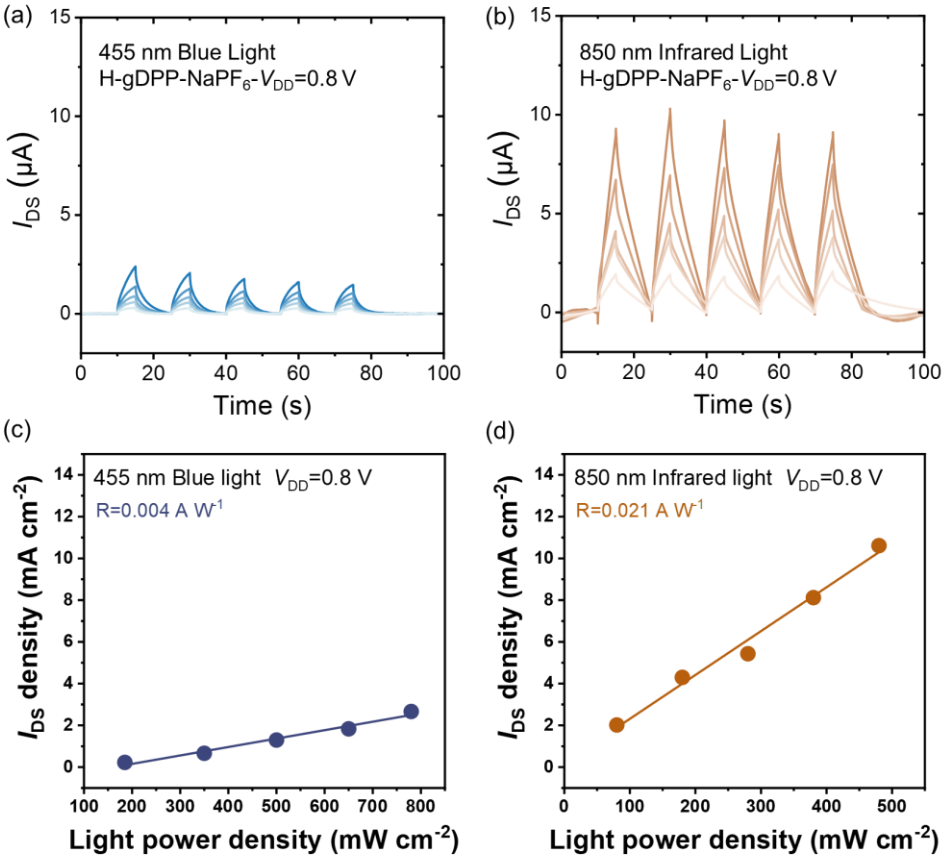

Figure S47. Photoresponse data of the planar OECT with 1M $NaPF_6$ electrolyte.

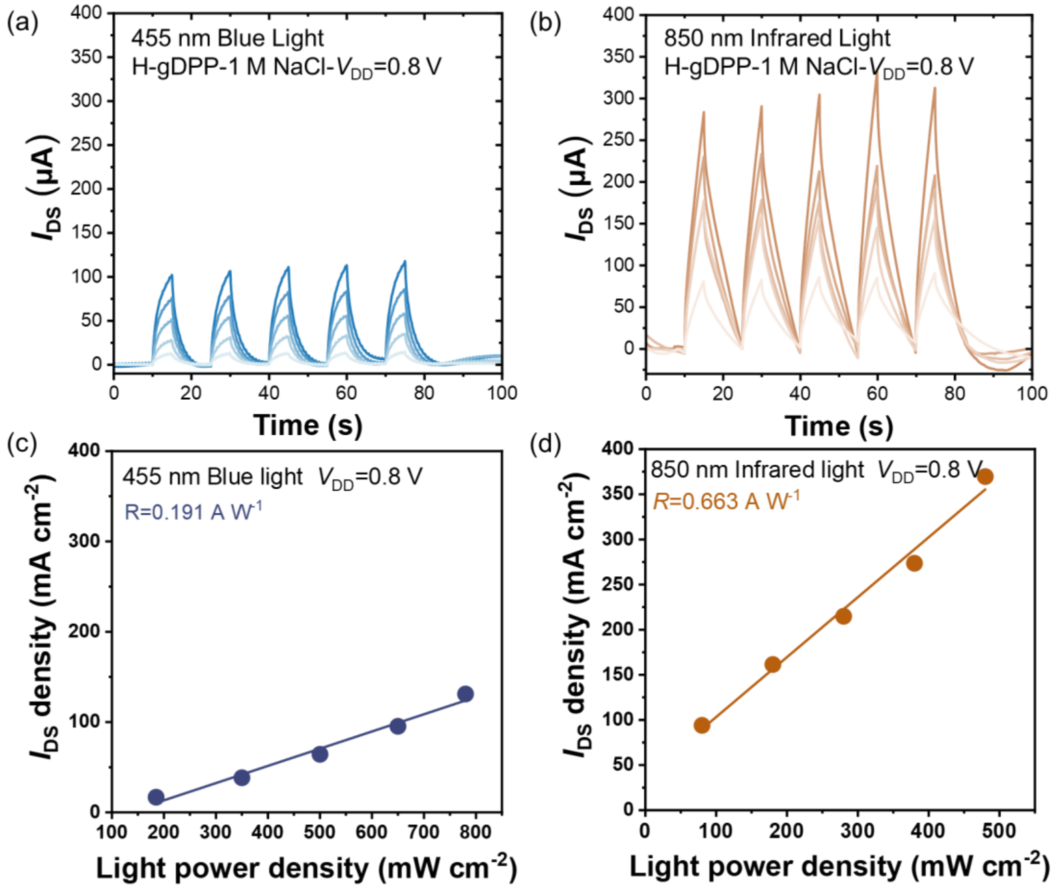

Figure S48. Photoresponse data of the vertical OECT with 1M NaCl electrolyte**.**

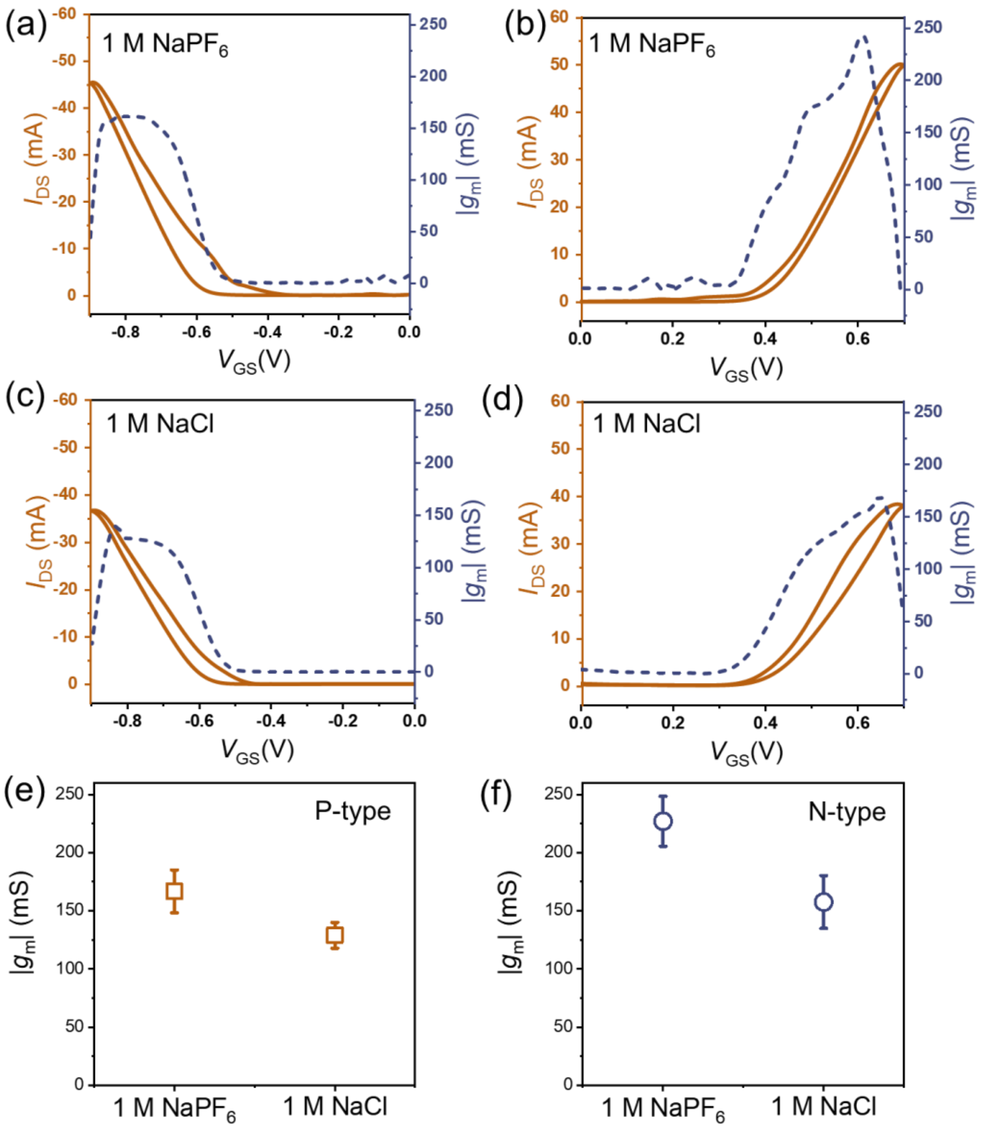

Figure S49. (a-d) Transfer curves and transconductance of H-gDPP-based vertical OECTs with two different electrolytes (1 M $NaPF_6$ and 1 M NaCl). (e-f) Statistical comparison of N/P-type transconductance in different electrolytes. Error bars denote the s.d. for $n = 4$ devices, mean ± s.d.

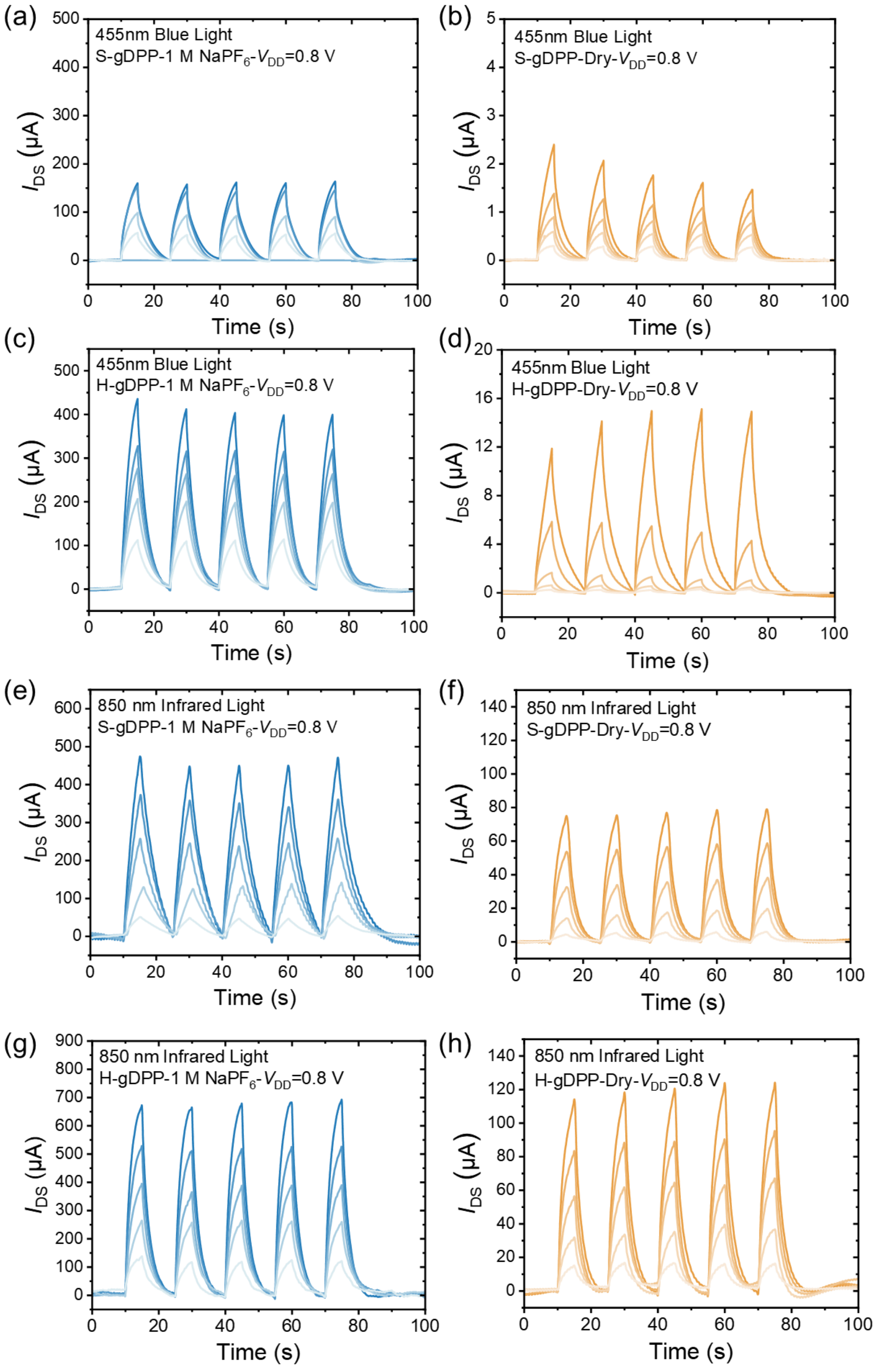

Figure S50. Schematic illustration of photoresponse characteristics for H-gDPP and S-gDPP materials under 1 M $NaPF_6$ electrolyte and electrolyte-free (dry) conditions upon 850 nm infrared and 455 nm blue light illumination. The gradient-colored curves (from dark to light) represent light intensities of 100%, 80%, 60%, 40%, and 20%, respectively.

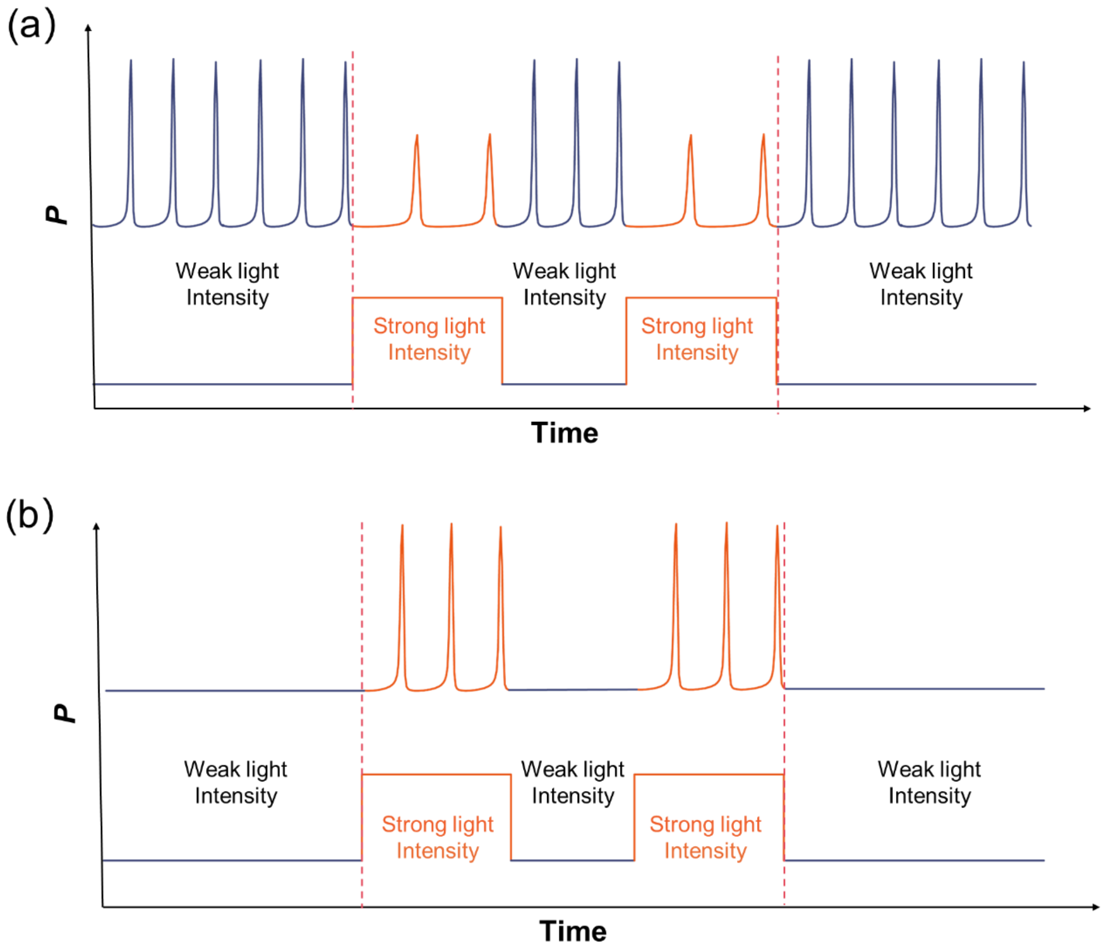

Figure S51. The ideal adaptive response of inhibitory ANC (a) and excitatory ANC (b) to the change of light intensity.

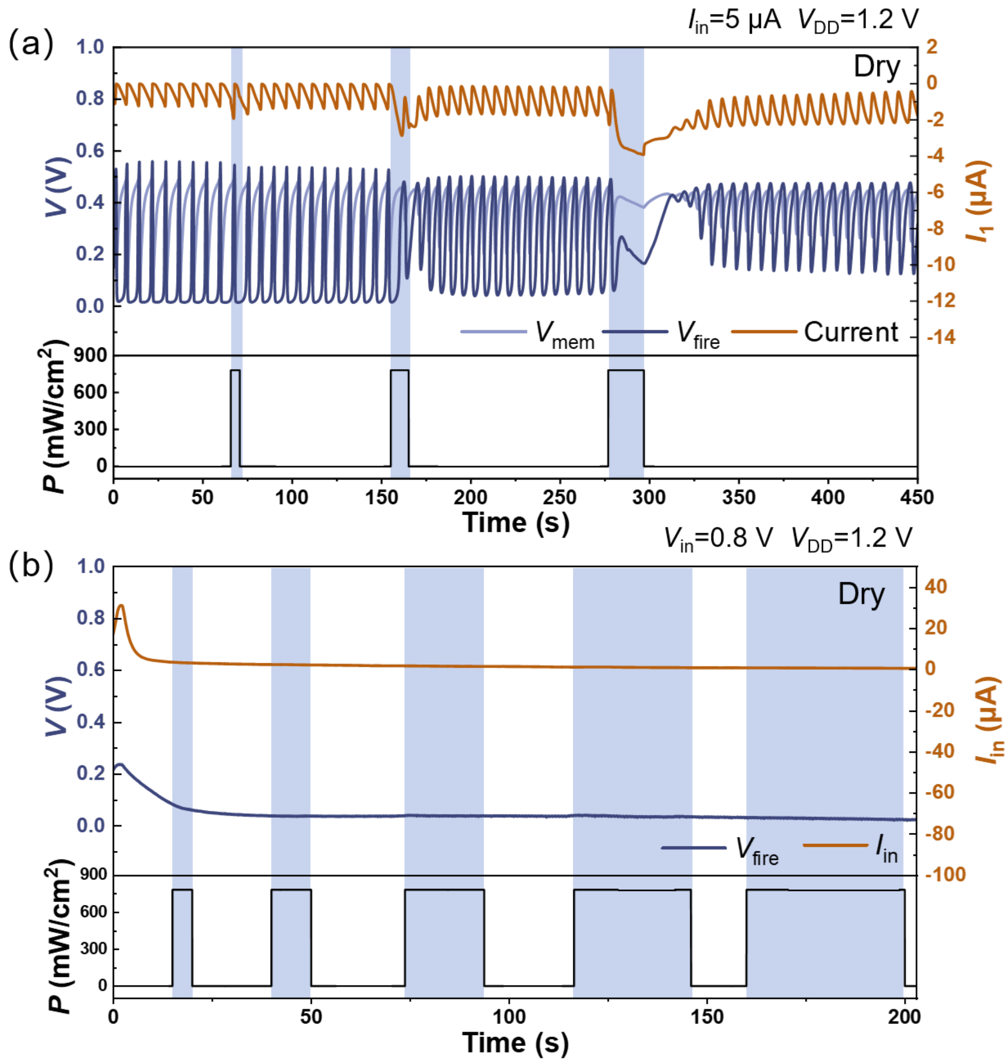

Figure S52. Dynamic response of optic ANC upon light stimuli, tested without electrolyte. (a) Inhibitory ANC. (b) Excitatory ANC.

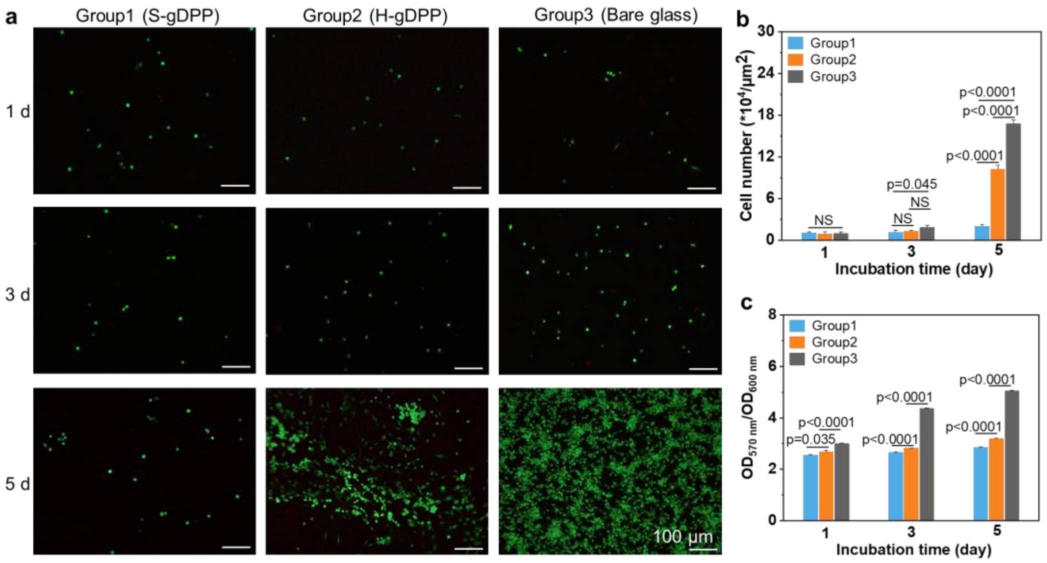

Figure S53 Biocompatibility test of different parts in the integrated device. (a) Fluorescence microscope images of the PC-12 cells cultured on different device-cell interfaces. (**b-c**) Cell density (b) and 3-(4,5-Dimethylthiazol-2-yl)-2,5-diphenyltetrazolium bromide (MTT) tested mitochondrial activities (c) on different device-cell interfaces.

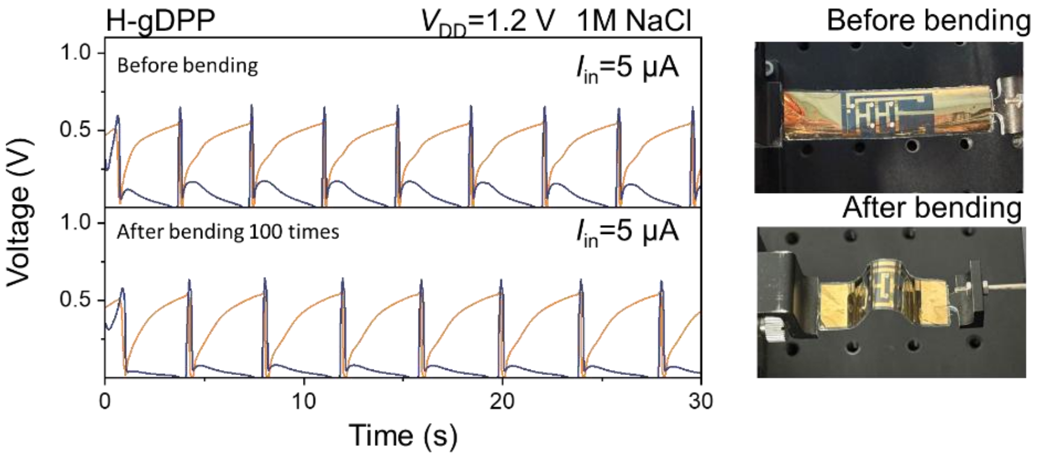

Figure S54 Spiking behavior of the H-gDPP ANC on Parylene substrate before and after 100 times of bending.

References

1. Hexemer A, Bras W, Glossinger J et al. A SAXS/WAXS/GISAXS beamline with multilayer monochromator. *J Phys: Conf Ser* 2010; **247:** 012007.
2. Ilavsky J. Nika: software for two-dimensional data reduction. *J Appl Crystallogr* 2012; **45:** 324–8.
3. Randles JEB. A cathode ray polarograph. Part II.—The current-voltage curves. *Trans Faraday Soc* 1948; **44:** 327–38.
4. Ševčík A. Oscillographic polarography with periodical triangular voltage. *Collect Czechoslov Chem Commun* 1948; **13:** 349–77.
5. Lin S, Usov PM, Morris AJ. The role of redox hopping in metal-organic framework electrocatalysis. *Chem Commun* 2018; **54:** 6965–74.
6. Bernards DA and Malliaras GG. Steady-state and transient behavior of organic electrochemical transistors. *Adv Funct Mater* 2007; **17:** 3538–44.
7. Ohayon D, Druet V, Inal S. A guide for the characterization of organic electrochemical transistors and channel materials. *Chem Soc Rev* 2023; **52:** 1001–23.
8. Giovannitti A, Nielsen CB, Sbircea D-T *et al.* N-type organic electrochemical transistors with stability in water. *Nat Commun* 2016; **7:** 13066.
9. Samuel JJ, Garudapalli A, Mohapatra AA *et al.* Single-Component CMOS-Like Logic using Diketopyrrolopyrrole-Based Ambipolar Organic Electrochemical Transistors. *Adv Funct Mater* 2021; **31:** 2102903.
10. Rashid RB, Du W, Griggs S *et al.* Ambipolar inverters based on cofacial vertical organic electrochemical transistor pairs for biosignal amplification. *Sci Adv* 2021; **7:** eabh1055.
11. Ohayon D, Savva A, Du W *et al.* Influence of Side Chains on the n-Type Organic Electrochemical Transistor Performance. *ACS Appl Mater Interfaces* 2021; **13:** 4253–66.
12. Stein E, Nahor O, Stolov M *et al.* Ambipolar blend-based organic electrochemical transistors and inverters. *Nat Commun* 2022; **13:** 5548.
13. Wu X, Tam TLD, Chen S *et al.* All-Polymer Bulk-Heterojunction Organic

Electrochemical Transistors with Balanced Ionic and Electronic Transport. *Adv Mater* 2022; **34**: 2206118.

14. Ge G-Y, Xu J, Wang X *et al.* On-site biosignal amplification using a single high-spin conjugated polymer. *Nat Commun* 2025; **16:** 396.
15. Sun Y, Lan Y, Li M *et al.* Indacenodithiophene-based single-component ambipolar polymer for high-performance vertical organic electrochemical transistors and inverters. *Aggregate* 2024; **5:** e577.
16. Cong S, Chen J, Xie M *et al.* Single ambipolar OECT-based inverter with volatility and nonvolatility on demand. *Sci Adv* 2024; **10:** eadq9405.
17. Qi G, Wang M, Wang S *et al.* High-Performance, Single-Component Ambipolar Organic Electrochemical Transistors with Balanced n/p-Type Properties for Inverter and Biosensor Applications. *Adv Funct Mater* 2024; **35:** 2413112.
18. Lan L, Wang Y, Zhu X *et al.* Ultrathin-Film Small Molecule Mixed Conductors Exhibiting Ion-Tunable Ambipolarity for High-Performance Organic Electrochemical Transistors and Multivalued Logic Inverters. *Adv Mater* 2025; **37:** 2501041.
19. Pan X, Ren Z, Chen Y *et al.* Strong Proquinoidal Acceptor Enables High-Performance Ambipolar Organic Electrochemical Transistors. *Adv Mater* 2025; **37:** 2417146.
20. Pan T, Jiang X, van Doremaele ERW *et al.* Over 60 h of Stable Water-Operation for N-Type Organic Electrochemical Transistors with Fast Response and Ambipolarity. *Adv Sci* 2024; **11:**2400872.
